# Machine Learning and Deep Learning Approaches for Arabic Sign Language Recognition: A Decade Systematic Literature Review

**DOI:** 10.3390/s24237798

**Published:** 2024-12-05

**Authors:** Asmaa Alayed

**Affiliations:** Department of Software Engineering, College of Computing, Umm Al-Qura University, Makkah 21955, Saudi Arabia; asayed@uqu.edu.sa

**Keywords:** Arabic sign language (ArSL), Arabic sign language recognition (ArSLR), dataset, machine learning, deep learning, hand gesture recognition

## Abstract

Sign language (SL) is a means of communication that is used to bridge the gap between the deaf, hearing-impaired, and others. For Arabic speakers who are hard of hearing or deaf, Arabic Sign Language (ArSL) is a form of nonverbal communication. The development of effective Arabic sign language recognition (ArSLR) tools helps facilitate this communication, especially for people who are not familiar with ArSLR. Although researchers have investigated various machine learning (ML) and deep learning (DL) methods and techniques that affect the performance of ArSLR systems, a systematic review of these methods is lacking. The objectives of this study are to present a comprehensive overview of research on ArSL recognition and present insights from previous research papers. In this study, a systematic literature review of ArSLR based on ML/DL methods and techniques published between 2014 and 2023 is conducted. Three online databases are used: Web of Science (WoS), IEEE Xplore, and Scopus. Each study has undergone the proper screening processes, which include inclusion and exclusion criteria. Throughout this systematic review, PRISMA guidelines have been appropriately followed and applied. The results of this screening are divided into two parts: analysis of all the datasets utilized in the reviewed papers, underscoring their characteristics and importance, and discussion of the ML/DL techniques’ potential and limitations. From the 56 articles included in this study, it was noticed that most of the research papers focus on fingerspelling and isolated word recognition rather than continuous sentence recognition, and the vast majority of them are vision-based approaches. The challenges remaining in the field and future research directions in this area of study are also discussed.

## 1. Introduction

Sign language (SL) is a powerful means of communication among humans. It is used by people who are deaf or hard of hearing, as well as those who struggle with oral speech due to disability or special conditions. According to the latest estimations, as of March 2021, there are over 1.5 billion people worldwide experiencing some degree of hearing loss, which could increase to 2.5 billion by 2050 [1]. Around 78 million people with hearing difficulties were estimated in the Eastern Mediterranean region, which could grow to 194 million by 2050 [1].

There are two main classes of sign language symbols: single-handed and double-handed [2]. These signs are further divided into dynamic and static. One dominant hand is used to represent one-handed signs. Any static or dynamic gesture can be used to represent it. Both dominant and non-dominant hands are used when signing to indicate two-handed signs [2]. SL uses non-manual signs (NMS), such as body language, lip patterns, and facial expressions, in addition to manual signs (MS) that use static hand/arm gestures, hand/arm motions, and fingerspelling [3].

Sign language recognition (SLR) is the process of recognizing and deciphering sign language movements or gestures [4]. It usually involves complex algorithms and computational operations. Sign language recognition systems (SLRS) is one application within the field of human-computer interaction (HCI) that interprets sign language of hearing impairments into text or voice of oral language [5]. SLRS can be categorized into three main groups based on the main technique used for gathering data, namely sensor-based or hardware-based, vision-based or image-based, and hybrid [6,7]. Sensor-based techniques use data gloves that the signer wears to collect data about their actions from external sensors. Most of the current research, however, has focused on vision-based approaches that use images, videos, and depth data to extract the semantic meaning of hand signals due to practical concerns with sensor-based techniques [4,6]. Hybrid methods have occasionally been employed to gather information on sign language recognition. Comparing hybrid methods to other approaches, they perform similarly or even better in terms of proportional automatic speech or handwriting recognition. In hybrid techniques, multi-mode information about the hand shapes and movement is obtained by combining vision-based cameras with other types of sensors, like glove sensors.

**Figure 1:**
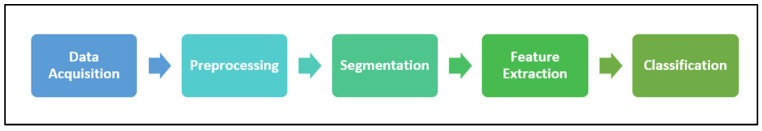
Stages of sign language recognition systems.

As shown in Figure 1, the stages involved in sign language recognition can be broadly divided into data acquisition, pre-processing, segmentation, feature extraction, and classification [8,9]. In data acquisition, single frames of images are the input for static sign language recognition; video, or continuous frames of images, is the input for dynamic signs. In order to enhance the system’s overall performance, the preprocessing stage modifies the image or video inputs. Segmentation, or the partitioning of an image or video into several separate parts, is the third step. A good feature extraction arises from perfect segmentation [10]. Feature extraction is performed to transform important parts of the input data into sets of compact feature vectors. When it comes to the recognition of sign language, the features that are extracted from the input hand gestures should contain pertinent information and be displayed in a condensed form that helps distinguish the sign that needs to be categorized from other signals. The final step is classification. Machine learning (ML) techniques for classification can be divided into two categories: supervised and unsupervised [8]. Through the use of supervised machine learning, a system can be trained to identify specific patterns in input data, which can subsequently be utilized to predict future data. Using labeled training data and a set of known training examples, supervised machine learning infers a function. Inferences are derived from datasets containing input data without labeled responses through the application of unsupervised machine learning. There is no reward or penalty weightage indicating which classes the data should belong to because the classifier receives no labeled responses. Deep learning (DL) approaches have surpassed earlier cutting-edge machine learning techniques in a number of domains in recent years, particularly in computer vision and natural language processing [4]. Eliminating the need to construct or extract features is one of the primary objectives of deep models. Deep learning enables multi-layered computational models to learn and represent data at various levels of abstraction, mimicking the workings of the human brain and implicitly capturing the intricate structures of massive amounts of data.

It is worth noting that there is no international SL. In fact, SL is geography-specific, as it differs from one region or country to another in terms of syntax and grammar [9,11,12,13]. Different sign languages include, for example, American Sign Language (ASL), Australian Sign Language (Auslan), British Sign Language (BSL), Japanese Sign Language (JSL), Urdu Sign Language, Arabic Sign Language (ArSL), and others [9,11,12,13].

**Figure 2:**
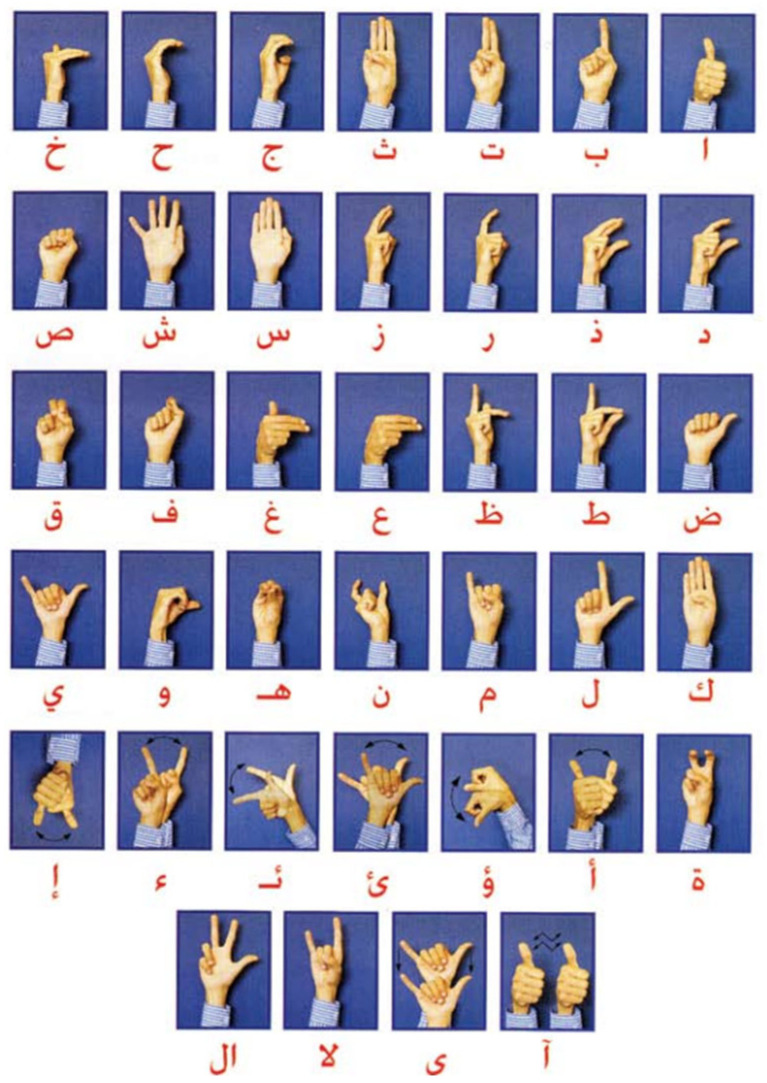
Representation of the Arabic sign language for Arabic alphabets [14].

The language that is spoken throughout the Middle East and North Africa is unified ArSL [2]. Although there are 28 letters in the Arabic alphabet, Arabic sign language uses 39 signs [14,15], as depicted in Figure 2. The eleven extra signs are fundamental signs made up of two letters combined. For example, in Arabic, the two letters “ال” are frequently used, much like “the” in English. Thus, the 39 signs for the Arabic alphabet are used in the majority of reviewed works on Arabic sign language recognition (ArSLR) [9,15].

SLR research efforts are categorized according to a taxonomy that starts with creating systems that can recognize small forms and segments, like alphabets, and progresses to larger but still small forms, like isolated words, and ultimately to the largest and most challenging forms, like complete sentences [4,9,15]. As the unit size to be recognized increases, the ArSLR job becomes harder. Three main categories—fingerspelling (alphabet) sign language recognition, isolated word sign language recognition, and continuous sentences sign language recognition—can be used to classify ArSLR research [9,15].

In this study, a comprehensive review of the current landscape of ArSLR using machine learning and deep learning techniques is conducted. Specifically, the goal is to explore the application of ML and DL in the past decade, the period between 2014 and 2023, to gain deeper insights into ArSLR. To the author’s knowledge, none of the previously reviewed surveys have systematically reviewed the ArSLR studies published in the past decade. Therefore, the main purpose of the current study is to thoroughly understand the progress made in this field, discover valuable information about ArSLR, and shed light on the current state of knowledge. Through an extensive systematic literature review, I have collected and synthesized the most recent results regarding recognizing ArSL using both ML and DL. The analysis involves a collection of techniques, exploring their effectiveness and potential for improving the accuracy of ArSLR. Moreover, current trends and future directions in the area of ArSLR are explored, highlighting important areas of interest and innovation. By understanding the current landscape, the aim is to provide valuable insights into the direction of research and development in this evolving field.

The rest of this paper is organized as follows: in Section 2, the methodology that will help to achieve the goals of this systematic literature review is detailed. In Section 3, the results of the research questions and sub-questions are analyzed, synthesized, and interpreted. In Section 3.4, the most important findings of this study are discussed in an orderly and logical manner, the future perspectives are highlighted, and the limitations of this study are presented. Finally, Section 4 draws the conclusions.

## 2. Research Methodology

**Figure 3:**
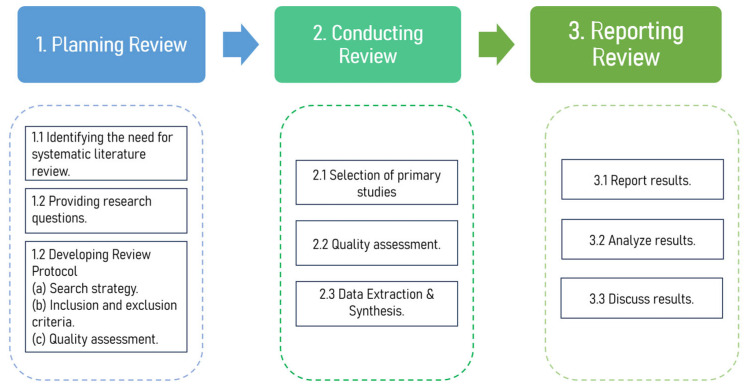
Overview of research methodology.

The systematic literature review gathers and synthesizes the papers published in various scientific databases in an orderly, accurate, and analytical manner about an area of interest. The goal of the systematic approach is to direct the review process on a specific research topic to assess the advancement of research and identify potential new directions. This study adopts the Preferred Reporting Items for Systematic Reviews and Meta-Analyses (PRISMA) guidelines [16]. The research methodology encompasses three key stages: planning, conducting, and reporting the review. Figure 3 provides a visual representation of the methodology applied in this study.

### 2.1. Planning the Systematic Literature Review

The aim of this stage is to determine the need for a systematic literature review and to establish a review protocol. To determine the need for such a systematic review, an exhaustive search of systematic literature views in different scientific databases is performed.

#### 2.1.1. Identification of the Need for a Systematic Literature Review

A search string was developed to find similar systematic literature reviews on ArSLR and to assess whether the planned, systematic review of this study will help close or reduce any gaps in knowledge. Two equivalent search strings were constructed, one for the Web of Science database and one for the Scopus database:

**Web of Science** = TS = ((Arabic sign language) AND recognition AND ((literature review) OR review OR survey OR (systematic literature review)))

**Scopus** = TITLE-ABS-KEY ((Arabic AND sign AND language) AND recognition AND ((literature AND review) OR review OR survey OR (systematic AND literature AND review))).

Any review paper published before 2014 was discarded. After filtering the remaining review papers based on their relevance to the topic of interest, a total of five literature reviews were eligible for inclusion. Three of the review papers [9,15,17] were found in both Web of Science and Scopus. Two papers were found in Scopus [18,19].

In 2014, Mohandes et al. [17] presented an overview of image-based ArSLR studies. Systems and models in the three levels of image-based ArSLR, including alphabet recognition, isolated word recognition, and continuous sign language recognition, are reviewed along with their recognition rate. Mohandes et al. have extended their survey [17] to include not only systems and methods for image-based ArSLR but also sensor-based approaches [15]. This survey also shed light on the main challenges in the field and potential research directions. The datasets used in some of the reviewed papers are briefly discussed [15,17].

A review of the ArSLR background, architecture, approaches, methods, and techniques in the literature published between 2001 and 2017 was conducted by Al-Shamayleh et al. [9]. Vision-based (image-based) and sensor-based approaches were covered. The three levels of ArSLR for both approaches, including alphabet recognition, isolated word recognition, and continuous sign language recognition, were examined with their corresponding recognition rates. The study also identified future research gaps and provided a road map for research in this field. Limited details of the utilized datasets in the reviewed papers were mentioned, such as training and testing data sizes.

Mohamed-Saeed and Abdulbqi [18] presented an overview of sign language and its history and main approaches, including hardware-based and software-based. The classification techniques and algorithms used in sign language research were briefly discussed, along with their accuracy. Tamimi and Hashlmon [19] focused on Arabic sign language datasets and presented an overview of different datasets and how to improve them; however, other areas, such as classification techniques, were not addressed.

In addition to Web of Science and Scopus, Google Scholar was used as a support database to search for systematic literature review papers on ArSLR. Similar keywords were used and yielded four more reviews [2,12,20,21] that are summarized below:

Alselwi and Taşci [10] gave an overview of the vision-based ArSL studies and the challenges the researchers face in this field. They also outlined the future directions of ArSL research. Another study was conducted by Wadhawan and Kumar [2] to systematically review previous studies on the recognition of different sign languages, including Arabic. They provided reviews for the papers published between 2007 and 2017. The papers in each sign language were classified according to six dimensions: acquisition method, signing mode, single/double-handed, static/dynamic signing, techniques used, and recognition rate. Mohammed and Kadhem [20] offered an overview of the ArSL studies published in the period between 2010 and 2019, focusing on input devices utilized in each study, feature extraction techniques, and classification algorithms. They also reviewed some foreign sign language studies. Al Moustafa et al. [21] provided a thorough review of the ArSLR studies in three categories: alphabets, isolated words, and continuous sentence recognition. Additionally, they outlined the public datasets that can be used in the field of ArSLR. Despite mentioning “systematic review” in the paper’s title, they did not follow a systemic approach in their review.

Valuable contributions are made by gathering papers on ArSLR and carrying out a comprehensive literature evaluation. Of the surveys mentioned above, only one adopts a systematic review process [2]; nevertheless, this survey does not analyze or focus on the datasets included in the evaluated studies, and it does cover the studies published after 2017. As a result, it was necessary to provide the community and interested researchers with an analysis of the current status of ArSLR. The purpose of this research, therefore, is to close or at least lessen that gap. All the review papers are listed and summarized in Table 1 in chronological order from the oldest to the newest.

#### 2.1.2. Providing Research Questions

This section discusses the fundamental questions to be investigated in the study. The research questions are divided into three main groups:

- Questions about the used datasets
- Questions about the used algorithms and methods
- Questions about the research limitations and future directions.

Table 2, Table 3 and Table 4 display the questions along with their respective purposes.

#### 2.1.3. Developing Review Protocol

Once the research questions and sub-questions were specified, the scope of the research was defined using the PICO method proposed by Petticrew and Roberts [22]:

P: Population. Deaf individuals who use Arabic sign language to communicate.

I: Intervention. Recognition of Arabic sign language.

C: Comparison. The machine learning and deep learning algorithms adopted for Arabic sign language recognition and the datasets developed for Arabic sign language.

O: Outcomes. Performance reported in the selected studies.

##### Search Strategy

To establish a systematic literature review, it is crucial to specify the search terms and to identify the scientific databases where the search will be conducted. In 2019, a study was carried out to evaluate the search quality of PubMed, Google Scholar, and 26 other academic search databases [23]; the results revealed that Google Scholar is not suitable as a primary search resource. Therefore, the most widely used scientific databases in the field of research were selected for this systematic review, namely Web of Science, Scopus, and IEEE Xplore Digital Library. Each database was last consulted on 2 January 2024.

A search string is an essential component of a systematic literature review for study selection, as it restricts the scope and coverage of the search. A set of search strings was created with the Boolean operator combining suitable synonyms and alternate terms: AND restricts and limits the results, and OR expands and extends the search [24]. Moreover, double quotes have been used to search for exact phrases. At this initial stage, the search was limited to the title, abstract, and keywords of the papers. Each database has different reserved words to indicate these three elements, such as TS in Web of Science, TITLE-ABS-KEY in Scopus, and All Metadata in IEEE Xplore. The specific search strings used in each scientific database are listed below:

Web of Science:

TS = (Arabic sign language) AND recognition

Scopus:

TITLE-ABS-KEY (“Arabic sign language” AND recognition)

IEEE Xplore:

((“All Metadata”: “Arabic sign language”) AND (“All Metadata”: recognition))

##### Inclusion and Exclusion Criteria

The selection process of the studies has a significant impact on the systematic review’s findings. As a result, all studies that were found using the search strings were assessed to see if they should be included in this review. The review excluded all studies that did not fulfill all inclusion criteria. A study was excluded if it satisfied at least one of the exclusion criteria. The inclusion criteria used in this systematic review are:

**IN1**: Papers must be peer-reviewed articles and published in journals.

**IN2**: Papers that are written in English.

**IN3:** Papers are published between January 2014 and December 2023.

**IN4**: Papers must be primary studies.

**IN5**: Papers that use Arabic sign language as a language for recognition.

**IN6**: Papers that use machine learning and/or deep learning methods as a solution to the problem of ArSLR.

**IN7**: Papers that include details about the achieved results.

**IN8**: Papers that mention the datasets used in their experiment.

We used several criteria to discard papers from the candidate set. The exclusion criteria (EC) are as follows:

**EC1**: Papers that are conference proceedings, data papers, books, notes, theses, letters, or patents.

**EC2**: Papers that are not written in English.

**EC3:** Papers that are published before January 2014 or after December 2023.

**EC4**: Papers that are secondary studies (i.e., review, survey).

**EC5**: Papers that do not use Arabic sign language as a language for recognition.

**EC6**: Papers that do not use machine learning or deep learning methods as a solution to the problem of ArSLR.

**EC7**: Papers that do not include details about the achieved results.

**EC8**: Papers that do not mention the datasets used in their experiment.

**EC9:** Papers with multiple versions are not included; only one version will be included.

**EC10**: Duplicate papers found in more than one database (e.g., in both Scopus and Web of Science) are not included; only one will be included.

**EC11:** The full text is not accessible.

##### Quality Assessment

It is deemed essential to assess the quality of the primary studies in addition to inclusion/exclusion criteria to offer more thorough criteria for inclusion and exclusion, to direct the interpretation of results, and to direct suggestions for further research [16]. In-depth assessments of quality are typically conducted using quality instruments, which are checklists of variables that must be considered for each primary study. Numerical evaluations of quality can be achieved if a checklist’s quality items are represented by numerical scales. Typically, checklists are created by considering variables that might affect study findings. A quality checklist aims to contribute to the selection of studies through a set of questions that must be answered to guide the research. In the current study, a scoring system based on the answers to six questions was applied to each study. These questions are:

QA1: Is the experiment on ArSLR clearly explained?

QA2: Are the machine learning/deep learning algorithms used in the paper clearly described?

QA3: Is the dataset and number of training and testing data identified clearly?

QA4: Are the performance metrics defined?

QA5: Are the performance results clearly shown and discussed?

QA6: Are the study’s drawbacks or limitations mentioned clearly?

Each study obtained a score of three for each question: 0, 0.5, and 1, denoting no, partially, and yes, respectively. A study was finally taken into consideration if it received a score of 4 or more out of 6 on the previous questions.

### 2.2. Conducting the Systematic Literature Review

**Figure 4:**
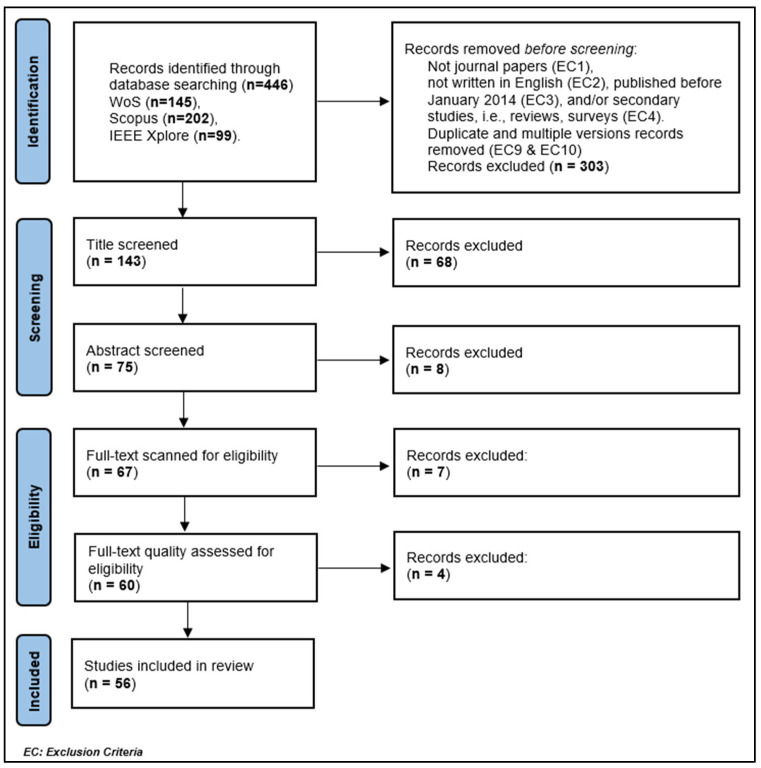
Steps of paper selection.

The search results and evaluation of the papers chosen for inclusion are discussed here. Figure 4 summarizes the number of eligible papers and shows how many were eliminated in each step.

#### 2.2.1. Selection of Primary Studies

The search for peer-reviewed papers was conducted in three large databases: Web of Science, Scopus, and IEEE Xplore. The Web of Science, Scopus, and IEEE Xplore Digital Library databases are estimated to be suitable because there are duplicate papers in the search results, i.e., the same work appears in different databases at the same time. This indicates the very high coverage of these scientific databases. These databases were searched using predetermined search strings, as specified in the review protocol above. There were **145** papers found in Web of Science and **202** and **99** in Scopus and IEEE Xplore, respectively.

Of the **446** papers in the initial search, the exclusion criteria were applied automatically to select eligible papers as follows: **303** papers were discarded because they were conference proceedings, data papers, books, notes, theses, letters, or patents (EC1), not written in English (EC2), published before January 2014 (EC3), and/or secondary studies, i.e., reviews, surveys (EC4). At this stage, the number of remaining papers was 62, 74, and 7 in Web of Science, Scopus, and IEEE Xplore, respectively.

##### Title-Level Screening Stage

Exclusion criteria EC9 and EC10 were applied to remove duplicate papers and multiple versions. The titles were also screened to discard the papers that meet EC1, EC4, and EC5. In this stage, we excluded 68 research papers; thus, the number of papers was lowered to **75** eligible papers. Examples of research papers that were excluded in this stage are presented in Table 5.

**Table 5:** The reasoning behind the exclusion of some papers in the title-level screening stage.

Ref.	Paper Title	Reason for Exclusion
[25]	Indian sign language recognition system using network deconvolution and spatial transformer network	EC5—Indian sign language recognition not ArSLR
[26]	SignsWorld Atlas; a benchmark Arabic Sign Language database	EC1—Data paper
[15]	Image-Based and Sensor-Based Approaches to Arabic Sign Language Recognition	EC4—Review paper

##### Abstract-Level Screening Stage

In this stage, the abstracts for the **75** papers were screened to shorten paper reading time. The paper is excluded if the abstract meets at least one of the exclusion criteria: **EC5**: Papers that do not use Arabic sign language as a language for recognition and/or **EC6**: Papers that do not use machine learning or deep learning methods as a solution to the problem of ArSLR.

As a result of this screening, **eight** research papers were excluded, and **67** were included in the full-text article scanning. Examples of research papers that were excluded in this stage are included in Table 6.

**Table 6:** The reasoning behind the exclusion of some papers in the abstract-level screening stage.

Ref.	Paper Title	Reason for Exclusion
[27]	A machine translation system from Arabic sign language to Arabic	EC6—Paper does not use machine learning or deep learning methods as a solution to the problem of ArSLR
[28]	Isolated Video-Based Arabic Sign Language Recognition Using Convolutional and Recursive Neural Networks	EC5—Paper does not use ArSL as a language for recognition. Moroccan sign language recognition is used. Although Moroccan is an Arabic delicate, Moroccan sign language is different from the unified Arabic sign language.

##### Full-Text Scanning Stage

In this stage, the full texts of **67** papers are scanned. The paper is excluded if it satisfies one of the following exclusion criteria,

**EC5**: Papers that do not use Arabic sign language as a language for recognition,

**EC6**: Papers that do not use machine learning or deep learning methods as a solution to the problem of ArSLR,

**EC7**: Papers that do not include details about the achieved results,

**EC8**: Papers that do not mention the datasets used in their experiment,

**EC9:** Papers with multiple versions are not included; only one version will be included, or

**EC11:** The full text is not accessible.

A total of **Seven** research papers were excluded in this stage, making the number of studies for the next stage **60**. Examples of the excluded research papers and the reasoning for their exclusion are illustrated in Table 7.

**Table 7:** The reasoning behind the exclusion of some papers in the full-text scanning stage.

Ref.	Paper Title	Reason for Exclusion
[29]	Supervised learning classifiers for Arabic gestures recognition using Kinect V2	EC5—Paper does not use ArSL as a language for recognition. Egyption sign language recognition is used. Although Egyption is an Arabic delicate, Egyption sign language is different from the unified Arabic sign language (ArSL).
[30]	Arabic dynamic gesture recognition using classifier fusion	
[31]	Intelligent real-time Arabic sign language classification using attention-based inception and BiLSTM	EC5—Paper does not use ArSL as a language for recognition. Saudi sign language recognition is used. Saudi is an Arabic delicate, however; Saudi sign language is different from the unified Arabic sign language (ArSL).
[32]	Arabic sign language intelligent translator	EC9—Papers with multiple versions are not included; only one version will be included. The same methodology and results discussed in this paper are presented in another paper published in 2019 and written by the same authors [33].
[34]	Mobile camera-based sign language to speech by using zernike moments and neural network	EC11—The full text is not accessible.

#### 2.2.2. Quality Assessment Results

This section presents the quality assessment results used to select relevant papers based on the quality criteria defined in Section 2.1.3. Failure to provide information to meet these criteria leads to a lower paper evaluation score. Papers with a score of 3.5 or lower out of 6 are removed from the pool of candidates. As a result, only high-quality papers are taken into consideration. Table 8 shows some example research papers from this stage and elaborates on how and why they were included or excluded.

**Table 8:** The reasoning behind the exclusion or inclusion of some papers in the full-text article screening stage.

Reference	Score						Total Score	Included
	QA1	QA2	QA3	QA4	QA5	QA6		
Podder et al. [35]	1	1	1	1	1	1	6	Yes
Aldhahri et al. [36]	1	0.5	1	0	0.5	0	3	No

**Figure 5:**
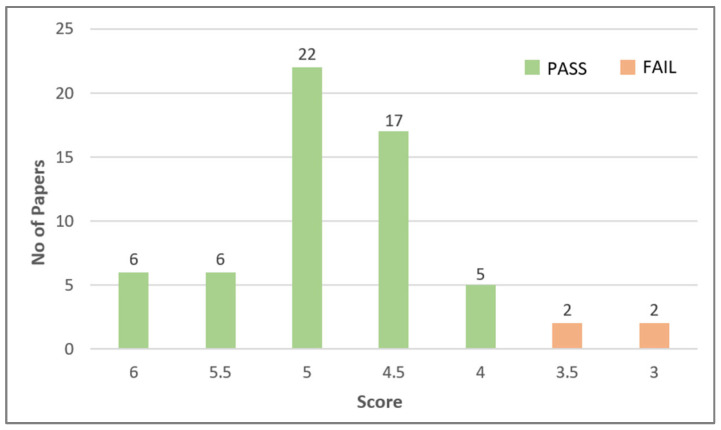
Number of papers passed and failed the quality assessment.

Out of 60 papers, 56 of them met the quality assessment criteria. The number of papers that failed and passed these criteria is illustrated in Figure 5.

One of the reasons why some papers fail to pass the quality assessment phase is that some papers did not explain their ArSLR methodology clearly and thoroughly. The other reasons are an insufficient description of the utilized ML or DL algorithms and/or a lack of information related to the used datasets, such as the number of training and testing datasets. Missing identification of performance metrics and poor analysis and discussion of the results also contributed to removing some papers from the pool of candidates. Many of the studies included a detailed explanation of their methodology; however, some neglected to address the limitations and drawbacks of the techniques, which also affected the assessment.

**Figure 6:**
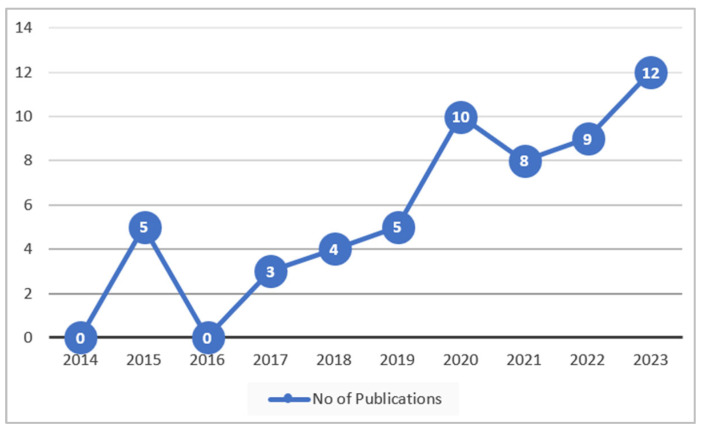
Number of eligible papers published per year.

As seen in Figure 6, an increase has been observed in recent years, from 2017 to 2023, in the research of ArSLR, which itself exhibits the significance of this investigation.

**Figure 7:**
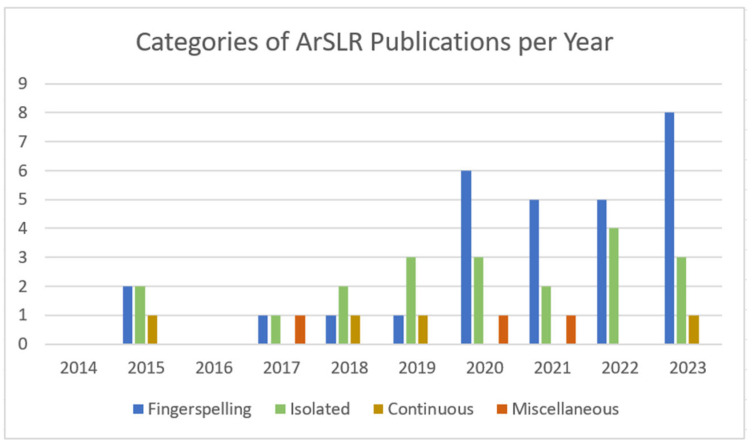
Number of publications for each category of ArSLR for the years between 2014 and 2023.

Figure 7 reveals the number of publications for each category of ArSLR for the years between 2014 and 2023. It can be seen that, over the years, fingerspelling recognition has attracted more researchers, followed by isolated recognition and then continuous recognition. It is worth noting that a few of the selected papers have worked on more than one category of ArSLR, and they belong to the category of miscellaneous recognition [37,38,39].

#### 2.2.3. Data Extraction and Synthesis

After reading the full text, the author aims to extract the answers to the defined research questions from each study of the 56 papers included in this systematic review. The extracted data were synthesized to provide a comprehensive summary of the results. The author used a test-retest strategy and reassessed a random sample of the primary studies that were identified following the first screening in order to verify the consistency of their inclusion/exclusion judgments.

### 2.3. Reporting the Systematic Literature Review

Addressing the research questions and sub-questions listed in Table 2, Table 3 and Table 4 is the main aim of this stage. The following section presents the literature review, answering questions RQ1 through RQ3 and making a summary and synthesis of the data gathered from the results of the selected studies.

## 3. Research Results

In this section, the answer to each research question is provided by summarizing and discussing the results of the selected papers.

### 3.1. Datasets of Arabic Sign Language

To answer the first research question, RQ1: What are the characteristics of the datasets used in the selected papers? Twelve research sub-questions have been answered. These questions address different aspects and characteristics of the datasets utilized in the selected papers.

Large volumes of data are necessary for ML/DL models to carry out certain tasks accurately. One of the obstacles to the advancement of Arabic sign language recognition research and development is the availability of datasets [27]. Despite the huge number of sign language videos on the internet, recognition systems cannot benefit from these unannotated videos.

Sign language datasets fall into three primary categories: fingerspelling, isolated signs, and continuous signs. Fingerspelling datasets focus on the shape, orientation, and direction of the finger. Sign language for numbers and alphabets belongs to this category, where the signs are primarily static and performed with just fingers. Datasets for isolated words can be either static (images) or dynamic (videos). Continuous sign language datasets consist of videos of signers performing more than one sign word continuously. Combinations of the other collections make up the fourth category of datasets, known as miscellaneous datasets.

Table 9, Table 10, Table 11 and Table 12 summarize the main characteristics of the datasets in the reviewed papers according to the category they represent. ArSL datasets for Arabic alphabets and/or numbers are summarized in Table 9. Table 10 and Table 11 describe ArSL datasets for words and sentences, whereas Table 12 summarizes ArSL miscellaneous datasets. Rows shaded in gray indicate methods that utilize wearable sensors.

**Table 9:** Fingerspelling ArSL datasets. “PR” means Private dataset, “AUR” means Available Upon Request dataset, and “PA” means Publicly Available dataset.

Year	Dataset	Created/Used by			Availability	# Signers (Subjects)	Samples	# Signs (Classes)	MS/NMS	Data Acquisition Device	Acquisition Modalities	Images/Videos or Others?	Collection				Signing Mode			Static/Dynamic	Data Collection Location/Country	Comments
		Ref.	Year	With Other Datasets?									Alphabets	Numbers	Words	Sentences	Isolated	Continuous	Fingerspelling			
2001	Al-jarrah dataset [40]	[41]	2023	No	AUR	60	1800	30	MS	Camera	RGB	128 × 128 pixels grey scale images	✓	✗	✗	✗	✗	✗	✓	S	Jordan	
2015	ArASLRDB [42]	[42]	2015	No	PR	-	357	38	MS	Camera	RGB	640 × 480 pixels saved in jpg file format	✓	✓	✗	✗	✗	✗	✓	S	Egypt	29 letters, 9 numbers
2015	The Suez Canal University ArSL dataset [43]	[43]	2015	No	AUR	-	210	30	MS	Camera	RGB	200 × 200 Gray-scale images	✓	✗	✗	✗	✗	✗	✓	S	Egypt	7 images for each letter gesture
		[44]	2020	No																		
2016	ArSL [45]	[46]	2023	Yes	AUR	-	350	14	MS	Camera	RGB	64 × 64 grey scale images	✓	✗	✗	✗	✗	✗	✓	S	Egypt	
		[47]	2023	Yes																		
2017	[48]	[48]	2017	No	PR	20	1398	28	MS	Microsoft Kinect V2 and Leap Motion Controller (LMC) sensors (manufactured by Leap Motion, Inc., a company based in San Francisco, CA, USA. Leap Motion, Inc. has since merged with Ultraleap, a UK-based company)	Depth, skeleton models	All upper joints (hand’s skeleton data).	✓	✗	✗	✗	✗	✗	✓	S	Saudi Arabia	
2018	[49]	[49]	2018	No	PR	30	900	30	MS	Smart phone cameras	RGB	RGB	✓	✗	✗	✗	✗	✗	✓	S	Saudi Arabia	
2019	ArSL2018 [50,51]	[52]	2019	No	PA	40	54,049	32	MS	smart camera (iPhone 6S manufactured by Apple Inc., headquartered in Cupertino, CA, USA)	RGB	64 × 64 pixels gray scale images	✓	✗	✗	✗	✗	✗	✓	S	Saudi Arabia	
		[53]	2020	No																		
		[54]	2020	No																		
		[38]	2020	YES																		
		[55]	2020	No																		
		[56]	2021	YES																		
		[57]	2021	No																		
		[58]	2022	No																		
		[59]	2022	No																		
		[60]	2022	No																		
		[61]	2022	No																		
		[47]	2023	Yes																		
		[62]	2023	Yes																		
		[63]	2023	Yes																		
		[46]	2023	Yes																		
		[64]	2023	No																		
		[65]	2023	No																		
		[66]	2023	No																		
2019	Ibn Zohr University dataset [67]	[63]	2023	Yes	AUR	-	5839	28	MS	professional Canon^®^ camera (manufactured by Canon Inc., headquartered in Ōta City, Tokyo, Japan).	RGB	256 × 256 pixels in RGB colored images	✓	✗	✗	✗	✗	✗	✓	S	Morocco	
2020	[68]	[68]	2020	No	PR	10	300	28	MS	RGB digital camera	RGB	128 × 128 RGB images	✓	✗	✗	✗	✗	✗	✓	S	Egypt	
2020	[69]	[69]	2020	No	PR	-	3875	31	MS	Camera	RGB	128 × 128 RGB images	✓	✗	✗	✗	✗	✗	✓	S	Saudi Arabia	125 images for each letter
2021	[70]	[70]	2021	No	PR	7	4900	28	MS	Six 3-D IMU sensors (manufactured by SparkFun Electronics, headquartered in Boulder, CO, USA).	Raw data	Gyroscopes and accelerometers data	✓	✗	✗	✗	✗	✗	✓	S	Palestine	
2021	[71]	[71]	2021	Yes	PR	10	22,000	44	MS	Camera	RGB	RGB image	✓	✓	✗	✗	✗	✗	✓	S	Iraq	32 letters, 11 numbers (0:10), and 1 sign for none
2021	[72]	[72]	2021	Yes	PR	10	2800	28	MS	Webcam and smart mobile cameras	RGB	RGB images	✓	✗	✗	✗	✗	✗	✓	S	Egypt	Light background
2021	[72]	[72]	2021	Yes	PR	10	2800	28	MS	Webcam and smart mobile cameras	RGB	RGB images	✓	✗	✗	✗	✗	✗	✓	S	Egypt	Dark background
2021	[72]	[72]	2021	Yes	PR	10	1400	28	MS	Webcam and smart mobile cameras	RGB	RGB images	✓	✗	✗	✗	✗	✗	✓	S	Egypt	Images made with a right hand and wearing gloves, white background
2021	[72]	[72]	2021	Yes	PR	10	1400	28	MS	Webcam and smart mobile cameras	RGB	RGB images	✓	✗	✗	✗	✗	✗	✓	S	Egypt	Images made with a bare hand “two hands” and wearing glove with different background
2021	[73]	[73]	2021	No	PR	-	15,360	30	MS	Mobile cameras	RGB	720 × 960 × 3 RGB images	✓	✗	✗	✗	✗	✗	✓	S	Saudi Arabia	
2022	[74]	[74]	2022	No	PR	20+	5400	30	MS	Smart phone camera	RGB	RGB	✓	✗	✗	✗	✗	✗	✓	S	Saudi Arabia	
2023	[63]	[63]	2023	Yes	PR	30	840	28	MS	Camera	RGB	64 × 64 pixels gray scale images	✓	✗	✗	✗	✗	✗	✓	S	Iraq	50% left-handed, 50% right-handed

**Table 10:** ArSL Datasets for isolated words. “PR” means Private dataset, “AUR” means Available Upon Request dataset, and “PA” means Publicly Available dataset.

Year	Dataset	Created/Used by			Availability	# Signers (Subjects)	Samples	# Signs (Classes)	MS/NMS	Data Acquisition Device	Acquisition Modalities	Images/Videos or Others?	Collection				Signing Mode			Static/Dynamic	Data Collection Location/Country	Comments
		Ref.	Year	With Other Datasets?									Alphabets	Numbers	Words	Sentences	Isolated	Continuous	Fingerspelling			
2007	Shanableh dataset [75]	[76]	2019	Yes	AUR	3	3450	23	MS	Video camera	RGB	RGB video recorded at 25 fps with 320 × 240 resolution.	✗	✗	✓	✗	✓	✗	✗	D	United Arab Emirates (UAE)	Words chosen from the greeting section
		[77]	2020	No																		
		[78]	2022	Yes																		
2015	EMCC database (EMCCDB) [79]	[79]	2015	No	PR	3	1288	40	MS	Camera	RGB	30 frames per second for video frames and the size of the frames is 640 × 480 pixels.	✗	✗	✓	✗	✓	✗	✗	D	Egypt	
2015	Dataset for hands recognition [80]	[80]	2015	YES	PR	2	80	20	MS	LMC	Skelton joint points	Sequences of frames	✗	✗	✓	✗	✓	✗	✗	D	Egypt	
2015	Dataset for facial expressions recognition [80]	[80]	2015	YES	PR	2	50	5	NMS	Digital camera	RGB	70 × 70 RGB image	✗	✗	✓	✗	✓	✗	✗	S	Egypt	5 basic facial expressions: happy, sad, normal, surprised, and looking-up.
2015	Dataset for body movement recognition [80]	[80]	2015	YES	PR	2	NM	21	NMS	Digital camera	RGB	100 × 100 RGB image	✗	✗	✓	✗	✓	✗	✗	S	Egypt	16 different hand positions, and 5 shoulder movements.
2017	[81]	[81]	2017	No	PR	10	143	5	MS	Microsoft Kinect V2 and LMC sensors	Depth, skelton models	All features of upper joints including the hand’s skeleton	✗	✗	✓	✗	✓	✗	✗	S	Saudi Arabia	Words [Cruel, Giant, Plate, Tower, Objection]
2018	[82]	[82]	2018	YES	PR	21	7500	150	MS & NMS	Microsoft Kinect V2	RGB video, depth video, 3D skeleton sequence, sequence of hand states, sequence of face features.	Joints of the upper body part, which are 16 joints are used.	✗	✗	✓	✗	✓	✗	✗	D	Egypt	
2018	[5]	[5]	2018	No	PR		450	30	MS	Camera	RGB	Colored videos at a rate of 30 fps	✗	✗	✓	✗	✓	✗	✗	D	Egypt	30 daily commonly used words at school
2019	[83]	[83]	2019	No	PR	2	2000	100	MS	Front and side LMCs.	RGB	Sequence of frames.	✗	✗	✓	✗	✓	✗	✗	D	Saudi Arabia	
2019	[76]	[76]	2019	YES	PR	3	7500	50	MS	Microsoft Kinect V2	RGB, depth, and skeleton joints	The color images are saved into MP4 video, depth frames and skeleton joints are saved into binary files.	✗	✗	✓	✗	✓	✗	✗	D	Saudi Arabia	
2019	ArSL Isolated Gesture Dataset [33]	[33]	2019	No	AUR	3	1500	100	MS	Camera	RGB	Each video has a frame speed of 30 frames per second	✓	✓	✓	✗	✓	✗	✗	D	Egypt	30 Alphabets, 10 numbers, 10 Prepositions, pronouns and question words, 10 Arabic life expressions, and 40 Common nouns and verbs.
2020	[84]	[84]	2020	NO	PR	5	440	44	MS	LMC	Skelton joint points	Sequences of frames	✗	✗	✓	✗	✓	✗	✗	D	Syria	29 signs are one hand gestures, and 15 signs are two hand gestures
2020	[85]	[85]	2020	No	PR	10	222	6	MS	Kinect sensor	Depth	Depth video frames	✗	✗	✓	✗	✓	✗	✗	D	Saudi Arabia	Arabic Words [Common-, Protein, Stick, Disaster, Celebrity, Bacteria]
2020	[38]	[38]	2020	Yes	PR	-	288	10	MS	Mobile camera	RGB	RGB images	✗	✗	✓	✗	✓	✗	✗	S	Egypt	
2021	KArSL-33 [86,87]	[78]	2022	Yes	PA	3	4950	33	MS & NMS	Kinect V2	Depth, Skeleton joint points, RGB	RGB video format	✗	✗	✓	✗	✓	✗	✗	D	Saudi Arabia	
2021	mArSL [88,89]	[88]	2021	No	AUR	4	6748	50	MS & NMS	Kinect V2	RGB, depth, joint points, face, and faceHD.	224 × 224 RGB video frames	✗	✗	✓	✗	✓	✗	✗	D	Saudi Arabia	
		[35]	2023	No																		
2021	KArSL-100 [86,87]	[78]	2022	Yes	PA	3	15,000	100	MS & NMS	Kinect V2	RGB, depth, skeleton joint points	RGB video format	✓	✓	✓	✗	✓	✗	✗	S & D	Saudi Arabia	30-digit signs, 39 letter signs, and 31-word signs.
	KArSL-190 [86,87]	[78,90]	2022	Yes		3	28,500	190					✓	✓	✓	✗	✓	✗	✗	S & D	Saudi Arabia	30-digit signs, 39 letter signs, and 121-word signs.
	KArSL-502 [86,87]	[90]	2022	Yes		3	75,300	502					✓	✓	✓	✗	✓	✗	✗	S & D	Saudi Arabia	30 digit and signs, 39 letter signs, and 433 sign words.
2022	[91]	[91]	2022	No	PR	-	1100	11	MS	-	RGB	RGB video format	✗	✗	✓	✗	✓	✗	✗	D	Saudi Arabia	11 Words: [Friend, Neighbor, Guest, Gift, Enemy, To Smel, To Help, Thank you, Come in, Shame, House]
2022	[92]	[92]	2022	No	PR	55	7350	21	MS	Kinect camera V2	RGB and Depth video	RGB video format	✗	✗	✓	✗	✓	✗	✗	D	Iraq	Words: [Nothing, Cheek, Friend, Plate, Marriage, Moon, Break, Broom, You, Mirror, Table, Truth, Watch, Arch, Successful, Short, Smoking, I, Push, stingy, and Long]
2023	[93]	[93]	2023	No	PR	1	500	5	MS	-	-	-	✗	✗	✓	✗	✓	✗	✗	S	Saudi Arabia	
2023	[94]	[94]	2023	No	PR	72	8467	20	MS	Mobile camera	RGB	Videos	✗	✗	✓	✗	✓	✗	✗	D	Egypt	

**Table 11:** ArSL Datasets for continuous sentences. “PR” means Private dataset, “AUR” means Available Upon Request dataset, and “PA” means Publicly Available dataset.

Year	Dataset	Created/Used by			Availability	# Signers (Subjects)	Samples	# Signs (Classes)	MS/NMS	Data Acquisition Device	Acquisition Modalities	Images/Videos or Others?	Collection				Signing Mode			Static/Dynamic	Data Collection Location/Country	Comments
		Ref.	Year	With Other Datasets?									Alphabets	Numbers	Words	Sentences	Isolated	Continuous	Fingerspelling			
2015	Tubaiz, Shanableh, & Assaleh dataset [95]	[95]	2015	No	AUR	1	400 sentences 800 words	40	MS	Two DG5-VHand data gloves and (a video camera in training phase)	Raw sensor data (feature vectors) for hand movement and hand orientation	Sensor readings	✗	✗	✗	✓	✗	✓	✗	D	UAE	An 80-word lexicon was used to form 40 sentences.
		[96]	2019	Yes																		
2018	[97]	[97]	2018	No	PR	7	1260 samples	42	MS	Microsoft Kinect V2	RGB, depth, skeleton joints	For each sign, a sequence of skeleton data consisted of 20 joint positions per frame is recorded (formed from x, y, and depth coordinates)	✗	✗	✓	✓	✗	✓	✗	D	Egypt	Sentences containing medical terms performed by 7 signers (5 training, 2 testing). (20 samples from different 5 signers for each sign as a training set and 10 samples from different 2 signers as a testing set)
2019	Self-acquired sensor-based dataset 2 [96]	[96]	2019	YES	AUR	2	400 sentences 800 words.	40	MS	Two Polhemus G4 motion trackers	Raw data	Motion tracker readings	✗	✗	✗	✓	✗	✓	✗	D	Egypt	
2019	Self-acquired vision-based dataset 3 [96]	[96]	2019	Yes	AUR	1	400 sentences 800 words.	40	MS	Camera	RGB	Videos with frame rate set to 25 Hz with a spatial resolution of 720 × 528	✗	✗	✗	✓	✗	✓	✗	D	Egypt	-
		[98]	2023	No																		

**Table 12:** ArSL Miscellaneous Datasets. “PR” means Private dataset, “AUR” means Available Upon Request dataset, and “PA” means Publicly Available dataset.

Year	Dataset	Created or Used by			Availability	# Signers (Subjects)	Samples	# Signs (Classes)	MS/NMS	Data Acquisition Device	Acquisition Modalities	Images/Videos or Others?	Collection				Signing Mode			Static/Dynamic	Data Collection Location/Country	Comments
		Ref.	Year	With Other Datasets?									Alphabets	Numbers	Words	Sentences	Isolated	Continuous	Fingerspelling			
2017	[37]	[37]	2017	No	PR	3	26,060 samples	79	MS	LMC	Hands skeleton joint points	Sequences of frames	✓	✓	✓	✓	✓	✓	✓	S & D	Egypt	Alphabets: 28, Numbers: 11 (0–10), common dentist words: 8, common verbs and nouns-single hand: 20, common verbs and nouns-two hands: 10
2021	[39]	[39]	2021	No	PR	40	16,000 videos	80	MS & NMS	Kinect camera V2	RGB, depth, skeleton data	1280 × 920 × 3 RGB frames	✓	✓	✓	✗	✓	✗	✓	S & D	Saudi Arabia	

#### 3.1.1. RQ1.1: How Many Datasets Are Used in Each of the Selected Papers?

**Figure 8:**
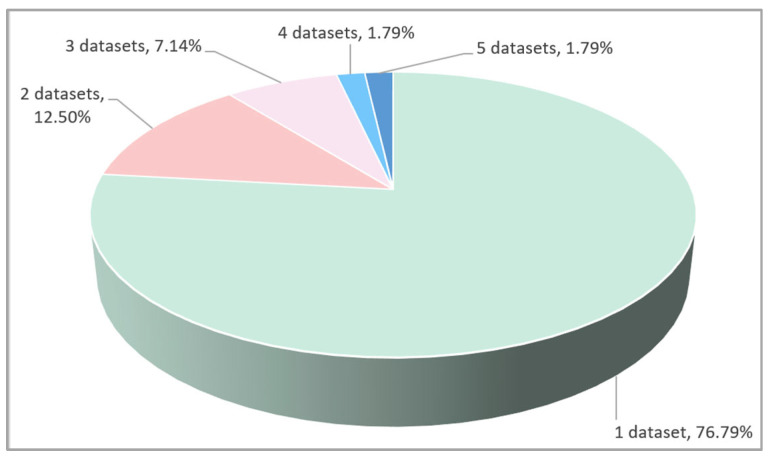
Percentage of the reviewed studies based on the number of utilized datasets.

According to the results in Figure 8, we can see that a high percentage of the reviewed papers, 76.79%, representing 43 papers, use one dataset in their experiments. Seven papers, with a percentage of 12.5%, rely on two datasets to conduct their experiments. Three and four datasets are utilized in four and one studies, with a percentage of 7.14% and 1.79%, respectively. Only one study uses five datasets with a percentage of 1.79%.

One of the following could be the rationale for using multiple datasets:

1. Incorporate different collections of sign language, for example, a dataset for letters and a dataset for words.
2. Collect sign language datasets pertaining to many domains, such as health-related words and greeting words.
3. Collect datasets representing different modalities, for example, one for JPG and the other for depth and skeleton joints and/or different data acquisition devices.
4. Enhance the study by training and testing the model using more than one sign language dataset and/or comparing the results.
5. Conduct user-independent sign language recognition, where the proposed mode is tested using signs represented by different signers from those in the training set.

Table 13 summarizes the reasons behind the use of more than one dataset in the reviewed studies.

#### 3.1.2. RQ1.2: What Is the Availability Status of the Datasets in the Reviewed Papers?

**Figure 9:**
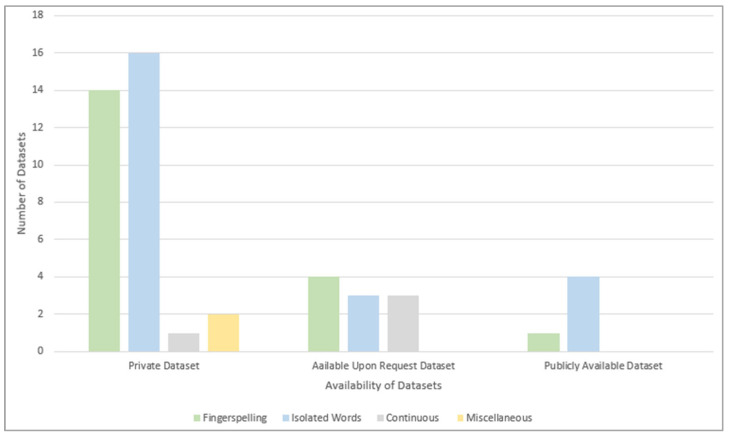
Availability status of the reviewed datasets in each dataset category.

We can divide the availability of the datasets in the reviewed papers into three main groups: publicly available, available upon request, and self-acquired private datasets. Figure 9 shows that self-acquired private datasets constitute the bulk of the reviewed datasets, followed by datasets that can be obtained upon request and those that are publicly available. Due to the lack of available upon request and publicly available datasets for the fingerspelling, isolated, or miscellaneous datasets, the researchers who worked on them had to build their own private ArSL datasets. On the contrary, around three-quarters of the continuous datasets are available upon request, while the remaining dataset was built privately.

Public and available upon-request datasets are discussed according to the dataset category. Examples of each of the datasets are also presented where available.

##### Category 1: Fingerspelling Datasets

- Arabic Alphabets Sign Language (ArSL2018)

**Figure 10:**
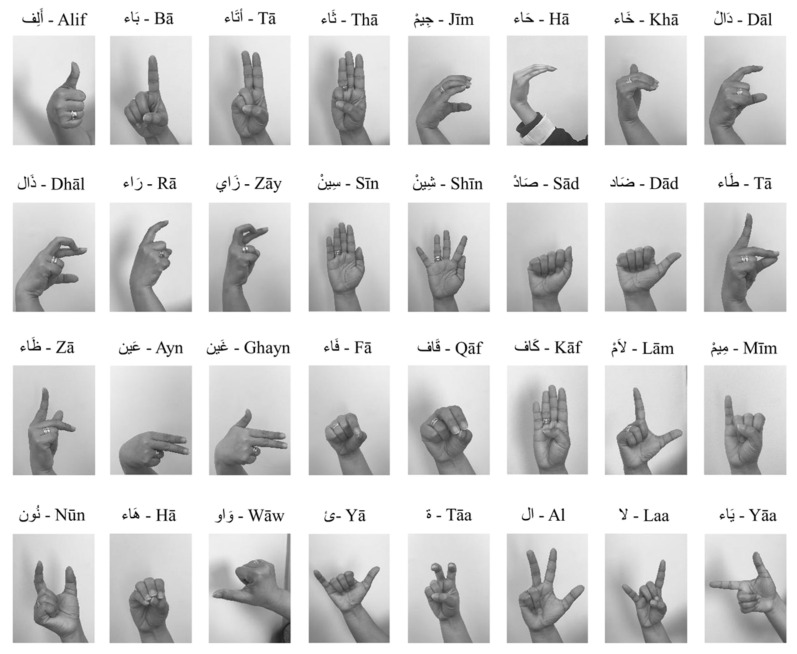
The ArSL2018 dataset, illustration of the ArSL for Arabic alphabets [48,49].

This publicly available dataset [50,51] consists of 54,049 images of ArSL alphabets for 32 Arabic signs, performed by 40 signers of various age groups. Each alphabet (class) has a different number of images. The RGB format was used to capture the images, which had varying backgrounds, lighting, angles, timings, and sizes. Image preprocessing was carried out to prepare for classification and recognition. The gathered images were adjusted to a fixed dimension of 64 × 64 and converted into grayscale images, meaning that individual pixels may have values ranging from 0 to 255. Figure 10 shows a sample of the ArSL2018 dataset.

- Al-Jarrah Dataset

This dataset [40] is available upon request and contains gray-scale images. For every gesture, 60 signers executed a total of 60 samples. Of the 60 samples available for each motion, 40 were used for training, and the remaining 20 were used for testing.

**Figure 11:**
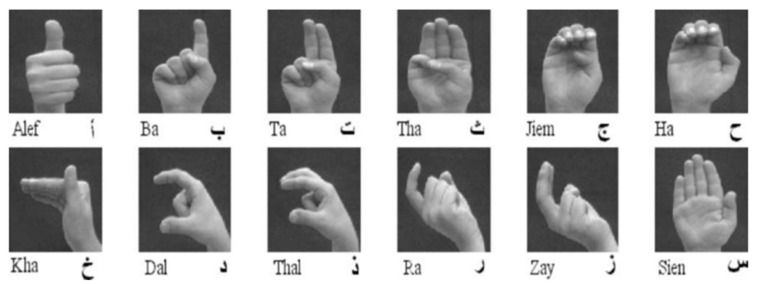
Samples of ArSL alphabets [39].

For training purposes, 40 of the 60 samples for each gesture were used, while the remaining 20 samples were used for testing. The samples were captured in various orientations and at varying distances from the camera. Samples of the dataset are exhibited in Figure 11.

- The Suez Canal University ArSL Dataset

This dataset [43] consists of 210 grayscale ArSL images with size 200 × 200. These images represent 30 letters, with seven images for each letter. Different rotations, lighting, quality settings, and picture bias were all taken into consideration when capturing the images. All images are centered and cropped to 200 × 200. These images were gathered from a range of signers with varying hand sizes. This dataset is available upon request from the researchers.

- ArSL Dataset

**Figure 12:**
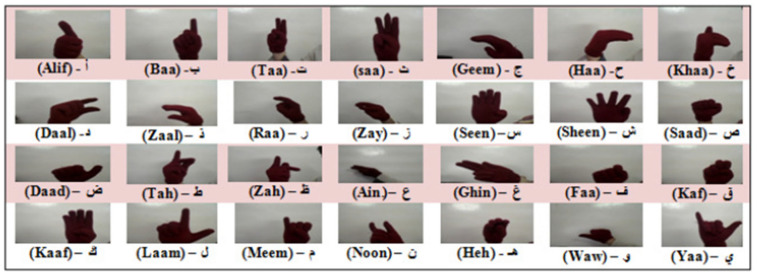
Samples of the ArSL Alphabet in the ArSL dataset [43].

The 700 color images in this dataset [45], which is available upon request, show the motions of 28 Arabic letters, with 25 images per letter. Various settings and lighting conditions were used to take the images included in the dataset. Different signers wearing dark-colored gloves and with varying hand sizes executed the actions, as shown in Figure 12.

- Ibn Zohr University Dataset

This dataset [67], which is available upon request, is made up of 5771 images for 28 Arabic letters. The color size of these images is 256 × 256 pixels.

##### Category 2: Isolated Datasets

- Shanableh Dataset

This dataset [75], which is available upon request, includes ArSL videos of 23 Arabic gesture words/sentences that are signed by three distinct signers. The 23 gestures were recorded in 3450 videos, with each gesture being repeated 150 times, thanks to the 50 repetitions of each gesture made by each of the three signers. Using no restrictions on background or clothing, the signer was recorded while executing the signs using an analog camcorder. The final videos from each session were edited into short sequences that each individually represented a gesture after being converted to digital video.

- KArSL-33

There are 33 dynamic sign words in the publicly available KArSL-33 dataset [86,87]. Each sign was executed by three experienced signers. By performing each sign 50 times by each signer, a total of 4950 samples (33 × 3 × 50) were generated. There are three modalities for each sign: RGB, depth, and skeleton joint points.

##### Category 3: Continuous Datasets

- Tubaiz, Shanableh, & Assaleh Dataset

This available upon-request dataset [95] employs an 80-word lexicon to create 40 sentences with no restrictions on sentence structure or length. Using two DG5-VHand data gloves, the sentences are recorded. The DG5-VHand glove is equipped with five integrated bend sensors and a three-axis accelerometer, enabling the detection of both hand orientation and movements. The gloves are suitable for wireless operations and rely on batteries. Information is transmitted, and sensor readings are collected between the gloves and a Bluetooth-connected PC. Subsequently, the sentences that were obtained were divided and assigned labels. A female volunteer, age 24, who is right-handed, offered to repeat each sentence ten times. A total of 33 sentences need both hands for gesturing, whereas the gestures for seven sentences can be made with just the right hand.

- Hassan, Assaleh, and Shanableh Sensor-Based Dataset

An 80-word lexicon was utilized to create 40 sentences in this available upon-request dataset [96], with no restrictions on sentence structure or length. Two Polhemus G4 motion trackers were employed to gather this dataset. These trackers offer six measurements: roll (a, e, r) and azimuth, elevation, and coordinates for the Euler angle. Volunteers to symbolize the signs included two signers.

- Hassan, Assaleh, and Shanableh Vision-Based Dataset

This available upon-request dataset [95] uses an 80-word lexicon to create 40 sentences with no restrictions on sentence grammar or length. This dataset was obtained solely with a camera; no wearable sensors were employed in the process. There was just one signer who offered to perform the signs.

##### Category 4: Miscellaneous Datasets

- mArSL

**Figure 13:**
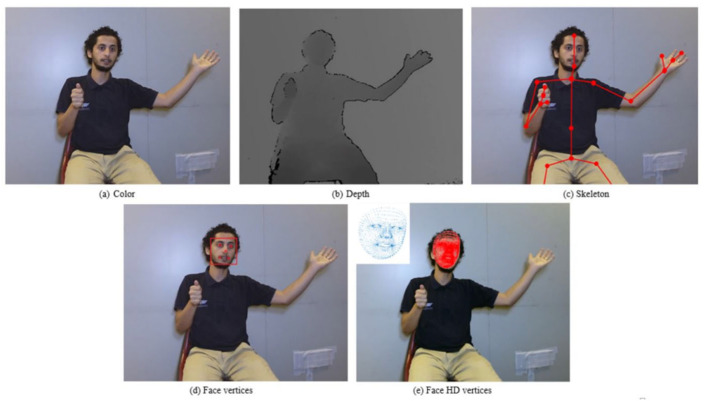
The mArSL dataset, an example of the five modalities provided for each sign sample [88].

Five distinct modalities—color, depth, joint points, face, and faceHD—are provided in the multi-modality ArSL dataset [88,89]. It is composed of 6748 video samples captured using Kinect V2 sensors, demonstrating fifty signs executed by four signers. Both manual and non-manual signs are emphasized. An example of the five modalities offered for every sign in mArSL is presented in Figure 13.

- KArSL-100

There are 100 static and dynamic sign representations in the KArSL-100 dataset [86,87]. A wide range of sign gestures were included in the dataset: 30 numerals, 39 letters, and 31 sign words. For every sign, there were three experienced signers. Each signer repeated each sign 50 times, resulting in an aggregate of 15,000 samples of the whole dataset (100 × 3 × 50). For every sign, there are three modalities available: skeleton joint points, depth, and RGB.

- KArSL-190

There are 190 static and dynamic sign representations in the KArSL-190 dataset [86,87]. The dataset featured a variety of sign gestures, including digits (30 signs), letters (39 signs), and 121 sign words. A broad spectrum of sign gestures was incorporated into the dataset, including 121 sign words, 30 number signs, and 39 letter signs. Each sign was executed by three skilled signers. Each signer repeated each sign 50 times, resulting in 28,500 samples of the dataset (190 × 3 × 50). For each sign, there are three modalities available: skeleton joint points, depth, and RGB.

- KArSL-502

Eleven chapters of the ArSL dictionary’s sign words, totaling 502 static and dynamic sign words, are contained in the KArSL dataset [86,87]. Numerous sign gestures were incorporated in the dataset, including 433 sign words, 30 numerals, and 39 letters. For every sign, there were three capable signers. Every signer repeated each sign 50 times, resulting in 75,300 samples of the dataset (502 × 3 × 50). Each sign has three modalities: RGB, depth, and skeletal joint points.

It is worth mentioning that public non-ArSL datasets were also utilized in a number of reviewed studies along with ArSL datasets [56,71,78,82,90]. The purpose of these studies was to apply the proposed models to these publicly available datasets and compare the results with published work on the same datasets.

#### 3.1.3. RQ1.3 How Many Signers Were Employed to Represent the Signs in Each Dataset?

**Figure 14:**
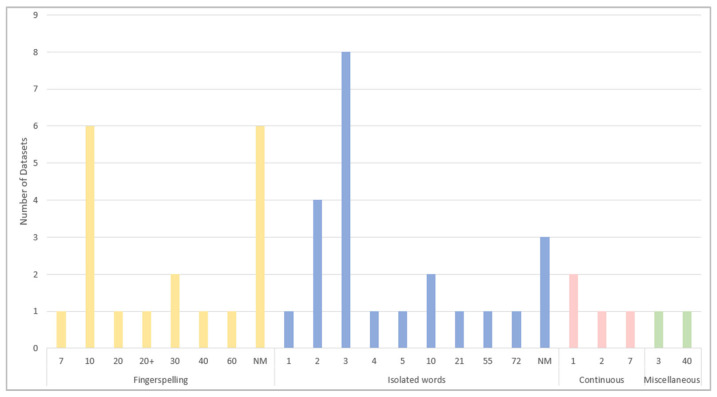
Number of datasets according to the number of signers.

The number of signers is one of the factors that impact the diversity of the datasets. In the reviewed studies, the number of signers varies from one dataset to another. The results show that the minimum number of signers is one in three datasets, whereas the highest number of signers is 72 in only one dataset, an isolated word dataset. Figure 14 shows that the majority of the ArSL datasets, with around 60%, recruit between one and ten signers. More diversity is provided by ten ArSL datasets that are executed by 20 to 72 signers. The number of signers who executed the signs is not mentioned or specified (NM) in any of the other nine ArSL fingerspelling and isolated datasets.

#### 3.1.4. RQ1.4 How Many Samples Are There in Each Dataset?

**Figure 15:**
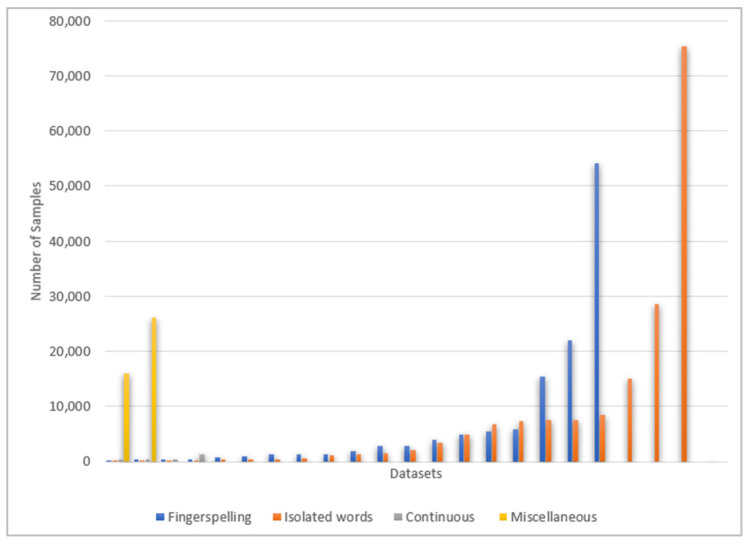
Number of samples in the datasets of reviewed papers for each dataset category.

Training machine learning and deep learning algorithms require the presence of datasets with a high volume of data or samples. Table 9, Table 10, Table 11 and Table 12 show the number of samples in each of the reviewed datasets. As Figure 15 reveals, a high number of samples are found in the datasets that belong to the category of isolated words. The biggest dataset in terms of the number of samples among all the reviewed datasets is KArSL-502 [86,87], with 75,300 samples. The second-biggest dataset is ArSL2018 [50,51], the fingerspelling dataset, which contains 54,049 samples. This is followed by the datasets that represent isolated words and fingerspelling. The dataset with the lowest sample size, 50, is the dataset for five facial expressions [80], which belongs to the category of isolated words.

#### 3.1.5. RQ1.5 How Many Signs Are Represented by Each Dataset?

**Figure 16:**
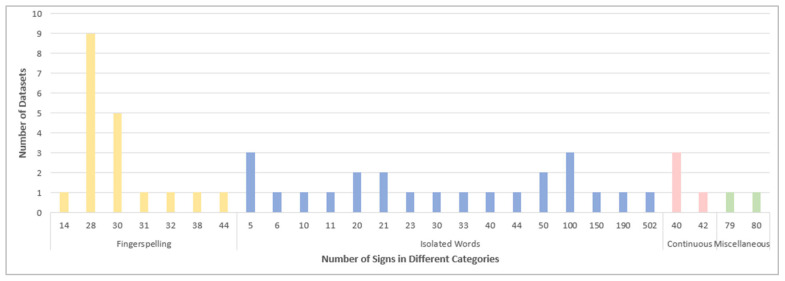
Number of datasets for each number of signs in different categories.

The number of signs differs based on the dataset category, as illustrated in Figure 16. In fingerspelling datasets, the number of signs ranges from 14 letters representing the openings of the Qur’anic surahs [46,47] to 38 representing letters and numbers [42], and 44 representing letters, numbers, and none [71]. The signs for basic Arabic letters, 28 letters, are represented by eight datasets. The remaining datasets in this category consist of signs for 30, 31, and 38 basic and extra Arabic letters. Interestingly, the highest number of signs is 502 in an isolated words dataset [86,87], as well as the lowest number of signs, five, is found in isolated words datasets [80,81,93].

#### 3.1.6. RQ1.6 What Is the Number of Datasets That Include Manual Signs, Non-Manual Signs, or Both Manual and Non-Manual Signs?

**Figure 17:**
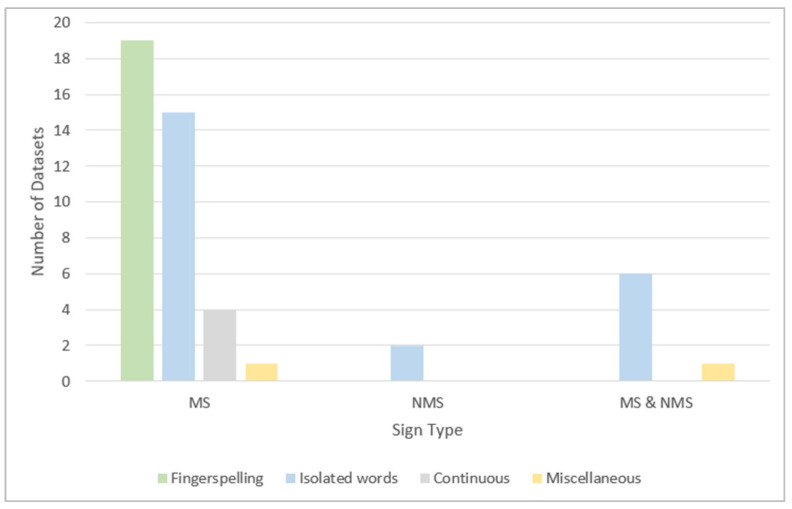
Number of datasets representing manual signs (MS), non-manual signs (NMS), and both signs in each dataset category.

Table 9, Table 10, Table 11 and Table 12 show the type of sign captured in each reviewed dataset. The type of sign can be a manual sign (MS), represented by hand or arm gestures and motions and fingerspelling; non-manual signs (NMS), such as body language, lip patterns, and facial expressions; or both MS and NMS. Figure 17 illustrates that the majority of the signs in all categories—except miscellaneous—are manual signs. In fingerspelling and continuous sentence datasets, all the signs are manual. Non-manual signs are represented by only two isolated word datasets. Both sign types are represented by three isolated word datasets and one miscellaneous dataset.

#### 3.1.7. RQ1.7 What Are the Data Acquisition Devices That Were Used to Capture the Data for ArSLR?

Datasets for sign language can be grouped as sensor-based or vision-based, depending on the equipment used for data acquisition. Sensor-based datasets are gathered by means of sensors that signers might wear on their wrists or hands. Electronic gloves are the most utilized sensors for this purpose. One of the primary problems with sensor-based recognition methods was the need to wear these sensors while signing, which led researchers to turn to vision-based methods. Acquisition devices with one or more cameras are usually used to gather vision-based datasets. One piece of information about the signer is provided by single-camera systems, such as a color video stream. Multiple cameras, each providing distinct information about the signer, such as depth and color information, are combined to produce a multi-camera gadget. One of these devices that can provide different types of information, like color, depth, and joint point information, is the multi-modal Kinect.

**Figure 18:**
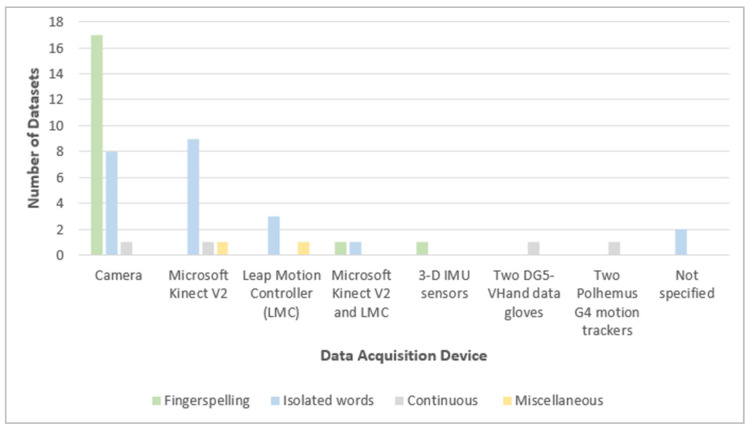
Data acquisition devices used to capture the data in the ArSL dataset.

The majority of the reviewed ArSL datasets use cameras to capture the signs, followed by Kinect and leap motion controllers (LMC), as shown in Figure 18. Wearable sensor-based datasets [70,95,96] use devices like DG5-VHand data gloves, Polhemus G4 motion trackers, and 3-D IMU sensors to capture the signs. These three acquisition devices are the least used among all the devices due to the recent tendency to experiment with vision-based ArSLR systems. No devices were specified in two of the datasets.

#### 3.1.8. RQ1.8 What Are the Acquisition Modalities Used to Capture the Signs?

**Figure 19:**
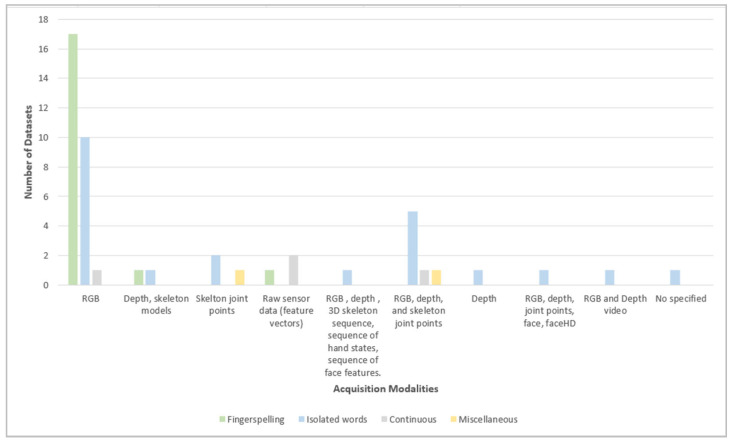
Acquisition modalities used to capture the signs in the ArSL dataset.

As depicted in Figure 19, the most popular acquisition modality for the datasets that belong to fingerspelling and isolated categories is RGB. Raw sensor data (feature vectors) is mostly used in continuous datasets, whereas RGB, depth, and skeleton joint points are the most popular in the category of isolated word datasets. The least used acquisition modalities for fingerspelling datasets are depth, skeleton models, and raw sensor data. For the other dataset categories, the least used modalities are distributed among different types of acquisition modalities.

#### 3.1.9. RQ1.9 Do the Datasets Contain Images, Videos, or Others?

**Figure 20:**
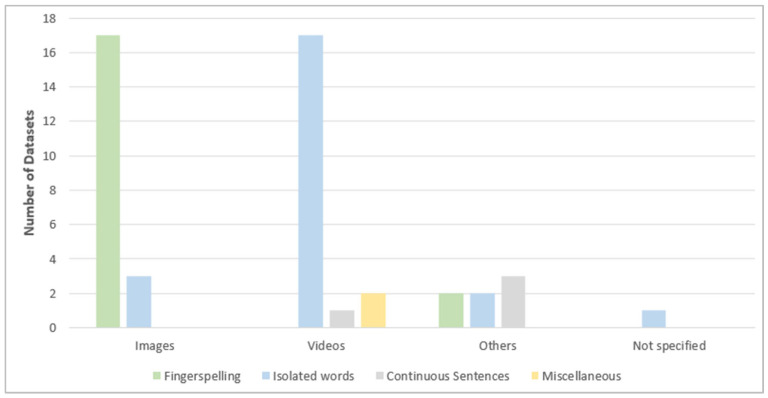
Contents of ArSL datasets, images, videos, or others.

As illustrated in Figure 20, almost all fingerspelling datasets—except two—contain images. Videos are the content of around 74% of the isolated datasets, followed by images and others. Only one continuous dataset contains videos, while the remaining continuous datasets contain different content from images and videos, such as sensor readings. All the miscellaneous datasets contain videos only.

#### 3.1.10. RQ1.10 What Is the Percentage of the Datasets That Represent Alphabets, Numbers, Words, Sentences, or a Combination of These?

**Figure 21:**
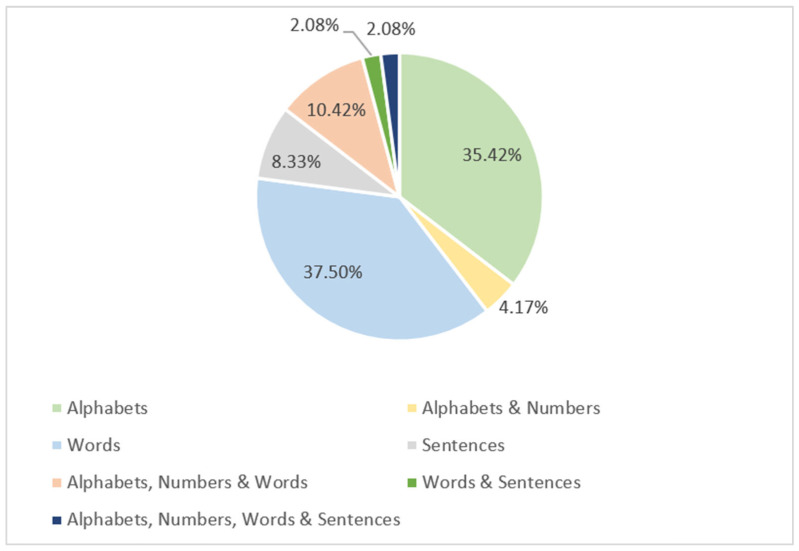
Percentages of alphabets, numbers, words, sentence datasets, and combinations of them.

ArSL datasets can be used to represent alphabets, numbers, words, sentences, or combinations of them. As demonstrated in Figure 21, the highest percentage of the reviewed ArSL datasets, roughly 37.50%, constitute words. This is followed by 35.42% of the datasets that represent alphabets. Combinations of alphabets, numbers, and words are represented by 10.42%. A minimum number of ArSL datasets are used to represent sentences, alphabets and numbers, words and sentences, and alphabets, numbers, words, and sentences with percentages of 8.33%, 4.17%, 2.08%, and 2.108%, respectively.

#### 3.1.11. RQ1.11 What Is the Percentage of the Datasets That Have Isolated, Continuous, Fingerspelling, or Miscellaneous Signing Modes?

**Figure 22:**
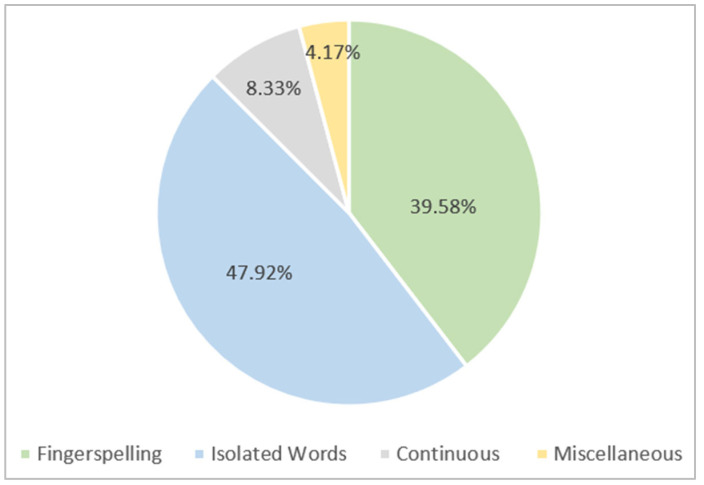
Percentages of the datasets based on the signing mode, fingerspelling, isolated, continuous, and miscellaneous.

With a percentage of 47.92%, isolated mode is regarded as the most prevalent mode, followed by fingerspelling signing (39.58%) and then continuous signing mode (8.33%), as shown in Figure 22. With 4.17%, the category involving miscellaneous signing modes has the least amount of work accomplished in this area.

#### 3.1.12. RQ1.12 What Is the Percentage of the Datasets Based on Their Data Collection Location/Country?

**Figure 23:**
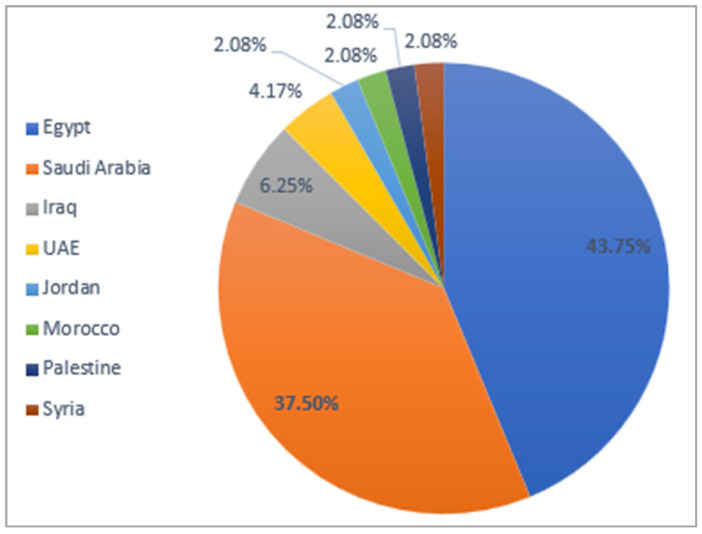
Distribution of the datasets based on their data collection location/country.

Figure 23 illustrates the distribution of the ArSL dataset collection across different countries. The figure shows that Egypt is the largest contributor to the ArSL dataset collection, accounting for 43.75%. Saudi Arabia follows with a significant contribution of 37.50%. Together, these two countries make up the majority of the dataset collection, totaling 81.25%. Iraq and the UAE contribute moderately to the dataset, with 6.25% and 4.17%, respectively. Jordan, Morocco, Palestine, and Syria each contribute a small and equal share of 2.08%, highlighting their relatively minor involvement. This distribution suggests the need to expand dataset collection efforts to the underrepresented countries to ensure a broader and more balanced representation of ArSL datasets. It might also reflect potential gaps in resources or interest in ArSL-related initiatives in these regions.

### 3.2. Machine Learning and Deep Algorithms Used for Arabic Sign Language Recognition

To address the second research question, RQ2: “What were the existing methodologies and techniques used in ArSLR?” six sub-questions were explored. These questions examine different phases of the ArSLR methods discussed in the reviewed papers, including data preprocessing, segmentation, feature extraction, and the recognition and classification of signs. The answers to each sub-question are analyzed across various ArSLR categories, such as fingerspelling, isolated words, continuous sentences, and miscellaneous methods. Table 14, Table 15, Table 16 and Table 17 provide a concise summary of the reviewed studies for each category. Rows shaded in gray indicate methods that utilize wearable sensors.

#### 3.2.1. RQ2.1: Which Preprocessing Methods Were Utilized?

The data preprocessing phase plays an important role in ArSLR to prepare the data before feeding it into ML/DL models. Data preprocessing involves a series of operations that aim to improve the images/videos before they are used by the model, such as color space conversion, resizing and cropping, noise reduction, data normalization, and augmentation. The preprocessing techniques that have been employed by the reviewed studies are discussed in this section.

**Color Space Conversion:** Color spaces illustrate the encoding of the colors. RGB and grayscale are common for images, while HSV obtains more accurate color information for extracting information. It is essential to understand and select the appropriate color space for particular jobs. Color spaces provide distinct benefits; depending on the job requirements, conversion can enhance certain features or make analysis easier.

Converting RGB images to grayscale can simplify the image data and hence reduce computational load. Most of the research that applied greyscale conversion is categorized as fingerspelling ArSLR [41,46,47,49,53,55,68,72,84], followed by isolated words ArSLR [82,94], and miscellaneous ArSLR [38]. Other researchers in the category of fingerspelling ArSLR have converted from greyscale space to red, green, and blue (RGB) images [60] and to hue, saturation, and value (HSV) color space [62] to ensure compatibility with the classifier algorithms used, which entails more accurate results. Transformation to YCbCr space was only applied by two studies published in 2015, for fingerspelling ArSLR [42] and isolated words ArSLR [79], for the purpose of taking advantage of the lower resolution for color with respect to luminosity, which means faster processing.

**Resizing and Cropping:** Resizing images to a uniform size is essential to ensuring optimal performance of ML/DL models. It helps avoid computational loads and simplifies the process for the model to learn patterns uniformly across different samples. With cropping, the focus is on a specific region of an image, and any irrelevant details are removed. This maximizes the model’s capacity to recognize particular features, which is useful for tasks where a certain object or object’s location is crucial. In the context of ArSLR, the hands are considered important objects to crop, as implemented by a number of researchers [33,38,92]. In the reviewed papers, most of the researchers have resized their acquired data, such as images and video frames, to a certain size and then trained their ML/DL models based on that size [33,35,38,41,44,46,47,53,55,58,60,61,63,65,66,68,69,71,72,73,74,84,92,94]. Other researchers have utilized pre-trained models that require that the data be fed in a specific size. Therefore, the images were resized to fit the pre-trained model input layer [52].

**Normalization:** Normalization refers to all operations and processes meant to standardize the input according to a predetermined set of rules, with the ultimate goal being to enhance the ML/DL model’s performance. It may involve several statistical procedures or input processing operations. The ideal normalization process varies depending on different factors, such as the ML/DL model, the degree of variability in the sample, and the nature of the input, whether it is text, image, or video. Pixel values are usually normalized to have a mean of 0 and a standard deviation of 1. This process improves the model’s performance during training by maintaining values within a standardized range, which helps with convergence. Normalization was mostly applied to the category of fingerspelling ArSLR [42,44,46,47,53,55,56,57,61,65,71], followed by isolated words [71,79,82,92], and continuous ArSLR [95,97]. Sensor readings from the DG5-VHand data gloves were normalized using the z-score [95], and then the standard deviations and means of the readings of the training set were saved and used again to normalize the testing set. The normalization applied by Hisham and Hamouda [97] on the frames captured by Microsoft Kinect solves two main issues related to the variation of the signers’ position and size. It consequently yields more accurate feature extraction for the coordinates, regardless of the signers’ location or size.

**Data Augmentation:** The technique of artificially creating new data from preexisting data, known as data augmentation, is mostly used to train new ML/DL models. Large and diverse datasets are necessary for the training of the models; however, finding adequately varied real-world datasets can be difficult. Data augmentation involves making minor adjustments to the original data in order to artificially enlarge the dataset. Using data augmentation during model training helps avoid overfitting, which usually occurs when a model performs well on training data but poorly on unseen data. There are different techniques to implement data augmentation, including rotation, flipping, shearing, shifting, rescaling, translation, and zooming. Using any of these techniques depends mainly on the type of input and the characteristics of the model. As illustrated in Table 14, Table 15, and Table 17, various studies from the field of ArSLR utilize data augmentation techniques for the purpose of enhancing the quality of data and improving the model’s performance.

**Noise Reduction:** This process is used to remove or reduce unwanted noise in the data and irrelevant or redundant features to direct the model’s attention to the data’s most informative elements. In the reviewed papers, various noise reduction techniques have been used for this purpose, such as Gaussian filter [38,68,71,84], median filter [38,60,71,90], averaging filter [71], and weighted average filter [91]. The Pulse Coupled Neural Network (PCNN) signature was used to reduce the random noise and smooth images [80]. In a sensor-based ArSLR study [70], a low-pass filter was used to remove dynamic noise from vibrations and other external factors that affect acceleration readings, and a high-pass filter was used to remove low-frequency drift from gyroscope readings. Dataset cleaning was implemented by removing the observations that had null values [48,81] and the rows that had the same values [48].

#### 3.2.2. RQ2.2: Which Segmentation Methods Were Applied?

Segmentation is a process that divides an image into distinct groups of pixels, or image segments, that form the region of interest (ROI). There are three main types of image segmentation: semantic segmentation, instance segmentation, and panoptic segmentation. Semantic segmentation works by assigning a class label to every pixel in an image, making it possible to properly identify and classify objects based on their semantic significance. Instance segmentation entails identifying and delineating each individual object within an image. Its functionality is not only limited to identifying objects in an image but also precisely locating each instance of that object within its borders. Panoptic segmentation is complex and goes beyond classifying each pixel in an image according to its class label to identifying the instance of that class it belongs to. In vision-based ArSLR, hand segmentation is usually the main concern, followed by non-manual segmentation for facial expressions and body gestures. Various segmentation techniques are utilized for this purpose, including thresholding, edge detection, region-based segmentation, clustering, and neural network-based segmentation. In this section, various segmentation methods that have been utilized by the reviewed studies are discussed.

Threshold-based segmentation is the simplest technique that involves choosing a threshold value and classifying image pixels based on pixel intensity values. In the reviewed papers, a global threshold that requires choosing a single intensity value to divide the whole image into distinct regions was implemented by some studies [68,72,82]. Adaptive thresholding determines the threshold value for smaller regions. As a result, various threshold values for varying regions in relation to lighting change. Adaptive thresholding based on the gyroscope intensity was utilized to identify the beginning and the end of each gesture segment [70]. Other researchers applied adaptive thresholding to each video frame to capture the most important features out of the frames [76,94].

Edge-based segmentation is another technique that relies on discontinuity detection and is considered suitable for images that have high contrast between objects. Algorithms like Sobel, Canny, Laplacian, and Roberts edge detectors are used for this purpose. Two of the reviewed papers applied the Sobel algorithm, which detects the hand edges with no attention paid to the weak edges like the Canny algorithm and does not miss information about the hand shape, as in the Laplacian algorithm [59,72].

The region-based segmentation technique involves dividing the image into smaller homogeneous regions, which are then recursively merged based on predetermined attributes in intensity, texture, and color. Podder et al. adopted a simple and fast face and hand region segmentation method using MediaPipe Holistic [35]. With this approach, a segmented dataset is produced to be used for ArSL classification. Ahmed et al. applied skin filter technology to the keyframes for the purpose of separating the skin-colored pixels from the non-colored pixels, followed by hand crop technology [33].

Deep learning-based segmentation techniques such as Convolutional Neural Network (CNN) have improved image segmentation and produced remarkably accurate and efficient results. These powerful techniques adopt a hierarchical approach, applying several layers of filters to the input image for feature extraction. Recently, great success has been obtained in hand segmentation and gesture recognition using this technique. Most reviewed ArSLR studies published in 2020 onwards have utilized this technique to help boost their results. CNNs are adopted in this field, wherein various CNN architectures are trained from scratch [38,39,46,53,55,56,57,69,78,94].

Transfer learning in CNN involves utilizing the early and central layers while only retraining the last layers on a different set of classes. The model leverages labeled data from its original training task, hence reducing training time, improving neural network performance, and functioning well with limited data. Examples of the pre-trained models used in transfer learning include VGG, AlexNet, MobileNet, Inception, ResNet, DensNet, SqueezeNet, EfficientNet, CapsNet, and others. Many researchers in the field of ArSLR used this approach for segmentation [52,54,58,59,60,61,62,63,64,65,66,71,73,74,90,91,92,93,98]. Alharthi and Alzahrani utilized two pretrained vision transformers, ViT (ViT_b16, ViT_132) and Swin (SwinV2Tiny256) [64]. In ViT, an image is considered a series of patches [99]. The image is then split into small patches, and a 1D vector is created from each patch. The transformer model receives these patch embeddings as input. The model can focus on various patches while tracking the connections between them due to the self-attention mechanism. It assists in the model’s understanding of the image’s context and interdependencies. Positional embeddings, which offer details about the spatial placement of each patch, are also part of ViT. Built upon the Transformer architecture, Swin processes and comprehends sequential data using a multi-layered system of self-attention mechanisms [100]. Swin has created shifted windows that operate by partitioning the input image into smaller patches or windows and shifting them throughout the self-attention process. By using this method, Swin can handle huge images quickly and effectively without having to rely on computationally demanding processes like convolutional operations or sliding window mechanisms. Swin increases the receptive field to gather global dependencies and improves the model’s comprehension of the visual context by shifting the patches.

Aly and Aly addressed hand segmentation using the state-of-the-art semantic segmentation DeepLabv3C model, which is built on Resnet-50 as a backbone encoder network with atrous spatial pyramid pooling [77]. Using the DeepLabv3C mask image, hand areas are cropped from each corresponding frame of the input video sequence, and the resulting mask image is then normalized to a fixed size for scale invariance. Alawwad et al. used fast R-CNN to enhance the efficiency and speed of the original model, R-CNN, by integrating a Region Proposal Network (RPN) along with an ROI pooling layer, which reduces the processing time and contributes to overall better performance [73].

#### 3.2.3. RQ2.3: Which Feature Extraction Methods Were Used?

Feature extraction is carried out by converting important parts of the input data to sets of compact feature vectors. In the field of sign language recognition, the features that are extracted from the input hand gestures should contain pertinent information and be displayed in a form that helps distinguish the sign that needs to be classified from other signs. The most common methods of feature extractions include Shift-Invariant Feature Transform (SIFT), Speeded Up Robust Feature (SURF), Principal Component Analysis (PCA), Linear Discriminant Analysis (LDA), Convexity defects and K-curvature, and features extraction in the frequency domain.

Features extraction in the frequency domain is adopted by a sensor-based ArSLR system [70], where a collection of well-constructed features was suggested to create the feature vectors for the detected segments for the purpose of training the recognition model. Time-Domain (TD) and Frequency-Domain (FD) signal characteristics are contained in the feature set. On each of the three axes (x, y, and z) of the gyroscope and accelerometer sensors, these features were extracted. Time-domain features are significant due to how they depict the change of signals over the course of time, whereas the frequency features highlight the salient features and signal recurrence of each component. To compute the frequency spectrum of the discrete signal readings, the Discrete Fourier Transform (DFT) was used. The extracted feature set comprises a total of nine time-domain features and nine features achieved from the autocorrelation and frequency. Another glove-based study for the recognition of continuous ArSL by Tubaiz et al. [95] employed a feature extraction technique that reflects the temporal dependence of the data. In this technique, a sliding window is used to compute the mean and standard deviations of the sensor readings. The accuracy of the classification is affected by the sliding window’s size. A small window size is insufficient to fully convey the present feature vector’s context. The context becomes increasingly noticeable as the size expands until it becomes saturated. Classification accuracy suffers when window size is increased further. Hassan et al. [96] applied a window-based approach to glove-based data and a 2D Discrete Cosine Transform (DCT) to vision-based datasets. In 2D DCT, the feature extraction relies on two parameters to be specified. The first parameter is cutoff, which is the number of DCT coefficients to keep in a feature vector. The more coefficients there are, the higher the recognition rate. However, recognition rates generally decline when the feature vector’s dimensionality rises above a particular threshold; hence, there is typically a threshold beyond which any increase in the DCT cutoff will result in a decline in recognition rates. The weighting parameter x is the second parameter that needs to be specified empirically. When 100 DCT coefficients and the value of x = 1 were used, the highest classification rate was obtained.

Elatawy et al. [68] developed an alphabet ArSLR system using the neutrosophic technique and fuzzy c-means. They proposed to use the Gray Level Co-occurrence Matrix (GLCM) to extract features from neutrosophic images. Three matrices—object (T), edge (I), and background (F)—are used to describe neutrophilic images. The GLCM works by scanning the image and recording the gray levels of each pair of pixels that are spaced apart by a set direction (0 and distant). Pixels and their neighbors are, hence, the basis for GLCM’s feature extraction process for neutrosophic images. The contrast, homogeneity, correlation, and energy are the calculated GLCM parameters. From each image, a total of 12 features, consisting of 4 GLCM parameters for each image component T, I, and F, are extracted.

Hybrid feature extraction has been utilized in various studies to overcome the limitations of single techniques and benefit from the advantages. Tharwat et al. [43] used SIFT to extract the invariant and distinctive features and LDA to reduce dimensions and thus increase the system’s performance in recognizing ArSL letters. A study conducted by Ahmed et al. [33] to recognize the ArSL isolated dynamic gestures proposed a feature integration between intensity histogram features and GLCM. The former contains six features that represent the first-order statistical information about the image, such as mean, variance, skewness, kurtosis, energy, and entropy, and the latter consists of 23 features to represent the second-order statistical information about the image features, like contrast, homogeneity, dissimilarity, angular second moment, energy, and entropy. The combined integrated features vector comprises 26 features since three features are shared by both.

Other researchers have investigated the feature extraction techniques by conducting a comparison between them to measure their performance. Sidig et al. [76] compared different feature extraction techniques: Modified Fourier Transform (MFT), Local Binary Pattern (LBP), Histogram of Oriented Gradients (HOGs), and combination of Histogram of Oriented Gradients and Histogram of Optical Flow (HOG-HOF) for isolated word ArSL recognition with Hidden Markov Model (HMM) for classification. With MFT and HOG, the best accuracy was achieved. In a previous study, Alzohairi et al. [49] conducted a comparison between five texture descriptors for the purpose of ArSL alphabet recognition. The descriptors are HOG, Edge Histogram Descriptor (EHD), GLCM, Discrete Wavelet Texture Descriptor (DWT), and LBP. These texture descriptors include details on the edges of the image as well as region homogeneity. The comparison results reveal that the HOG descriptor outperforms the other descriptors. Agab and Chelali [41] applied individual descriptors, including DWT, the Dual Tree Complex Wavelet Transform (DT-CWT), HOG, and two combined descriptors, namely DWT + HOG and DT-CWT + HOG, when compared to the individual descriptors, the combined descriptors DT-CWT + HOG outperformed with respect to accuracy rate and execution times.

In addition to the feature extraction methods discussed above, other researchers employed widely used deep learning techniques, such as CNNs, to extract pertinent features. These techniques extract features in the first layers and then feed them into the subsequent layers. CNNs have been utilized to extract features from fingerspelling ArSL images [46,47,53,55,56,57,69,94] and video frames [94]. CNNs have been used in combination with Long Short-Term Memory (LSTM) in order to extract spatial and temporal data dependencies [78], where the CNN model was adopted to extract features from each video frame separately and LSTM to learn the temporal features across video frames. Aly and Aly [77] have utilized the Convolutional Self-Organizing Map (CSOM) to extract hand-shape features from video frames and the Bi-directional Long Short-Term Memory (BiLSTM) to model the temporal dependencies in the video sequences.

In transfer learning, pre-trained models, such as VGG, ResNet, AlexNet, and Inception, have been trained on large-scale image datasets like ImageNet. These models function as effective feature extractors, converting raw images into forms that contain significant details about the visual material. By extracting features from pre-trained neural models, the information gathered from massive datasets is used to improve the performance of others’ work. Most of the studies conducted in 2019 onwards in the context of fingerspelling ArSLR have relied on pre-trained models to extract the features. Most of these studies have experimented with and compared various models [52,54,58,59,62,63,64,65,73]. Ismail et al. [71] have compared the performance of various single models and multi-models in feature extraction and ArSL recognition.

A few researchers have implemented solo pre-trained models, for example, lightweight EfficientNet with different settings [74] and EfficientNetB3 [66]. Islam et al. [66] have leveraged EfficientNetB3 to extract initial features and adopted stacked autoencoders to further refine these features. Alnuaim et al. [60] implemented two models, ResNet50 and MobileNetV2, together. In isolated and continuous ArSLR, various studies investigated the implementation of different pre-trained models accompanied by Recurrent Neural Networks (RNNs) or LSTM to extract spatial and temporal features accurately [35,88,90,92,98]. A few researchers have relied on single models to be feature extractors to generate a set of feature vectors, such as a capsul neural network (CapsNet) [91] and the DenseNet169 model [93].

#### 3.2.4. RQ2.4: What Algorithms Were Used for ArSLR?

Different ML/DL algorithms have been utilized in the field of ArSLR, including HMM, Support Vector Machines (SVM), K-Nearest Neighbor (KNN), Random Forest (RF), CNNs, CNN-based pre-trained models, RNNs, LSTM, and others.

##### Machine Learning

Machine learning (ML) is a branch of artificial intelligence (AI) that enables machines to learn from data and make decisions or predictions without being specifically programmed to do so. Fundamentally, machine learning is concerned with developing and applying algorithms that help with these decisions and predictions. As they handle more data, these algorithms are built to perform better over time, becoming more precise and effective. In the following, ML algorithms employed in the reviewed ArSLR research papers are discussed.

HMM is a powerful statistical modeling method that can identify patterns in the complicated relationships between actions in a continuum of time and space. HMM has been applied to the field of ArSLR by different studies to classify static hand gestures of ArSL alphabets [42], isolated words [76,79], and continuous sentences [96]. Abdo et al. [42] modeled the information of each sign with a different HMM. The model with the highest likelihood was chosen as the best model, and the test sign was classified as the sign of that model. The HMM was applied to the self-acquired dataset, ArASLRDB, with 29 Arabic alphabet signs. The recognition system was tested when splitting the rectangle surrounding the hand shape into 4, 9, 16, and 25 zones. The optimal number of zones was determined to be 16, with 19 states that recognize the Arabic alphabet of sign language. The algorithm could reach a 100% recognition rate by increasing the zone number to 16 or more, but it would take more time. To recognize ArSL at the word level, Abdo et al. [79] proposed to utilize the Enhancement of Motion Chain Code (EMCC) that uses HMM. The recognition rate achieved outperformed other systems by 98.8% when applied to a private, self-gathered dataset of 40 ArSL words. Hassan et al. [96] compared two classification algorithms, HMM (RASR and GT^2^K toolkits) and Modified K-Nearest Neighbor (MKNN), which are adequate for sequential data on sensor-based and vision-based datasets. Despite the high recognition rates obtained by the RASR and GT^2^K HMM toolkits, MKNN has the best sentence recognition rates, exceeding both HMM toolkits.

The MKNN algorithm was first proposed by Tubaiz et al. [95] in 2015 to classify sequential data for a glove-based ArSL. In this modification, the context prior to predicting the label of each feature vector is considered. It relies on using the most prevalent label within a surrounding window of labels to replace the predicted label. Once every label in a given sentence has been predicted, the statistical mode of the labels that surround it is used to replace each label. KNN is a non-parametric supervised machine learning algorithm that has been adopted by a number of ArSLR researchers [37,43,48,72,85,97]. By calculating the distances between unknown patterns and each sample, the KNN classifier can recognize unknown patterns based on how similar they are to known samples. The K-nearest samples are then chosen as the basis for classification. Among the K-nearest samples, the class with the greatest number of samples is assigned the unknown pattern. It has been noticed that the accuracy results of KNN for the miscellaneous ArSLR [37] or continuous sentence recognition [97] outperformed other algorithms when compared. Hisham and Hamouda [37] carried out a comparison between different algorithms, including SVM, KNN, and ANN, for static and dynamic gestures depending on two different feature sets: palm features set, and bone features set. KNN obtained the best accuracy results for the static gestures, achieving 99% and 98% for the two sets, respectively. In another study conducted by Hisham and Hamouda [97], the experimental results revealed that the accuracy of KNN with majority voting outperformed the other algorithms in recognizing dynamic medical phrase signs. On the other hand, when KNN was compared with other algorithms, including SVM and nearest neighbor (minimum distance) [43], SVM and RF [48] for fingerspelling ArSLR, and different variations of SVM and RF for isolated word recognition [85], the findings indicated that the best performance was achieved by SVM. In the context of fingerspelling recognition, just one study [68] demonstrates that the KNN algorithm outperformed other algorithms, including C4.5, Naïve Bayes (NB), and Multilayer Perceptron (MLP), in terms of accuracy.

SVM is a supervised machine learning approach that is mainly used for regression and classification tasks. The SVM algorithm works by finding the optimal hyperplane that maximizes the distance between each class in an N-dimensional space in order to classify data. SVM is one of the popular algorithms that have been used for different categories in the field of ArSLR, including fingerspelling recognition [41,43,48], isolated recognition [85], continuous recognition [97], and miscellaneous ArSL recognition [37]. The performance of the SVM algorithm proved to be outstanding when compared to other algorithms in fingerspelling and isolated recognition. Tharwat et al. [43] carried out a number of experiments to compare different ML algorithms, which showed that the performance of the SVM was inferior to that of the KNN and minimum distance in fingerspelling recognition with around 99% accuracy. While the experiments proved that the SVM algorithm is robust against any rotation, achieving 99% accuracy, the performance needs to be improved in the case of image occlusion. A different approach was proposed by Almasre and Al-Nuaim [48], where two stages of classification were carried out using SVM, KNN, and RF. Stage 1 involved training the three classifiers on the original dataset. Stage 2 entailed training the classifiers on an ensemble dataset, where the output of each classifier was coupled with an ensemble schema dataset to reclassify the classes. To see if changing numbers affected the performance of the classifiers, different observations for each letter were evaluated. When used as a standalone classifier, SVM yielded a superior overall accuracy of 96.119%, regardless of the number of observations. SVM would be a more efficient option because it requires less complexity while obtaining higher accuracy. Agab and Chelali [41] proposed a static and dynamic hand gesture recognition system that adopts the combined feature descriptors DT-CWT + HOG and compared the classification performance of three Artificial Neural Networks (ANNs), MLP, Probabilistic Neural Network (PNN), Radial Basis Neural Network (RBNN), SVM, and RF. Four distinct datasets comprising alphabet signs and dynamic gestures, including alphabet ArSL, were used for the experimental evaluation. The SVM classifier performed better for the ArSL dataset with regard to recognition rates and processing time. To recognize ArSL gestured dynamic words, Almasre and Al-Nuaim [85] proposed a dynamic prototype model (DPM) using Kinect as an input device. A total of eleven predictive models based on three algorithms, namely SVM, RF, and KNN, with varying parameter settings, were employed by the DPM. According to research findings, SVM models using a linear kernel and a cost parameter of 0.035 were able to attain the maximum accuracy for the dynamic words gestured. Alzohair et al. [49] developed a model employing a one-versus-all SVM classifier for each gesture. In their model, one class for each ArSL alphabet gesture was considered, and thirty classes resulted from this. A model is learned for each gesture by training the classifier using one particular class against all the others. The one-versus-all strategy looks for a hyperplane that differentiates the classes by considering all classes and splitting them into two groups, one for the points of the class under analysis and another for all other points.

The RF algorithm is a popular tree-learning approach in machine learning. During the training stage, it generates a collection of decision trees. To measure a random subset of characteristics in each partition, a random subset of the data set is used to build each tree. Because each tree is more variable as a result of the randomization, there is less chance of overfitting, and overall prediction performance is enhanced. In predictions, the algorithm averages (for regression tasks) or votes (for classification tasks) the output of each tree. The findings of this cooperative decision-making process, which is aided by the insights of several trees, are consistent and accurate. Random forests are commonly utilized for classification and regression tasks because of their reputation for managing complex data, minimizing overfitting, and producing accurate predictions in a variety of settings. A few studies have compared the RF performance to other ML algorithms for the sake of fingerspelling recognition [41,48] and isolated recognition [85]. The comparative results showed that while RF did not obtain the best recognition accuracy, it outperformed all other classifiers in terms of recognition rates for non-ArSL datasets, such as the ASL, Marcel, and Cambridge datasets [41]. Elpeltagy et al. [82] proposed to use the Canonical Correlation Analysis (CCA) [101] and RF algorithms for isolated word recognition. The proposed approach is based on hand shape and motion, where HOG-PCA is used for hand shape description, CCA for hand shape matching, Cov3DJ+ for motion and feature description, and RF for motion classification. The classification starts by applying the RF to the Cov3DJ+ descriptor in order to determine which top sign corresponds to the highest T probabilities. Subsequently, the CCA is used to determine which of these top signs is right by applying it to the hand-shape descriptors that correspond to them. CCA enhances classification performance by combining data from various performers and repetitions.

Deriche et al. [83] proposed the use of dual LMCs to capture the signer performing isolated Arabic dynamic word signs. The Gaussian Mixture Model (GMM) and a Bayesian classifier were used to examine the features that were extracted from the two LMCs. The individual Bayesian classifier findings were aggregated through an evidence-based methodology, specifically the Dempster-Shafer (DS) theory of evidence. A medium-sized vocabulary (100 signs) comprising signs frequently used in social settings was utilized to evaluate the suggested method. A simple LDA-based method to examine the system’s performance across several classifiers was employed. The results demonstrate that the combination strategy based on DS theory performs approximately 5% better than the LDA-based approach. About 92% recognition accuracy was attained.

The Euclidean distance classifier has been utilized in two isolated word recognition studies [5,33]. Euclidean distance measures the similarity between two feature vectors that are built directly from geometric features of the manual signs [33] or both manual and non-manual signs [5]. The experimental results show that the proposed system by Ahmed et al. [33] recognizes signs with an accuracy of 95.8%. Better outcomes were achieved in the Ibrahim et al. study, where the system demonstrated its resilience against various occlusion scenarios and reached a recognition rate of 97% in signer-independent mode [5].

Similar images are grouped together in the image clustering phase. In clustering problems, the fuzzy c-means approach is frequently employed. It is a clustering technique that gathers every data pixel into two or more clusters. The membership of the data changes to point in the direction of the designated cluster center as it moves closer to it. The Euclidean distance can then be used to calculate the degree of fuzziness between the cluster centers. This approach has been employed by Elatawy et al. [68] to recognize the Arabic alphabet sign language after converting the images to the neutrosophic domain and extracting their features. According to the experimental evaluation, the fuzzy c-means approach resulted in a 91% recognition accuracy rate.

Dynamic Time Warping (DTW) is known as an optimal alignment algorithm between two given sequences. DTW is used in many domains to quantify the similarity between two sequences that are changing in speed or time. Because it can handle the speeds at which signs are performed, the DTW is very appropriate for tasks involving sign recognition. This involves utilizing DTW to compare a set of frames from the training set with a set of frames from the test set. Each set of frames will be considered a signal or pattern. To find the similarity of the sequences that will be compared, they need to be warped non-linearly in the time dimension, independent of some non-linear changes in the time dimension. The sequence in the training set with the shortest DTW distances is the most similar sequence to the test sequence, as identified by the DTW based on the estimated distance between the most similar group and the test sign. At last, it selects the group that is the most comparable and assigns it to the test sign. Hisham and Hamouda [37] used LMC as an input device and employed this algorithm for dynamic gestures. The study findings showed that DTW dominated other models, including KNN, SVM, and ANN, for both the palm feature set and the bone feature set, with accuracy of 97.4% and 96.4%, respectively. When DTW was used for continuous sign recognition captured by Kinect [97], the performance was worse than the other models, KNN, SV, and ANN, in terms of accuracy and response time.

##### Deep Learning

The field of deep learning is concerned with learning data representations. However, the intricacy of the models and the underlying features of the system’s input restrict the ability of deep learning techniques to capture semantics embedded within data. The advances in deep learning have improved sign language recognition accuracy and effectiveness, leveraging ANNs, CNNs, and RNNs. An ANN is made up of several perceptrons or neurons at each layer. Because an ANN only processes inputs in a forward manner, it is often referred to as a feed-forward neural network. One of the most basic varieties of neural networks is this kind of network. Information is passed through a number of input nodes in a single direction until it reaches the output node. The network’s operation can be better understood whether or not it has hidden node layers. Hidden node layers may or may not exist in the network, which would make its behavior easier to understand. ANN did not show high performance when compared to other ML algorithms for static gestures representing letters, numbers, and words [37]. A similar result was achieved when applying ANN in the context of continuous ArSLR [97].

MLP is a feed-forward ANN with at least three layers of neurons: input, output, and hidden. The MLP’s neurons usually employ fully connected neurons’ nonlinear activation functions, which enable the network to recognize input with complicated patterns. Similar to MLP, RBNN is a feed-forward neural network that has a single hidden layer made of nonlinear radial basis functions (RBF), like a Gaussian function. Each neuron calculates the distance between the input data and the function’s center; at shorter distances, the neuron’s output value increases. PNN is another feedforward neural network that is frequently utilized to address pattern recognition and classification issues [102]. A PNN classifier is an application of Bayesian network and kernel discriminate analysis that develops a family of probability density function estimators. Agab and Chelali [38] conducted a comparison between five different classifiers, including three variants of the ANN, which are MLP, PNN, and RBNN, as well as SVM and RF, for the purpose of fingerspelling recognition. The results demonstrate lower performance of the ANN’s variants compared to the SVM and RF algorithms. A similar low performance for MLP was obtained by another fingerspelling recognition study [72] when comparing MLP with C4.5, NB, and KNN.

Two studies were carried out using MLP for the purpose of isolated word recognition [80,93]. ElBadawy et al. [80] proposed using MLP to classify manual sign input and a PCA network followed by an MLP network for facial expressions and body movements. Due to the integrated modules for body movement and facial expression recognition, the system achieved an accuracy of 95% for a dataset with 20 dynamic signs. Al-Onazi et al. [93] utilized the MLP classifier for sign recognition and classification in order to identify and categorize the presence of sign language gestures for five words. The Deer Hunting Optimization (DHO) algorithm is then applied to optimize the MLP model’s parameters. With an accuracy of 92.88%, the comparison analysis demonstrated that the proposed method produced better results for gesture classification than other methods.

A Deep Belief Network (DBN) is a class of deep neural networks used for unsupervised learning activities like generative modeling, feature learning, and dimensionality reduction. It is made up of several layers of hidden units that are trained to represent data in a structured way. Among the reviewed studies, only one paper was found to utilize the DBN approach paired with the direct use of tiny images to recognize and categorize Arabic letter signs [44]. By identifying the most significant features from sparsely represented data, deep learning was able to significantly reduce the complexity of the recognition problem. After scaling and normalization, a total of about 6000 samples of the 28 Arabic alphabetic signs were employed to extract features. A softmax regression was used to evaluate the classification process, and the results showed an overall accuracy of 83.32%, demonstrating the great reliability of the Arabic alphabetical letter recognition model based on DBN.

CNNs are the extended version of ANNs and one of the most widely utilized models in use nowadays. This neural network computational model comprises one or more convolutional layers that can be either pooled or fully connected. It is based on a variant of multilayer perceptrons. CNN’s ability to autonomously recognize key features without human oversight is by far its greatest advantage over its forerunners. Additionally, CNN delivers remarkable accuracy and processing efficiency. Numerous studies have used CNNs to recognize fingerspelling ArSL [46,47,53,55,56,57,69]. Althagafi et al. [53] developed a system that automatically recognizes 28 letters in Arabic Sign Language using a CNN model with a grayscale image as input [50,51]. Using 54,049 sign images [50,51], Latif et al. [55] offered various CNN architectures. Their results show how the size of the dataset has a significant impact on the proposed model’s accuracy. As the dataset size is increased from 8302 samples to 27,985 samples, the testing accuracy of the suggested model rises from 80.3% to 93.9%. When the dataset size is raised from 33,406 samples to 50,000 samples, the testing accuracy of the suggested model improves even further, rising from 94.1% to 95.9%. Alshomrani et al. [56] utilized CNNs to categorize the images into signs. CNN has been experimented with various settings for datasets containing Arabic and American signs. With an accuracy of 96.4%, CNN-2—which comprises two hidden layers—produced the best results for the Arabic sign language dataset [50,51]. Kamruzzaman [69] developed a vision-based method that uses CNN to recognize Arabic hand sign-based letters and transform them into Arabic speech with a 90% recognition accuracy. Utilizing the ArSL2018 dataset and a special ArSL-CNN architecture, Alani and Cosma [57] built a system for recognizing Arabic signs. While training, the suggested ArSL-CNN model’s accuracy was 98.80%; while testing, it was initially 96.59%. In order to lessen the impact that unbalanced data has on the model’s precision, they chose to use a range of resampling techniques for the dataset. The results show that the synthetic minority oversampling method (SMOTE) improved overall testing accuracy from 96.59% to 97.29%. During training, the proposed ArSL-CNN model’s accuracy was 98.80%, and roughly 96.59% during testing. In order to reduce the impact that unbalanced data has on the model’s accuracy, they opted to use a range of resampling techniques on the dataset. The results show that SMOTE boosted the overall testing accuracy from 96.59% to 97.29%. Abdelghfar et al. [47] proposed a new convolutional neural network-based model for Qur’anic sign language recognition, QSLR-CNN. A subset of the larger Arabic sign language collection, ArSL2018 [50,51], comprising just 24,137 images, was used for the tests. This subset represents the 14 dashed letters in the Holy Qur’an. The experiments were carried out on this portion of the dataset. The QSLRS-CNN model obtained 98.05% training accuracy and 97.13% testing accuracy for 100 epochs. In order to address class imbalance, the model was then trained and tested using several resampling techniques. Based on the findings, the testing accuracy increased from 97.13% to 97.67% overall when SMOT is used. The same methodology was adopted by Abdelghfar et al. [46] for 100 and 200 epochs. The SMOT method shows slightly better performance using 200 learning epochs but takes more time.

Many researchers have utilized CNN-based transfer learning for ArSL recognition. Conducting experiments to compare various pre-trained models was one of the methodologies proposed by several studies [52,54,58,59,62,63,65,66,71,73]. A deep transfer learning-based recognition method for ArSL was proposed by Shahin and Almotairi [52]. They employed several transfer learning techniques, including AlexNet, SqueezeNet, VGGNet16, VGGNet19, GoogleNet, DenseNet, MobileNet, ResNet18, ResNet50, ResNet101, and InceptionV3, based on data augmentation and fine-tuning to lessen overfitting and enhance performance. The experiment results on the ArSL2018 dataset [50,51] show that ResNet101, the suggested residual network system, obtained a maximum accuracy of 99.52%. Alsaadi et al. [58] trained and evaluated four cutting-edge models: AlexNet, VGG16, GoogleNet, and ResNet, using ArSL2018 [50,51], in order to determine which CNN model would be best for classifying sign language. With 94.81% accuracy, AlexNet was found to have the highest outcomes. Next, an AlexNet-based real-time recognition system was built. A comparison analysis based on three popular deep pre-trained models—AlexNet, VGGNet, and GoogleNet/Inception—was conducted [59] using the ArSL2018 dataset [50,51]. Test accuracy varied throughout the models, with VGGNet achieving the best score of 97%. Experiments have been performed by Islam et al. [61] on the ArSL2018 dataset using a variety of pre-trained models, including Xception, VGG16, Resnet50, InceptionV3, MobileNet, and EfficientNetB4. With respect to its relative simplicity, EfficientNetB4 is a heavy-weight architecture. We find that the top model has a 95% testing accuracy and a 98% training accuracy. The architecture of EfficientNetB4 is heavy-weight and relatively intricate. The findings reveal that the EfficientNetB4 model attained the best accuracy over the other models, with 98%.

Using ArSL2018, Baker et al. [65] comprehensively assessed and compared the performance of six different pre-trained models: Xception, ResNet50V2, InceptionV3, VGG16, MobileNetV2, and ResNet152. Early stopping and data augmentation strategies were used experimentally to improve the pre-trained models’ robustness and efficacy. The results demonstrated the greater accuracy attained by InceptionV3 and ResNet50V2, both of which reached 100% accuracy—the best accuracy ever attained. Two pre-trained models with intermediate layers, VGG16 and VGG19, were examined by Nahar et al. [63] to recognize sign language for the Arabic alphabet. Following testing on several datasets and dataset-specific adjustments, both models were trained using various methods. Analyzing the data revealed that the best accuracy results were obtained by fine-tuning these two models’ fifth and fourth blocks. With regard to VGG16, the testing accuracy was specifically 96.51% for the fourth block and 96.50% for the fifth block. Similar findings were observed in the testing accuracy of the second model. Saleh and Issa [54] exploited transfer learning and deep CNN fine-tuning to increase the accuracy of 32-hand gesture recognition by using the ArSL2018 dataset [50,51]. In order to address the imbalance resulting from the difference in class sizes, the dataset was randomly undersampled. There were 25,600 images instead of 54,049 in total. The best accuracy for VGG16 was 99.4%, and for ResNet152, it was 99.57%. Alharthi and Alzhrani [64] carried out a study that is made up of two parts. The first part involves the transfer learning approach by using a variety of pre-trained models, including MobileNet, Xception, Inception, InceptionResNet, DenseNet, and BiT, as well as two vision transformers, ViT and Swin. A number of CNN architectures were trained from scratch in order to be compared with the transfer learning approach in the second part, which used a deep learning approach employing CNNs, the transfer learning method beat other CNN models and achieved stable high performance on the ArSL2018 dataset [50,51]. A comparable high performance of 98% was achieved by ResNet and InceptionResNet.

Ismail et al. [71] suggested a different approach by comparing the performance of single pre-trained models: DenseNet121, VGG16, ResNet50, MobileNetV2, Xception, EfficientB0, NASNetMobile, and InceptionV3, and multi-models: DenseNet121-VGG16 model, ResNet50-MobileNetV2, Xception-EfficientB0, NASNetMobile-InceptionV3, DenseNet121-MobileNetV2, and DenseNet121-ResNet50. It was found that DenseNet121 is the best CNN model for extracting features and classifying Arabic sign language. For multi-models, the DenseNet121-VGG16 multi-model CNN shows the highest accuracy. The study findings reveal that when it comes to ASL feature extraction and classification, multi-models outperform single models. Faster R-CNN based on the pre-trained models VGG16 and ResNet18 was proposed by Alawwad et al. [73]. Using the dataset of self-collected ArSL images, this linkage between the proposed architecture and the ResNet and VGG16 models obtained 93% accuracy.

A few studies have focused their experiments on specific pre-trained models such as EfficientNet [66,74]. Islam et al. [66] presented an innovative approach to Arabic SL recognition that builds feature extraction on a modified version of the EfficientNetB3 model. Using stacked autoencoders, the approach ensures the best possible mapping of input images through powerful feature selection. This approach shows enhanced performance for Arabic sign language after a thorough testing process involving several CNN models. Arabic SL gesture detection becomes simpler and more precise with the addition of densely coupled coding layers, which improves the model’s performance even further. AlKhuraym et al. [74] proposed utilizing a CNN-based lightweight EfficientNet to recognize Arabic sign language (ArSL). A dataset with hand gestures for thirty distinct Arabic alphabets was gathered by numerous signers. Then, the classification outcomes obtained by different versions of lightweight EfficientNet were assessed. With 94% accuracy, the EfficientNet-Lite 0 architecture showed the best results and demonstrated its effectiveness against background variations.

By combining several models rather than just one, ensemble methods seek to increase the accuracy of outcomes in models. The accuracy of the results is considerably increased by the combined models. Two recent studies were found to exploit ensemble methods for alphabet ArSLR [60,62]. Alnuaim et al. [60] proposed a framework that consists of two CNN models, each trained on the ArSL 2018 dataset [50,51]. The two models, ResNet50 and MobileNetV2, were used in conjunction with each other. After using a variety of preprocessing methods, several hyperparameters for each model, and data augmentation strategies, the results reached an accuracy of almost 97% for the entire set of data. The proposed solution by Nahar et al. [62] involves retraining 12 models, namely VGG16, VGG19, ResNet50, InceptionV3, Xception, InceptionResNetV2, MobileNet, DenseNet121, DenseNet169, DenseNet201, NASNetLarge, and NASNetMobile. Once the 12 predictions are obtained, the majority of the predictions will be used by the classification module to increase accuracy. Simple majority voting is a collective method that leverages the majority of the classifiers to determine the prediction, increasing the output’s accuracy. The findings demonstrate that, with a 93.7% accuracy in Arabic language sign classification, the suggested approach outperforms conventional models in terms of speed and accuracy.

RNNs are a type of artificial neural network that is well-suited to capture temporal dependencies and sequential patterns in data. In contrast to feedforward neural networks, which process data in a single pass, RNNs handle data throughout many time steps. This makes RNNs ideal for processing and modeling time series, speech, and text. The most popular RNN architecture is LSTM. LSTM can efficiently capture long-term dependencies in sequential data using the memory cell, which is managed by the input, forget, and output gates. These gates determine what data should be input into, taken out of, and output from the memory cell. Alnahhas et al. [84] proposed an innovative approach that uses LMC and deep learning to recognize dynamic hand gestures that indicate expression in Arabic sign language. In order to process dynamic gestures, the sensory data is first represented as a series of frames, where each frame is made up of values that indicate the hand posture features in that frame. The LSTM model is then used to process the series of frames, which can be used to categorize the series into classes that correspond to different sign language expressions. Using the proposed solution, a system that can identify sign language expressions that can be executed with one or two hands was developed. According to the experiment’s findings, the greatest accuracy was 89% for gestures made with one hand and 96% for gestures made with two hands.

Another type of RNN is BiLSTM, which consists of two LSTM networks, one for forward processing of the input sequence and another for backward processing. The final outcome is then generated by combining the outputs of the two LSTM networks. Some researchers have adopted BiLSTM in ArSLR research. Aly and Aly [77] proposed a methodology to extract the features using a single-layer CSOM rather than depending on the transfer learning of pre-trained deep CNNs. After that, deep BiLSTM—which consists of three BiLSTM layers—was used to recognize the extracted feature vector sequence. BiLSTM includes one fully connected layer and two softmax layers. The proposed approach’s effectiveness was assessed using the Shanableh dataset [75], which comprises 23 distinct terms that were recorded by three separate users. In the signer-independent mode, the evaluation of the proposed solution yielded a high accuracy of 89.5%. Another study carried out by Shanableh [98] has aimed at employing a camera in user-dependent mode for continuous Arabic sign language recognition. The proposed solution is a two-step process wherein the first stage uses deep learning to predict the number of words in a sentence. Next comes a second stage, where a novel method based on motion images and biLSTM layers is used to recognize words in a sentence. The experiments were conducted using one LSTM layer, one biLSTM layer, two biLSTM layers, and three biLSTM layers. According to experimental findings, the suggested method performed exceptionally well when used on a dataset with 40 sentences [96]. BiLSTM with two layers produced the best outcomes, with a word recognition rate of 97.3% and a sentence recognition rate of 92.6%.

Some studies have integrated RNNs with other deep learning architectures, such as CNNs, to benefit from their individual advantages. These hybrid models aim to gather both spatial and temporal information from sign language data, seeking enhanced performance in ArSLR. The baseline study conducted by Luqman and El-Alfy [88] aimed to assess and compare six models based on cutting-edge deep-learning techniques for the spatial and temporal processing of sign videos for ArSL words. These models are CNN-LSTM, Inception-LSTM, Xception-LSTM, ResNet50-LSTM, VGG-16-LSTM, and MobileNet-LSTM. Using both manual and non-manual features, two scenarios are examined for the signer-dependent and signer-independent modes. Color and depth images were used directly in the first scenario, whereas in the second scenario, optical flow was utilized to extract more distinct features from the signs themselves instead of the signers. Using MobileNet-LSTM yielded the best results, with 99.7% and 72.4% for signer-dependent and signer-independent modes, respectively. Ismail et al. [92] proposed fusing different models in order to precisely capture the spatiotemporal change of dynamic word sign language movements and to efficiently gather significant shape information. The models include RNN models, LSTM and gated recurrent unit (GRU) for sequence classification, and deep neural network models that use 2D and 3D CNNs to cover all feature extraction approaches. ResNet50-LSTM was the best multi-model using the same fusion technique among the pre-trained models, including DenseNet121-LSTM, ResNet50-GRU, MobileNet-LSTM, and VGG16-LSTM. Luqman and Alalfy [78] suggested utilizing three distinct models for dynamic sign language recognition based on the combination of two architectures exploiting layers from CNN and LSTM. These models are CNN-LSTM, CNN with two stacked LSTM layers (CNN-SLSTM), and CNN followed by stacked LSTM layers and a fully connected (FC) layer (CNN-SLSTM-FC). After evaluating the models, the results show that CNN-SLSTM outperformed other models in terms of accuracy and training time. Luqman and Alalfy [90] presented a novel approach that includes three deep learning models for isolated sign language recognition: the Dynamic Motion Network (DMN), the Accumulative Motion Network (AMN), and the Sign Recognition Network (SRN). In DMN, different combinations of LSTM and CNN-based models were used to train and extract the spatial and temporal information from the key frame of the sign gesture, and MobileNet-LSTM outperformed all other combinations. The sign motion was encoded into a single image using the Accumulative Video Motion (AVM) technique. AMN was fed this image as its input. Finally, the SRN stream used the fused features from the DMN and AMN streams as input for learning and classifying signals. In 2023, Podder et al. [35] proposed a novel CNN-LSTM-SelfMLP architecture that can recognize Arabic Sign Language words from recorded RGB videos. This study’s dataset comprises both manual and non-manual sign gestures [88,89]. Six distinct CNN-LSTM-SelfMLP architecture models were built using three SelfMLPs and MobileNetV2 and ResNet18 CNN-based backbones, with the purpose of comparing performance in ArSLR. MobileNetV2-LSTM-SelfMLP obtained the highest accuracy of 87.69% for the signer-independent mode. Balaha et al. [94] presented their approach for integrating the CNN and RNN models to recognize isolated Arabic sign language words. Two CNNs were combined, and the output was fed to five cascaded layers of 512 BiLSTM units. To prevent network overfitting, a dropout layer comes after each of these layers. An FC layer with a SoftMax activation function that predicts the output comes after these layers. Using a self-acquired sign language dataset for 20 words, the proposed architecture demonstrated a testing accuracy of 92%.

#### 3.2.5. RQ2.5: Which Evaluation Metrics Were Used to Measure the Performance of ArSLR Algorithms?

Evaluation metrics offer a quantified representation of the performance of the trained model or algorithm. By employing metrics, researchers can evaluate various models and choose which is most effective for their requirements. The evaluation metrics selected are determined by the particular problem domain, the type of data, and the intended outcome. In this section, the most and least used evaluation metrics are presented in all categories of the reviewed ArSLR studies. As illustrated in Table 14, Table 15, Table 16 and Table 17, the most common fundamental metric to evaluate the effectiveness of the proposed ArSL recognition is accuracy, which determines the model’s capability to differentiate ArSL signs correctly and presents the ratio of correctly classified samples across the whole dataset. In continuous ArSLR, researchers report the accuracy in terms of word recognition rate and sentence recognition rate [95,96,98]. The ratio of correctly identified sentences to the total number of sentences in the collection of test sentences is referred to as the sentence recognition rate. When all the words that form a sentence are correctly identified in their original order, the sentence is correctly classified.

Precision, recall, and F1 scores were employed by around half of the fingerspelling ArSLR, as shown in Table 14. The precision of a model is defined as the proportion of true positive predictions among all positive predictions produced by the model. It shows how well the model recognizes positive samples correctly. The proportion of true positive predictions among all actual positive samples is called recall, which is often referred to as sensitivity or true positive rate. It demonstrates how accurately the model represents the positive samples. Precision and recall’s harmonic mean define the F1 score. By taking precision and recall into account, it offers a balanced measure of the model’s performance. The F1 score has a range of 0 to 1, where 0 denotes low performance, and 1 denotes exceptional precision and recall. On the contrary, only one study for miscellaneous ArSL recognition and a few studies for isolated word recognition utilized precision, recall, and the F1 score, as shown in Table 15 and Table 17.

More insight into the model’s performance across several classes is provided by the confusion matrix, which visualizes the results from classifier algorithms. A confusion matrix is a table that compares the number of actual ground truth values of a given class to the number of predicted class values. Less than half of the studies in different categories of ArSLR—except continuous ArSLR—benefited from using this visual representation. 

The loss metric is a measure of the model’s performance in terms of its capability to provide accurate predictions. It shows how the actual outcome, or target value, differs from the model’s predicted outcome. The loss metric is widely used to express the cost or error incurred in making the model’s predictions. The aim is to lower this error by modifying the model’s parameters throughout training. Around one-third of the fingerspelling recognition studies and one-fourth of the isolated word recognition studies used loss as a measure of the model’s effectiveness. Among these studies, the commonly used classification loss was categorical cross-entropy [52,55,58,63,69], whereas LogLoss was used by only one study [85].

Training time was a concern in a few ArSLR studies, with three fingerspelling recognition studies [46,47,55] and one isolated recognition [78]. Along with the training time, testing time was considered in two continuous recognition studies [96,98] and one fingerspelling recognition study [41]. In only one study [97], the response time was measured by calculating the time required to capture and classify the sign in real time for continuous sentence recognition. 

Receiver Operating Characteristic (ROC) curve analysis is a chart that presents a false positive rate (1-specificity) on the X-axis against a true positive rate (sensitivity) on the Y-axis. This metric was utilized by three isolated recognition studies [35,91,93]. The specificity metric is the capacity of the algorithm or model to predict true negatives for each class. A few researchers considered this metric for either fingerspelling recognition [44,52] or isolated recognition [35,95]. The Area Under Curve (AUC) is a metric that is specified by calculating the area under ROC curves, where the AUC should be between 0.5 and 1. It was used by two fingerspelling recognition studies [48,64] and one study for isolated recognition [85]. The Kappa statistic was utilized by two studies for isolated recognition [52,72] to assess the degree of agreement between two sets of multiclass labels. Various evaluation metrics were rarely utilized in the reviewed papers, including Matthews’s Correlation Coefficient (MCC) [52], Root Mean Squared Error (RMSE) [72] and top-5 accuracy [63] for fingerspelling recognition and overlap ratio [76], Jaccard index [91], standard error [35], confidence interval [35], top-1 accuracy [94], G-measure [93], and precision-recall curve analysis [93] for isolated recognition.

#### 3.2.6. RQ2.6: What Are the Performance Results in Terms of Recognition Accuracy?

**Figure 24:**
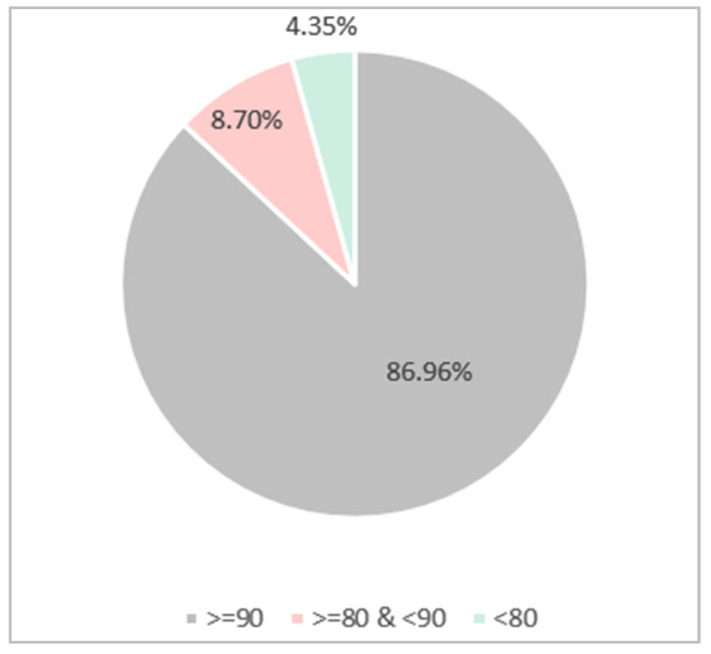
Percentages of Recognition accuracy of the reviewed ArSLR papers.

The majority of research papers emphasize accurately recognizing sign language content, and the main metrics employed in these studies aim to gauge this capacity. Almost all reviewed research papers contain a quantitative assessment of the proposed sign recognition method. The scope and intricacy of testing vary widely, and specific tests are established based on the goals of the research study. Generally speaking, the tests were created to gauge the algorithm’s capability to recognize sign language sentences, words, or alphabets frequently by comparing it with a number of benchmarking techniques. Comparing the performance of various ML/DL models in ArSLR research can be challenging due to the diverse nature of the tests, differences in datasets, evaluation metrics, and experimental setups. Overall, many approaches did rather well and identified over 90% of the signs that were presented, as shown in Figure 24 and Table 14, Table 15, Table 16 and Table 17. Although this typically involved less difficult tasks and was often unsustainable across several datasets, there have been instances where the stated effectiveness was above 97%. One of the most crucial aspects of ArSLR research that can positively affect the proposed solutions’ effectiveness is the optimization of training parameters. The accuracy of 55.57% for a signer-independent isolated recognition [82] was the lowest recognition accuracy achieved among all the reviewed ArSLR studies. Recognition rates of more than 80% are regarded as very strong for continuous ArSLR applications, especially when they are maintained across different datasets; however, the reviewed continuous recognition papers obtained superior results, with all of them above 90%.

One of the important factors that affect the performance results of the proposed ArSLR models is sign dependency. Signer-independent recognition systems are usually tested on different signers than those used for system training; augmenting the signer population benefits these systems. The signer-independent option is more challenging to use than the signer-dependent one, as shown by Table 14, Table 15, Table 16 and Table 17, where, for the identical experimental setting, the performance accuracy of the signer-independent case is consistently lower than that of the signer-dependent case. The reason for the significant decline in recognition accuracy in the signer-independent mode may be traced back to the models that began to overfit the signers during the system learning phase. This has been seen clearly in studies that apply both signer-dependent and signer-independent modes [39,88,90]. The exception was when the input acquisition occurred through wearable sensor devices like gloves [70], where there was no significant drop in the user-independent case. Despite the discomfort and impractical need to wear the glove sensors during the signing, ArSLR systems that utilize this approach achieve a high-performance accuracy of 96% and above [70,95,96].

### 3.3. Challenges, Limitations, and Future Directions in the ArSLR Papers

To answer the third research question, RQ3: What are the challenges, limitations, and future directions mentioned in the reviewed papers? Three research sub-questions have been answered. Table 18, Table 19, Table 20 and Table 21 summarize the papers in each category. Rows shaded in gray indicate methods that utilize wearable sensors.

#### 3.3.1. RQ3.1: Has the Number of Research Papers Regarding ArSLR Been Increasing in the Past Decade?

Figure 6 illustrates the distribution of the 56 papers gathered by the selection process described above, according to the years of publication. From our pool of papers, we can notice an overall pattern of increasing publications over the last ten years, which should be a good indicator of a rising volume of publications in the area in all journals.

#### 3.3.2. RQ3.2: What Are the Limitations and/or Challenges Faced by Researchers in the Field of ArSLR?

The majority of the assessed ArSLR publications omitted information about the constraints or the difficulties they faced in conducting their studies. Approximately 26.79% of the ArSLR studies acknowledged the challenges and limitations that they encountered during their research process. Different aspects were discussed as limitations, including dataset, signers, model performance, training time, and suitability for real-world applications. With regard to the dataset, the small size was considered a limitation by Latif et al. [55] for alphabet recognition and Almasre and Al-Nuaim [81] for isolated word recognition. Abdelghfar et al. [46,47] utilized a limited set of images of static gestures that show the discontinuous letters at the beginning of the Qur’anic Surahs. Alharthi and Alzahrani [64] pointed out that the dataset was not representative enough for alphabet recognition. Fadhil et al. [63] used a dataset of similar fingerspelling sign language images. Extra data and low-quality blurred frames in the captured signs stored in the dataset were reported by Bencherif et al. [39]. The enormous number of produced frames in the dataset, particularly when sign gestures are captured at high frame rates, was a challenge pointed out by Luqman [90]. Limitations in the process of video capturing were discussed by Bencherif et al. [39], including relying on the original suboptimal factory parameters for the two Kinect cameras, capturing at a low frame rate, and including a large field of view of each camera. Signers were another aspect mentioned in a number of studies; for example, Latif et al. [55] reported the limitation in the number of signers who volunteered to perform alphabet signs. Luqman [90] stated that word signs in the dataset were performed by nonexpert signers who incorporated non-sign language gestures into the sign language. Moreover, the various signers who perform the same signs show differences in their gestures. A few signers were recruited to perform the word signs stored in the dataset used by Podder et al. [35]. Bencherif et al. [39] reported that some singers had trouble coordinating their hands and making the same gestures. A few researchers highlighted the limitation in real-time recognition of alphabet signs [41,55,64] and continuous sentences [98] for real-world applications due to the required computation time. Agab and Chelali [41] pointed out that accurate segmentation is necessary when using their model in practical, real-life applications. Model training time was another limitation that impacted fingerspelling sign recognition, as mentioned by Shahin and Almotairi [52] and Fadil et al. [63]. The limitation in system hardware, including processing and memory requirements for alphabet sign language recognition, was discussed by Latif et al. [55]. Some researchers admit the limitations in their proposed models; for example, the model proposed by Alsaadi et al. [58] is limited to detecting only one object (a hand) without taking the background into consideration, which would affect the performance. The detection process in their proposed model is highly sensitive to variations in the hand’s pose. In addition to the limitation in model robustness to illumination reported by Agab and Chelali [41], the HOG descriptor efficiently captures the hand structure only if there is no background clutter. The semantic-oriented post-processing module suggested by Badawy et al. [80] to detect and correct any translation errors would perform well in a particular predefined field.

#### 3.3.3. RQ3.3: What Are the Future Directions for ArSLR Research?

**Figure 25:**
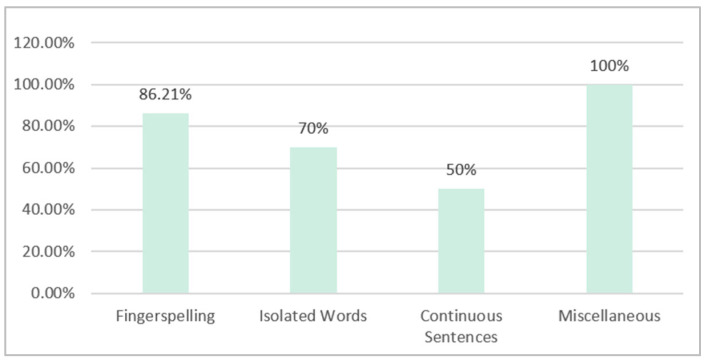
Percentages of papers that discuss future work in each category.

Many of the reviewed publications, with a percentage of 78.57%, exhibited future work for their proposed solutions. This percentage is distributed among the different categories of ArSLR studies, as illustrated in Figure 25. Different dimensions for future work have been discussed, including datasets, data acquisition devices, data preprocessing and segmentation, feature extraction, recognition models, expanding to other ArSLR categories, and developing practical real-time systems.

In the category of finger spelling recognition, some researchers discussed increasing the dataset size in their future plans [43,44,55,56,64]. Other researchers planned to utilize different and various datasets to test and evaluate their proposed solutions [46,47,57,58,62,68]. Publishing the self-acquired dataset was pointed out by Kamruzzaman [69]. Building video-based was a concern for Ismail et al. [71]. In terms of data acquisition devices, Kamruzzaman [69] planned to consider more advanced hand gesture-recognizing devices such as Leap Motion or Xbox Kinect. In data preprocessing, implementing different data augmentation techniques was a concern for a few researchers [58,64]. With regard to feature extraction, Alzohairi et al. [49] discussed the potential improvement of the model by assigning a relevant feature weight to each sign gesture. Investigating the impact of normalization and whitening on feature extraction was a concern for Hasasneh [44].

The majority of the studies have focused on recognition models and how to boost their performance as one of the future directions. For example, some researchers have focused on improving the accuracy of results in cases of image occlusion [43] or complicated backgrounds [41]. Decreasing the learning parameters and model size using quantization or model-pronening techniques was proposed by Islam et al. [66]. Shahin and Almotairi [52] discussed a possible reduction of the training time by implementing a low-depth residual network. Hasasneh [44] suggested investigating the sparsity factor with various models’ parameters. A number of researchers have mentioned specific recognition models to be investigated in their future studies, such as kernal SVM [49], RNN [57], YOLO algorithm [73], and RNN together with LSTM [46,47]. The interest has been shifted toward transfer learning by many researchers [46,47,58,61,63], where it was first mentioned as a future direction in 2021 by Alani and Cosma [57]. Zakariah et al. [61] suggested combining different transfer learning models for single-hand gesture recognition, such as MobileNet and ResNet50 architectures, and applying these models to recognize the two-hand gestures. Fadhil et al. [63] emphasized the necessity of further understanding the decision-making processes of the transfer learning model’s layers, such as ResNet. It was also suggested that future research would extend the current proposed approaches to be capable of recognizing words and sentences [48,55,64,71,74].

Other studies pointed out the need to enhance the proposed systems for real-time acquisition and recognition [41,43,68]. One potential avenue for future improvement is the development of real-time mobile applications for ArSLR [55,56,58,71]. Tharwat et al. [74] recommended creating educational materials for deaf and dumb children, while Shahin and Almotairi [52] proposed creating an entirely automated ArSLR system. Using deep learning models, Abdelghfar et al. [46,47] and Tharwat et al. [72] suggested translating the meanings of the Holy Qur’an into sign language.

**In the category of isolated word recognition**, some researchers suggested expanding the dataset by increasing the number of signs [33,70,91,94], observations [85], and signers [94]. Exploring how the proposed methods can be applied to different datasets was in the plans for a number of studies [78,88,91,94]. Podder et al. [35] pointed out that the work would be improved by building a larger sign dataset of alphabets, numbers, words, and sentences from various signers, as well as variations in background, lighting, and camera angles. With regard to data acquisition devices, Almasre and Al-Nuaim [81] emphasized the need for employing faster Kinect and LMC to collect and recognize gestures more accurately and instantly. Deiche et al. [83] suggested examining various scenarios in which LMCs could be combined with other sensors to improve the overall performance. Glove design was a concern for Qaroush et al. [70], where Bluetooth IMU sensors can be utilized, together with 3D printed rings that allow the sensors to be positioned on the fingers.

In the preprocessing stage, Qaroush et al. [70] suggested employing more sophisticated methods such as sensor fusion (e.g., Kalman filter). In terms of feature extraction, Ahmed et al. [33] planned to increase the number of non-manual features for full ArSL recognition, whereas Hisham and Hamouda [37] suggested incorporating more feature engineering. Qaroush et al. [70] mentioned merging magnetometer data with ACC and GYRO features to detect magnetic north.

Many of the studies have paid attention to recognition models and improving their accuracy. Some of the researchers suggested incorporating efficient techniques to decrease the processing complexity and increase accuracy rates, including deep learning algorithms [78,83,85,88,93,94]. Qaroush et al. [70] suggested utilizing sequence-based classification techniques such as HMM and RNN. To improve the SL recognition performance, a combination of DL models can be developed [33,91]. Luqman [90] mentioned that transformers and the attention mechanism are two other models that can be utilized to recognize sign language. Podder et al. [35] suggested creating a sign language transformer that uses MediaPipe Holistic’s landmark data rather than videos as input, incorporating more cutting-edge 1D and 2D CNNs into a real-time Arabic Sign Language Transformer and including the attention mechanism in the model for Arabic sign video classification so that the model can be trained on bigger datasets and be capable of recognizing sign videos in real-life scenarios.

A number of studies showed that further work is needed to extend the proposed approaches to incorporate continuous sign language recognition [33,78,83,88,94] and to provide a mechanism that can transform sign language movements into complete sentences while recognizing overlapped gestures [84]. Almasre and Al-Nuaim [81] highlighted their intention to make use of depth sensors and supervised machine learning to identify ArSL phrases while considering the LMC’s constrained workspace and the user’s motion. Marzouk et al. [91] underlined the importance of expanding the proposed model to recognize sign boards in real-time applications. Developing an upgrade plan for the system to enable mobile platform deployment was pointed out by Deriche et al. [83]. Badawy et al. [80] stated that the accuracy of the translation can be improved by including a semantic-oriented post-processing module to identify and fix any translation errors.

**In the category of continuous sentence recognition**, Hisham and Hamouda [97] recommended conducting further testing of more words from various domains. They also suggested improving the segmentation method and utilizing alternative techniques in the recognition phase, such as DTW, for the purpose of increasing accuracy and improving the proposed system. Shanableh [98] emphasized the importance of making the proposed solution appropriate for real-time sign language recognition and minimizing the computation time.

**In the category of miscellaneous recognition**, Hisham and Hamouda [37] suggested incorporating more feature engineering, working on large samples of complete sentences, and utilizing deep learning to enhance recognition accuracy. Bencherif et al. [39] pointed out the need to add a sign boundary detector or a network to the proposed solution in order to determine whether a shape is a sign or a transitory gesture. They stressed that more research should be done to find the smallest group of unique frames and/or key points that show a sign by putting the produced frame key points into meaningful reduced clusters. This would make the deep learning model smaller and lighter for mobile devices. They also highlighted the significance of minimizing network size and maximizing classification performance by improving delay removal across the pipeline using convolution suppression and optimum data propagation. Additional enhancements, including the ability to zoom in on singers and the addition of an automated method for detecting palm positions, were also suggested by Bencherif et al. [39]. Elsayed and Fathy [38] underlined the necessity of improving deep learning with ontology to address dynamic real video in real-time applications and transforming the system into a mobile application.

### 3.4. Discussion, Future Perspectives and Limitations

Our analysis of the relevant literature indicates that vision-based recognition algorithms for alphabet, isolated words, and sentences for ArSL recognition have been successfully applied by researchers in this field. It is noteworthy to mention that I did not find any study that used a sensor-based technique for fingerspelling ArSL recognition. Despite their performance being promising, limited sensor-based studies were found for isolated words and continuous recognition. Research on ArSLR has recently focused more on vision-based solutions because they offer minimal to no constraints on users, unlike sensor-based approaches. Despite being non-invasive, vision-based techniques are limited by the low-quality images of traditional cameras. One more issue is that simple hand features can lead to ambiguity, while more complex features need more processing time. As both vision-based and sensor-based approaches have the potential to attain the best performance in this field, this shows the feasibility of research efforts utilizing both of them.

This section discusses the results found in the analyzed papers and is based on the answers to the research questions.

#### 3.4.1. Dataset Characteristics

The ArSLR process relies heavily on the dataset to train and test models. Therefore, when selecting a dataset, several requirements should be considered, including:

Availability: Whether the dataset is publicly available or not.

Diversity: To ensure that models are capable of handling real-world circumstances, datasets need to be diverse. Thus, the datasets should incorporate a variety of backgrounds, lighting situations, camera angles, and representations of signs by different signers.

Size: The size of the dataset affects model performance. Therefore, larger datasets are generally better for improving model accuracy.

Sign representation: Different representations of signs are available in the dataset in the form of different modalities, including RGB, depth, skeleton joint points, or others.

Data quality: Performance may suffer from a large amount of poor-quality data. Therefore, data should be high-resolution and free of watermarks.

Annotation quality: Any dataset should include accurate, comprehensive, and consistent annotations for key points and sign detection.

The fact that the ArSLR datasets available today only partially meet these requirements is disappointing and could negatively impact the performance of the model. This systematic review shows that a few datasets are publicly available. However, publicly available datasets are important in creating benchmark datasets to compare the performance of different algorithms proposed in previous studies on ArSLR. The lack of available datasets is one of the challenges impeding research and improvement in Arabic sign language recognition. This is mainly caused by a shortage of experienced ArSL specialists as well as the time and cost involved in gathering sign language data. Furthermore, researchers might have trouble acquiring reliable ArSL datasets since Arabic is a complicated language by nature. It could be challenging to directly compare the recognition accuracy of the various approaches because some studies created their own data, which is typically private or unavailable to other researchers.

One of the variables that affects the diversity of the ArSL dataset is the total number of signers. This aspect is crucial for assessing the generalization of the recognition systems. The majority of the reviewed ArSL datasets were built with a relatively small number of signers and only a few classes, which calls into doubt their representative value. Signer-independent recognition systems that are tested on signers other than those who participated in the system training benefit from having more signers. Thus, when faced with slightly varying presentations of sign language gestures, the performance of any ML/DL model that relies on those datasets may be jeopardized.

An additional consideration in evaluating a sign language dataset is the number of samples. Training ML/DL models requires numerous samples per sign with certain variances per sample. Data on sign representation is very crucial for assessing datasets. Most of the reviewed Arabic sign language datasets are available in RGB format. However, a number of the datasets capture the signs using multimodality devices such as Microsoft Kinect and LMC, which provide additional representations of the sign sample, like joint points and depth. A few datasets rely on wearable devices, such as DG5-VHand data gloves and 3-D IMU sensors, to record the required features of the hand gestures. Multi-modal datasets are becoming more prevalent, especially in the categories of isolated words and continuous sentence recognition. This is a positive indicator showing the next phase of ArSLR research and providing more opportunities for creative thinking.

One of the most important factors that might influence the rate of advancement in any branch of AI research is the availability of high-quality datasets for model training and testing. As a relatively new field of interest, ArSLR research experienced this issue at first, but the reviewed papers show that situations are beginning to improve in this matter.

Owing to these issues with current datasets, some researchers tend to integrate two or more datasets while training their models. The goal of combining datasets is to overcome the shortcomings of each one individually. Despite the features of these datasets, data augmentation remains required to enhance data diversity. Therefore, for ArSLR tasks, an exhaustive dataset with a variety of data that tackles the issue of occlusions and enables accurate labeling is still essential.

ArSLR research shows considerable promise for enhancing accessibility for the deaf and hard-of-hearing groups; however, it raises a number of significant ethical and privacy issues. These issues mostly focus on informed consent, data privacy, and security. The ethical and privacy considerations that researchers must carefully consider when gathering and utilizing data for ArSLR are discussed below.

A key ethical principle in research is informed consent, which guarantees that study participants are aware of the goals, methods, risks, and intended use of their data. Participants must be clearly informed about the following when conducting ArSLR research:

- How their sign language gestures will be captured and examined.
- What data will be gathered, such as motion data or video footage?
- The data’s intended use, such as sharing it with third parties or using it to train models, and who will have access to it.
- The possible risks include invasions of privacy or improper use of personal information.

Data anonymization is essential for protecting the privacy and identity of research participants. Researchers must ensure that identifiable features like faces, clothing, or locations are eliminated or obscured when using motion data or video footage to train ArSLR systems. They also need to ensure that data linkage is avoided, which means that individual participants cannot be identified through combined data from multiple sources or over time. In video-based data collection, this becomes especially difficult because identifying people by their body language may unintentionally lead to identification. In addition to ensuring that all identifying features of the data are anonymized before usage, researchers should investigate methods like face-blurring.

The storage and protection of data must also be carefully considered in ethical research. Researchers must ensure that data is stored securely, with only authorized individuals having access; data is stored in accordance with relevant data protection laws, ethical standards, and regulations; and participants’ rights to request removal of data and withdrawal of consent are upheld.

#### 3.4.2. ArSLR Methodologies and Techniques

In Section 3.2, a number of elements that constitute the ArSLR methodologies and techniques were discussed. One of these elements is data preprocessing, which mainly prepares the data for the next ArSLR phases, and therefore choosing suitable preprocessing techniques would affect the performance of the model. Various techniques were adopted by the reviewed studies, including color space conversion, resizing and cropping, normalization, data augmentation, and noise reduction. Utilizing these techniques rely on different factors, such as

- The quality of acquisition devices: These devices usually produce low-quality data, which may lead to decreased accuracy.
- The calibration of acquisition devices: Some researchers do not calibrate their devices to the appropriate parameters and work with default settings, which are not ideal in most situations.
- Improper distance between the signers and the acquisition devices: The distance should be adjusted to be adequate. The performance may be affected by how close or how far away the signer is from the acquisition device.
- The background, illumination, and surroundings all have a significant impact on the way the dataset is prepared.
- Small datasets: To improve the model’s performance, augmentation techniques could be applied to increase the amount of training data.

The results from the analyzed papers show high popularity of data augmentation recently, especially in the category of fingerspelling recognition, where the data tend to be static images. A number of recent research cases show how transfer learning and data augmentation techniques help overcome dataset scarcity issues and reach recognition accuracy of 100% [65,66,92]. 

The primary focus of vision-based ArSLR is typically hand segmentation, with non-manual segmentation for facial expressions and body gestures coming in after. Segmentation is accomplished by using a variety of segmentation approaches, such as neural network-based segmentation, region-based segmentation, thresholding, edge detection, and clustering. The findings reveal that since 2020, the segmentation has been shifted towards CNN-based algorithms and transfer learning, including VGG, AlexNet, MobileNet, Inception, ResNet, Densnet, SqueezeNet, EfficientNet, CapsNet, and others. A few researchers adopted pretrained vision transformers, such as ViT and Swin, and semantic segmentation DeepLabv3C, where they proved their contribution to enhancing the accuracy of the model.

Feature extraction is a crucial step in the ArSLR process. Thus, the feature vectors obtained from this process serve as the classifier’s intake. The feature extraction approach should identify structures robustly and consistently, irrespective of changes in the brightness, location, size, and orientation of the item in an image or video. The findings show that some ArSLR studies tend to adopt hybrid feature extraction techniques to address the shortcomings in any individual technique and take advantage of their benefits. Other researchers have recently used popular deep learning methods, such as CNNs, to extract relevant features. These methods take features from the first layers and input them into the ones that come after. CNNs and LSTM have been used together by some researchers to extract temporal and spatial data, which are useful in isolated words and continuous sentence recognition. Pretrained models have been used to extract features in the majority of studies on fingerspelling ArSLR that have been conducted since 2019.

Section 3.2 reveals that deep learning algorithms, such as RNNs and CNNs, have emerged as powerful tools in ArSLR research and have seen widespread use since 2019. However, despite their advances, they also encounter a number of challenges that should be overcome in order to fully exploit their effect and applicability in ArSLR research. The scarcity of longitudinal datasets presents one of the problems RNNs encounter. RNNs excel at modeling temporal dependencies and capturing sequential patterns, making them ideal for recognizing continuous sentences. However, collecting diverse large-scale longitudinal datasets is the key to training resilient RNN models. Furthermore, RNNs are challenged by the wide range of sign language data. CNNs have proven to be remarkably effective at recognizing ArSL. They do, however, have challenges in the field of ArSLR research. The requirement for diverse and large-volume datasets for the effective training of CNN models is one of these challenges. ArSLR data may demonstrate class imbalances and tend to be small in size; therefore, careful data augmentation techniques are needed to overcome these problems. Moreover, CNNs struggle with generalizing across different signers and acquisition modalities. Developing robust techniques to handle these challenges and ensure model generalization is a key area of research. For this reason, a method called transfer learning—in which the model is trained on a large training set—has been suggested as a remedy. The results of this training are then treated as the starting point for the target task. Transfer learning using pretrained models has been successful in the field of ArSLR. Frequently used pretrained deep learning models include common models such as AlexNet, SqueezeNet, VGGNet16, VGGNet19, GoogleNet, DenseNet, MobileNet, Xception, Inception, and ResNet, which are all typically utilized for image classification. In an attempt to improve model accuracy, ensemble approaches—which incorporate different models—have also been used recently. The integrated models significantly improve the results’ accuracy. Additionally, some researchers have integrated RNNs with other deep learning architectures, such as CNNs for isolated words and continuous sentence recognition. These hybrid models aim to gather both spatial and temporal information from sign language data, seeking enhanced performance in ArSLR. A few more recent studies have boosted the performance by adopting vision transformers and attention mechanisms.

The capacity of the proposed models to accurately accomplish the main task—that is, to recognize or translate sign language—is how their performance is often evaluated. The primary metric to evaluate the effectiveness of the model is the average accuracy over the whole dataset; a greater percentage denotes a more accurate approach. It can be difficult to compare the effectiveness of different ML/DL models in ArSLR research because of the variety of tests, differences in datasets, evaluation metrics, and experimental setups. Overall, many approaches did rather well and identified over 90% of the Arabic signs that were displayed. The fact that many of the studies adopt signer-dependent mode testing contributes to achieving such high accuracy. Although the ML/DL model’s capacity is typically limited to the signs learned from the training set, it is possible to accomplish some generalization with regard to other individuals exhibiting the same sign. Thus, one of the most crucial aspects of SLR research is the optimization of training parameters, which can significantly affect the effectiveness of the proposed solutions. More sophisticated systems seek to comprehend increasingly complicated continuous sign language speech segments and to enhance their real-time recognition capabilities. These applications are far more complicated than simple word or letter recognition and often require combining the analysis of various signs to decipher a particular sequence’s meaning. In order to capture semantic nuances and prevent comparable signs from being confused, researchers have to use hybrid architectures and advanced sequence-to-sequence models.

The relationship between computational resources and model complexity is crucial in ASLR, particularly as the field moves toward the use of deeper and more complex neural networks. Earlier research on ArSL recognition may have relied on less complex models like shallow neural networks, decision trees, or support vector machines. The accuracy of these models is often lower, particularly in more complicated sign sequences or gestures, but they may not reflect the subtleties or contextual dynamics of sign language. More recently, deeper neural networks are being investigated to enhance performance, particularly for dynamic and continuous sign gesture recognition. For instance, RNNs or transformers for sequential sign interpretation and CNNs for spatial feature extraction (from images or videos). These models demand considerably more memory, processing power, and training time than other models. ArSLR systems frequently need to process a variety of input data types, such as images, video, depth sensors, or motion capture data. This boosts model complexity and calls for more advanced networks.

The complexity of the model has a substantial impact on the computational resources required for both training and deployment. For efficient training and inference, deeper networks with more parameters require more processing power, such as high-performance GPUs or TPUs. In order to maintain large datasets and model weights, complex models demand a substantial amount of memory and storage capacity. Deeper network training also necessitates processing massive amounts of video data, frequently in real-time, which can be computationally costly. Longer training times for more complicated models could result in increased expenses and consumption of power. This is especially problematic when scaling up to large datasets for ArSLR or in circumstances with restricted resources.

There is a trade-off between the model’s performance on ArSLR tasks and its complexity. Deeper models typically offer higher accuracy, but they also come with a higher processing cost. Model performance and efficiency must be balanced, particularly in real-time applications where speed is critical. Researchers are investigating methods like transfer learning, quantization, and model pruning to lessen the computational load without appreciably compromising performance.

Both the Arabic language and sign languages in general have inherent complexities that contribute to the difficulty of handling contextual interactions and advanced semantic analysis in ArSLR. Like other sign languages, ArSL mostly depends on the surrounding signs and contextual cues, such as body posture, facial expressions, and hand shape, to accurately express meaning. A sign’s meaning might change depending on the context. For instance, depending on its position, speed, and the facial expressions that accompany it, a single sign may have several meanings. Several signs in continuous sign language have similar hand shapes or movements but differ slightly (e.g., by speed, direction, or facial expressions), and gestures also frequently overlap. It might be challenging to recognize these subtle variations. Parsing the semantic structure of ArSL, or comprehending the meaning behind a sequence of signs, remains a challenging task. This includes contextual factors such as the link between signs, pronominal reference, negations, and non-manual markers. The lack of comprehensive ArSL datasets restricts the ability to train robust models, particularly for continuous signing.

A comparison of the most popular ML/DL techniques in ArSLR research, such as SVM, KNN, HMM, RF, MKNN, NB, CNNs, RNNs, hybrid CNN-RNN, transfer learning, and transformer-based techniques, is provided in Table 22 in terms of recognition accuracy, efficiency, and robustness. This comparison has been derived from typical patterns observed in the reviewed ArSLR papers. The performance can differ considerably depending on the dataset, preprocessing and segmentation methods, feature extraction techniques, and the specific recognition task (static vs. dynamic signs) used.

#### 3.4.3. Comparison with SOTA Systems Used in Related Languages

In this section, a comparison of ArSLR research with state-of-the-art (SOTA) systems used in larger and well-established corpora like Ankara University Turkish Sign Language Dataset (AUTSL) [103], Word-Level American Sign Language Dataset (WLASL) [104], BBC-Oxford British Sign Language Dataset (BOBSL) [105], RWTH-PHOENIX-Weather 2014T [106], and Microsoft American Sign Language (MS-ASL) [107] is provided. The aspects for this comparison include dataset size and diversity, modeling techniques, multimodal data integration, performance metrics and benchmarks, and scalability and global relevance.

- Dataset Size and Diversity

ArSLR datasets are generally small, with limited vocabularies (e.g., specific letters or isolated signs) and minimal variation in signer identity, environment, or recording settings, often featuring fewer than 100 signs and scarce continuous signing samples. In contrast, SOTA systems utilize much larger and more diverse datasets. For example, AUTSL includes 226 signs with multiple signers, capturing both isolated and continuous gestures with style and context variations. WLASL offers over 2000 signs recorded across varied settings and multiple signers, making it one of the largest for isolated word recognition. BOBSL emphasizes conversational British Sign Language with continuous signing and a large vocabulary, while RWTH-PHOENIX-Weather 2014T provides a highly structured corpus for German Sign Language (DGS) with subtitles for context alignment. MS-ASL features 1000+ signs recorded in diverse environments with signer variability, enabling robust testing. To achieve global SOTA, ArSLR datasets must grow in size, vocabulary, signer diversity, and recording conditions.

- Modeling Techniques

ArSLR modeling techniques primarily rely on CNNs for spatial feature extraction and hybrid CNN-LSTM models for temporal learning in dynamic signing tasks, with limited adoption of advanced frameworks such as graph-based models, transformers, or multimodal attention mechanisms. On the contrary, SOTA systems employ more sophisticated approaches. AUTSL incorporates models like Spatio-Temporal Features with LSTM (STF + LSTM) for spatio-temporal features [108], 3D-DCNN for video-based learning, and Spatio-Temporal Multi-Graph Convolutional Neural Network (ST-MGCN) for skeleton-based recognition [109]. WLASL leverages graph-based neural networks, including Spatial-Temporal Graph Convolutional Networks (ST-GCN) [110] and transformer architectures for robust spatio-temporal learning [111]. BOBSL and RWTH-PHOENIX utilize sequence-to-sequence models, attention mechanisms, and transformers for continuous signing recognition [112,113,114]. MS-ASL explores multimodal architectures and pre-trained deep learning models, such as I3D, ResNet, and transformers, for both isolated and continuous tasks [110,115]. To advance, ArSLR research must adopt SOTA techniques like transformers, Graph Convolutional Neural Networks (GCNs), and multimodal networks to enhance spatio-temporal and contextual learning.

- Multimodal Data Integration

ArSLR systems primarily rely on RGB video data, with limited exploration of additional modalities such as depth or skeletal representations. In contrast, SOTA systems incorporate multimodal data for enhanced performance. AUTSL combines RGB, depth, and skeletal data for comprehensive feature extraction. WLASL and MS-ASL leverage skeletal data for graph-based learning to enrich gesture representation. RWTH-PHOENIX aligns subtitles with continuous signing to provide contextual disambiguation, and BOBSL utilizes both contextual and visual cues to improve accuracy in conversational signing. Expanding ArSLR systems to integrate depth, skeletal, and contextual data can significantly enhance their robustness and versatility.

- Performance Metrics and Benchmarks

ArSLR systems often achieve high performance with accuracy above 90% in signer-dependent recognition but struggle with signer-independent tasks due to limited dataset variability and lack of standardized evaluation benchmarks for cross-dataset comparisons. On the contrary, SOTA systems excel in signer-independent recognition. WLASL and MS-ASL achieve accuracy above 90% by utilizing diverse datasets and robust models. RWTH-PHOENIX sets standardized benchmarks for continuous signing, enabling models to achieve state-of-the-art accuracy. AUTSL consistently delivers high accuracy—above 95%—for both isolated and continuous recognition tasks. To align with global standards, ArSLR research must develop signer-independent benchmarks and test systems on more diverse datasets.

- Scalability and global relevance

ArSLR research faces scalability challenges due to small datasets and the limited adoption of advanced modeling techniques, making it less prominent in global SLR research and reducing participation in shared tasks or standardized benchmarks. In contrast, SOTA systems leverage larger datasets and advanced models, enabling scalability across languages and domains, facilitating cross-dataset evaluations, and supporting real-world applications. Expanding datasets and adopting transferable frameworks can enhance the scalability of ArSLR systems and make the research more globally competitive.

In conclusion, ArSLR research has made progress but lags behind SOTA systems like AUTSL, WLASL, BOBSL, RWTH-PHOENIX, and MS-ASL in terms of dataset diversity, modeling sophistication, multimodal integration, and benchmarking. By adopting advanced models, expanding datasets, and standardizing evaluations, ArSLR can align with global standards and improve scalability for real-world applications.

#### 3.4.4. Future Perspectives

Despite significant progress in recognizing Arabic sign language, existing deep learning models still face challenges with accuracy and efficiency. In this section, several potential ideas for future directions in ArSLR are proposed and discussed.

- The difficulties posed by the scarcity of high-quality publicly available datasets in ArSLR research must be addressed. Deep learning algorithms typically require a lot of labeled data for optimal results. However, the complexity and expense of obtaining data usually result in limited ArSLR datasets. Transfer learning approaches, which fine-tune pretrained models on relevant tasks or datasets for ArSLR analysis, can help researchers overcome this problem. Designing effective transfer learning models for ArSLR involves several strategies, each aimed at enhancing the model’s generalization capabilities and accuracy, especially when dealing with the limited availability of labeled data in ArSL. It is feasible to implement transfer learning from pre-trained models on large, diverse datasets, such as ASL (American Sign Language) or other gesture-based datasets. Even in underrepresented languages, cross-lingual transfer learning can improve generalization and save the time and effort needed to create an ArSL-specific model from the ground up. Moreover, the model can be fine-tuned using a more specific but smaller ArSL dataset. This requires gathering a comprehensive dataset of Arabic signs and using techniques such as data augmentation to increase the available volume and diversity of the available data, creating more resilient and generalizable models. Multiple input modalities, such as depth, skeletal joint data, and RGB video frames, may also be combined. In real-world situations where sign language may change based on the context, signer, and surroundings, multi-modal learning can assist in capturing various elements of sign language, such as motion, hand shape, and spatial context, and result in improved recognition accuracy and robustness. Transfer learning models can be designed to extract fine-grained features specific to Arabic sign language, such as local variations in hand gestures, facial expressions, and body language, to improve performance in recognizing subtle differences in signs. This can entail focusing on the most significant features for ArSL recognition by training attention-based models or specialized CNNs.
- Building high-quality, large-scale, and diverse ArSL datasets is another avenue for research that could lead to more progress in the field and enable direct comparison of the recognition accuracies of the various methodologies. Although situations continue to improve, it is still challenging to test more sophisticated applications that demand large vocabulary sizes in order to fully utilize current or upcoming techniques. Building high-quality ArSL datasets requires a well-structured, systematic approach to ensure both accuracy and diversity. A wide range of participants from different backgrounds, ages, and genders who are fluent in ArSL or have experience with it should be involved in the data gathering process. Clear motion capture requires the use of high-resolution cameras (1080p or higher) and a steady frame rate (e.g., 30 fps) to maintain the subtle details of hand shapes and movements. To increase robustness and generalizability, recordings should be made in a variety of settings, including different lighting and backgrounds. In order to record dynamic gestures, facial expressions, and hand configurations in different environments, researchers can use a variety of data collection techniques, including video recordings and real-time capture with depth sensors, like Kinect and LMCs. For consistent labeling, researchers may also employ a team of experienced annotators who are fluent in ArSL or create a comprehensive set of standardized labels for signs that consider variations in handshape, movement, orientation, facial expressions, and contextual usage. A broad variety of signs, such as numbers, common phrases, and simple words, would also be covered by the dataset. To reduce noise, enhance clarity, and normalize illumination, the gathered data must be preprocessed. Researchers can assure data privacy by obtaining participants’ consent for data usage and anonymizing personal identifiers. ArSLR researchers may use open data formats such as the sign language corpus framework [116] to facilitate data sharing and integration. Furthermore, it is feasible to establish a feedback system whereby ArSL users evaluate early dataset versions for comprehensiveness and accuracy. The dataset can be enhanced using iterative refinement procedures in response to user feedback.
- Although significant progress has been made in fingerspelling and isolated ArSLR, where algorithms only need to recognize a single letter sign or word, continuous ArSLR, which interprets longer speech segments, has not seen the same level of progress. This task cannot be simplified to the recognition of individual gestures because contextual interactions among signs have a significant effect on the interpretation of the sentences. Current efforts to create continuous ArSLR capabilities have shown very limited success and often produce errors when advanced analysis of semantic nuances is necessary. This is certainly one of the hottest topics in ArSLR research, and it will keep being investigated in a variety of ways in an effort to find a configuration that may solve the issues impeding the development of extremely powerful tools. Considering current findings, I anticipate that future research in this particular area will focus on context-aware models that use sequence-based analysis, such as transformers or RNNs, to capture the sequential nature of signs and their contextual relationships. The immediate context (prior or subsequent signs) and the global context (the overall sentence structure, non-manual signs like facial expressions, etc.) should both be considered by these models. More accurate interpretations of ambiguous signs could be made possible by using attention mechanisms to emphasize the most contextually relevant portions of a sign sequence. ArSLR models can also be improved by integrating multimodal data, such as body posture, face expression, and hand gesture recognition. By processing multiple inputs concurrently, multimodal deep learning frameworks—like multimodal transformers—may enhance the model’s comprehension of the semantic information that is communicated by combining these inputs.
- Deep learning models for ArSLR can evolve by integrating advanced frameworks from state-of-the-art research, as demonstrated in datasets like AUTSL [103]. These frameworks emphasize the fusion of spatial and temporal features, multimodal data processing, and cutting-edge model architectures. Here is how these approaches can be adapted for ArSLR:
  - Frameworks like STF + LSTM [108] and Feature Engineering with LSTM (FE + LSTM) [117] demonstrate how the system can model sequential dependencies by combining spatial information, such as hand shapes and locations, with temporal dynamics, like movement trajectories, using LSTM networks. Continuous signing may be processed efficiently by such models, which can also handle variations in the speed and execution of gestures and extract distinctive spatial features from ArSL gestures (e.g., finger positions for specific letters).
  - 3D Convolutional Models for Video Input including 3D-DCNN [109] and MViT-SLR [118]: These networks are highly suited to sign language video data since they can capture motion and depth. Hierarchical feature scaling transformers can also be applied for temporal and spatial learning using Multiscale Vision Transformers for Sign Language Recognition (MViT-SLR). Using 3D convolutions to learn hand gestures and combining them with MViTs for hierarchical temporal modeling could be one way to adapt to ArSLR.
  - Graph-Based Models for Skeleton Dynamics (e.g., ST-MGCN [109], HW-GAT [119]): These models use graph convolutional networks (GCNs) to model relationships between skeletal keypoints and temporal dynamics. ST-MGCNs are used to model complex joint movements over time, and Hand-Weighted Graph Attention Networks (HW-GAT) are used to assign higher weights to critical joints like fingers in hand-dominated gestures. Adaptability to ArSLR comprises applying ST-MGCNs or HW-GAT for fine-grained recognition of Arabic sign trajectories and leveraging skeletal data, such as OpenPose or MediaPipe, to track hand and body keypoints.
  - Transformer-Based Architectures, such as Video Transformer Networks with Progressive Filtering (VTN-PF) [120], can be applied for global temporal modeling, progressively refining key gesture features. These architectures can be adapted to ArSLR to process Arabic sign videos with high variability in signer style and environmental conditions. Moreover, progressive filtering can be employed to emphasize critical frames, such as key transitions in signs.
  - Multimodal integration: Advanced frameworks can integrate video, depth, and skeletal data inputs for a richer feature representation. Sign Attention Module (SAM-SLR) [121] integrates spatial, temporal, and modality-specific features using attention mechanisms. Such attention modules can be applied to prioritize relevant features, for example, hand motion overhead position.
- Further research can be conducted to examine hybrid methods by incorporating different models, for example, vision transformers and pretrained models. More efforts are required to explore how to leverage the advantages of different architectures to improve reliability and performance even further for applications involving ArSL recognition. This might entail fusing the attention mechanisms of vision transformers with features from pretrained models.
- Further research on simultaneous recognition of manual and non-manual signs, including facial expression, hand gestures, and body movement, in real-time with improved performance is encouraged. This can be achieved using fine-tuned deep learning techniques with a high configuration system to process the input data with minimal computational time.
- One possible direction for future advancement is the development of real-time mobile applications for ArSLR, as the majority of the work on ArSLRS is still in the research and prototype stages. Practical implementation issues that could arise for real-world applications include those related to computational capabilities, model deployment, real-time performance, and user friendliness. It is fair to state that, despite certain obstacles, the ArSLR community is steadily moving closer to creating real-time recognition systems that will eventually be used in everyday scenarios. Prior to that, it will be essential to improve performance consistency and remove a few common error places, where most algorithms often misinterpret a targeted sign.

#### 3.4.5. Limitations of the Study

This systematic review has some limitations. Firstly, this study only surveyed research papers published between 2014 and 2023, extracting them based on a predetermined keyword combination. Secondly, this review restricted the search of papers from only three online electronic databases, namely WoS, Scopus, and IEEE Explore. It is expected that many more papers on Arabic sign language recognition have been published in other academic journals. Thirdly, papers from conferences, editorials, prefaces, discussions, comments, tutorial summaries, workshop summaries, panels, and other non-journal publications are not included in this systematic review. Consequently, gray literature is not considered in this study.

## 4. Conclusions

Intelligent solutions for Arabic sign language recognition are still gaining interest from academic scholars thanks to recent developments in machine learning and deep learning techniques. This study presents a systematic review of ML/DL techniques utilized in ArSLR-relevant studies in the period between 2014 and 2023. Using data from 56 full-text research publications that were obtained from the Scopus, WoS, and IEEE Xplore online databases, an overview of the current trends in intelligent-based ArSL recognition is provided.

Thorough analysis of the dataset characteristics utilized in the reviewed papers was conducted. The datasets were grouped according to the recognition category they represent, whether it is fingerspelling, isolated words, continuous sentences, or a combination of them. The findings reveal that the most widely used dataset was ArSL2018, where it has been adopted by many fingerspelling recognition researchers since 2019. The analysis of the datasets shows that the area of ArSLR lacks high-quality, large-scale, publicly available datasets, particularly for isolated words and continuous recognition. Availability of such datasets would play a significant role in advancing this field and enable researchers to focus on improving recognition algorithms in order to boost performance and achieve high accuracy outcomes. The adoption of deep learning models—which are still being refined and will only gain more traction in the upcoming years—has been a major driving force behind recent advancements in this field. The past decade has seen the development of numerous unique and extremely creative ideas for ArSLR systems, such as feature extraction from sensor data or videos and passing them into neural classifiers.

In this study, I reviewed the state-of-the-art techniques for ArSLR tasks based on ML/DL algorithms, which have been developed over the last 10 years, and categorized them into groups according to the type of recognition: fingerspelling, isolated words, continuous sentences, and miscellaneous recognition. Due to their superior qualities, CNN-based algorithms are used in the most popular method to extract discriminative features from unprocessed input. Several different types of networks were frequently combined to increase overall performance. These models can handle data from a variety of sources and formats; they have been successfully applied to static images, depth, skeleton, and sequential data. Many researchers have shifted toward employing CNN-based transfer learning for ArSL recognition. When compared to conventional CNN-based deep learning models, the reviewed studies show that the transfer learning approach—which makes use of both pretrained models and vision transformers—achieved a greater accuracy. Even though the pretrained models outperformed the vision transformers in terms of accuracy, vision transformers demonstrate more consistent learning. Recent fingerspelling recognition studies were found to exploit ensemble methods, where several models are combined, seeking to increase the overall performance.

Ultimately, regardless of the advancements in research on ArSL recognition, there is still an apparent lack of practical applications and software for performing these tasks. In order to narrow the gap between research and practical implementation, it is necessary that accessible and user-friendly software and applications for ArSL recognition be developed. The development of trustworthy, usable, high-performance software solutions will help those who are hard of hearing or deaf, and it may enhance their daily interactions and communication in general.

## Figures and Tables

**Table 1:** Summary of existing surveys of Arabic sign language recognition (ArSLR).

Ref	Year	Journal/Conference	Timeline	SL	Datasets	Classifiers	Feature Extraction	Performance Metrics	Systematic	Focus of the Study	Source
[15]	2014	IEEE Transactions on Human-Machine Systems	Not specified	Arabic	Partial	Yes	Yes	Recognition accuracy: yes	No	Reviews systems and methods for image-based and sensor-based ArSLR, main challenges, and presents research directions for ArSLR.	WoS Scopus Google Scholar
[17]	2014	IEEE 11th International Multi-Conference on Systems, Signals & Devices (SSD14)	Not specified	Arabic	Partial	Yes	Yes	Recognition accuracy: yes, Error rate: partial	No	Reviews methods for image-based ArSLR, challenges, and shows research directions for ArSLR.	WoS Scopus Google Scholar
[9]	2020	Malaysian Journal of Computer Science	2001–2017	Arabic	Partial	Yes	Yes	Recognition accuracy: yes	No	Provides review, taxonomy, open challenges, research roadmap, and future directions.	WoS Scopus Google Scholar
[12]	2021	Bayburt University Journal of Science	Not specified	Arabic	No	Yes	Partial	Recognition accuracy: yes	No	Reviews vision-based ArSLR studies and shows challenges and future directions.	Google Scholar
[2]	2021	Archives of Computational Methods in Engineering	2007–2017	Arabic and others.	Partial	Yes	No	Recognition accuracy: yes	Yes	Review previous studies on recognition of different sign languages, including ArSLR, with a focus on six aspects: acquisition method, signing mode, single/double handed, static/dynamic signing, techniques used, and recognition rate.	Google Scholar
[20]	2021	Journal of Physics: Conference Series	2010–2019	Arabic and others	No	Yes	Yes	Recognition accuracy: yes	No	Gives an overview of the ArSLR studies in terms of input devices, feature extraction and classifier algorithms.	Google Scholar
[18]	2023	Conference of Mathematics, Applied Sciences, Information and Communication Technology	Not specified	Arabic, Iraqi sign language and others	No	Yes	No	Recognition accuracy: yes	No	Presents techniques used in sign language research and shows the main classification of the approaches.	Scopus
[19]	2023	International Symposium on Networks, Computers and Communications (ISNCC)	Not specified	Arabic	Partial: Only public datasets	No	No	No	No	Reviews the current ArSL datasets and how to improve them.	Scopus
[21]	2024	Indian Journal of Computer Science and Engineering (IJCSE)	Not specified	Arabic	Partial: Only public datasets	Yes	Yes	Accuracy	No	Investigates the ArSLR studies focusing on classifier algorithms, feature extraction, and input modalities. It also shed light on the public datasets in the field of ArSLR.	Google Scholar
Our	2024	Sensors Journal	2014–2023	Arabic	Yes	Yes	Yes	Yes	Systematic	A variety of ArSLR algorithms are systematically reviewed in order to determine their efficacy and potential for raising ArSLR performance. Furthermore, the present and future directions in the field of ArSLR are examined, emphasizing significant areas of innovation and interest.	

Yes means this aspect is fully covered, Partial means this aspect is partially covered, and No means this aspect is not covered.

**Table 2:** Research questions related to the used datasets.

No.	Research Question		Purpose
RQ.1	What are the characteristics of the datasets used in the selected papers?		To discover the quality and characteristics of the datasets utilized for ArSLR.
	RQ1.1	How many datasets are used in each of the selected papers?	
	RQ1.2	What is the availability status of the datasets in the reviewed papers?	
	RQ1.3	How many signers were employed to represent the signs in each dataset?	
	RQ1.4	How many samples are there in each dataset?	
	RQ1.5	How many signs are represented by each dataset?	
	RQ1.6	What is the number of datasets that include manual signs, non-manual signs, or both manual and non-manual signs?	
	RQ1.7	What are the data acquisition devices that were used to capture the data for ArSLR?	
	RQ1.8	What are the acquisition modalities used to capture the signs?	
	RQ1.9	Do the datasets contain images, videos, or others?	
	RQ1.10	What is the percentage of the datasets that represent alphabets, numbers, words, sentences, or a combination of these?	
	RQ1.11	What is the percentage of the datasets that have isolated, continuous, fingerspelling, or miscellaneous signing modes?	
	RQ1.12	What is the percentage of the datasets based on their data collection location/country?	

**Table 3:** Research questions related to the used algorithms and methods.

No.	Research Question		Purpose
RQ.2	What were the existing methodologies and techniques used in ArSLR?		To identify and compare the existing ML/DL methodologies and algorithms used in different phases of ArSLR.
	RQ2.1	Which preprocessing methods were utilized?	
	RQ2.2	Which segmentation methods were applied?	
	RQ2.3	Which feature extraction methods were used?	
	RQ2.4	What ML/DL algorithms were used for ArSLR?	
	RQ2.5	Which evaluation metrics were used to measure the performance of ArSLR algorithms?	
	RQ2.6	What are the performance results in terms of recognition accuracy?	

**Table 4:** Research questions related to the research limitations and future directions.

No.	Research Question		Purpose
RQ.3	What are the challenges, limitations, and future directions mentioned in the reviewed papers?		To identify and analyze limitations, challenges, and future directions in the field of ArSLR research.
	RQ3.1	Has the number of research papers regarding ArSLR been increasing in the past decade?	
	RQ3.4	What are the limitations and/or challenges faced by researchers in the field of ArSLR?	
	RQ3.5	What are the future directions for ArSLR research?	

**Table 13:** Reasons for utilizing more than one dataset in the reviewed studies.

No of Datasets	Ref.	Reason for Utilizing More Than One Dataset	Comments
2	[76]	Reason 2 & 3	Different modalities and sign words from different domains.
	[38]	Reason 1	Alphabet and words.
	[56]	Reason 4	Arabic and American alphabet sign language datasets.
	[71]	Reason 4	Arabic sign language for 32 letters, 11 numbers and none, and American sign language for 27 letters, delete, space, and nothing.
	[46]	Reason 5	Both datasets contain sign language representations for 14 letters. The second dataset is entirely used for testing.
	[47]		
	[62]	Reason 1	Alphabet and numbers.
3	[82]	Reason 1, 2, 3 and 4	Arabic sign language for words, Indian sign language for common and technical words, numbers and fingerspelling (alphabets), and Italian sign language for words. All datasets have different modalities.
	[96]	Reason 3	A total of 40 Arabic sign language sentences created from 80 words lexicon signs are captured in each dataset with different acquisition devices, namely DG5-VHand data gloves, two Polhemus G4 motion trackers, and camera.
	[90]	Reason 1, 2, 3 and 4	KArSL-502 and KArSL-190 constitute letters, numbers, and words captured using Kinect V2 and stored in different modalities, RGB, depth, and skeleton joint points. The third dataset is LSA64 (Argentinian Sign Language), which contains only words captured by the camera and saved as RGB.
	[63]	Reason 5	All the ArSL datasets are for letters, however, two of them are only used for testing.
4	[72]	Reason 4 & 5	All the ArSL datasets are for letters, however, only one of them is used for testing.
5	[78]	Reason 1, 2, 3, 4	KArSL-33, KArSL-100, and KArSL-190 constitute letters, numbers, and words captured using Kinect V2 and stored in different modalities, RGB, depth, and skeleton joint points. The fourth dataset, Kazakh-Russian sign language (K-RSL), contains sign language for Kazakh-Russian words captured by LOGITECH C920 HD PRO WEBCAM and saved as RGB, skeleton joint points. The fifth dataset is for greeting words captured by camera and saved as RGB.

**Table 14:** Summary of fingerspelling ArSLR research papers.

Year	Ref.	Preprocessing Methods	Segmentation Methods	Feature Extraction Methods	ArSLR Algorithm	Evaluation Metrics	Performance Results	Testing and Training Methodology	Signing Mode
2015	[42]	Transform to YCbCr, Image normalization.	Skin detection, background removal techniques.	Feature extraction through observation detection and then creation of observation vector	HMM. This algorithm divides the rectangle surrounding by the hand shape into zones.	Accuracy.	100% recognition accuracy for 16 zones.	Training: 70.87% (253 images), and Testing: 29.13% (104 images).	Signer-dependent
2015	[43]	-	-	SIFT to extract the features and LDA to reduce dimensions.	SVM, KNN, nearest-neighbor (minimum distance)	Accuracy.	SVM shows the best accuracy around 98.9%.	Different partitioning methods were experimented	Signer-dependent
2017	[48]	Remove observations with rows that had same values, and rows that had multiple missing values or null.	-	Two feature types: − Type 1: three angles for each hand bone (angles between the bone and the three axes of the coordinate system). − Type 2: one angle between each of the two bones.	SVM, KNN, and RF.	Accuracy, and AUC.	SVM produced a higher overall accuracy = 96.119%.	Training: 75% (1047 observations), Testing: 25% (351 observations).	Signer-dependent
2018	[49]	Transform the color images into gray level images.	Skin detection to extract the hand region from the background.	HOG, EHD, GLCM, DWT, and LBP.	One versus all SVM.	Precision, Recall, Accuracy.	Best accuracy was 63.5% for one ersus all SVM using HOG.	Not Mentioned	Signer-dependent
2019	[52]	− Resize images to fit each pretrained CNNs image input layer. − Image data augmentation.	-	AlexNet, SqueezeNet, VGGNet16, VGGNet19 GoogleNet, DenseNet, MobileNet and ResNet 18, ResNet50, ResNet101, InceptionV3	AlexNet, SqueezeNet, VGGNet16, VGGNet19 GoogleNet, DenseNet, MobileNet and ResNet 18, ResNet50, ResNet101, InceptionV3.	Accuracy, Error Rate, Sensitivity, Specificity, Precision, F1 Score, MCC, Kappa, confusion matrix.	ResNet18 achieved highest accuracy with 99.52%.	Training: 90% (48,644 images), Testing: 210% (5405 images)	Signer-dependent
2020	[53]	− Remove noise. − Grayscale conversion. − Resize each image to 64 × 64 pixels. − Normalization.	-	CNN	CNN	Accuracy, Loss.	Best accuracy obtained was 92.9%.	Training: 80% (43,239 images), Testing: 20% (10,810 images)	Signer-dependent
2020	[68]	− Resize images to 128 × 128 pixels. − Images conversion into a neutrosophic image. − Gaussian filter to deal with the noise.	Global thresholding technique.	The feature extraction for neutrosophic images by GLCM is based on pixel and their neighbors.	Clustering by Fuzzy c-means algorithm.	Accuracy.	Best accuracy obtained was 91%	Training: 80% (20,480 images), Testing: 20% (5120 images)	Signer-dependent
2020	[54]	− Random under-sampling to reduce the dataset imbalance. − Data augmentation.	-	Fine-tuned VGG16, and ResNet152.	Fine-tuned VGG16, and ResNet152.	Accuracy.	Best accuracy for VGG16 was 99.26% and for ResNet152 was **99.57%.**	After resampling: Training: 80% (20,480 images), Testing: 20% (5120 images).	Signer-dependent
2020	[69]	− Resize images to 128 × 128 RGB images. − Data augmentation.	-	CNN	CNN	Accuracy, Loss (categorical cross-entropy), confusion matrix.	Best accuracy obtained was 90%	Training: 80% (3100 images), Testing: 20% (775 images).	Signer-dependent
2020	[44]	− Resize images to 32 × 32 pixels. − Normalization.	-	Restricted Boltzmann machine (DBN-based) feature extraction.	DBN followed by SoftMax, and DBN followed by SVM.	Accuracy, Sensitivity, Specificity, Precision, F-1 measure, Error Rate.	Deep belief network (DBN) with a SoftMax achieved best accuracy of 83.32%	Training: 50%, Testing: 50%.	Signer-dependent
2020	[55]	− Transform images to grayscale 64 × 64 pixels. − Normalization. − Convert class labels to one-hot encoding vectors.	Deep CNN.	Deep CNN.	CNN with four convolutional layers, four max pooling layers, and five dropout layers.	Accuracy, training time and loss (categorical cross-entropy)	Best obtained accuracy was 97.6%.	Training: 60%, Validation: 20%, Testing: 20%.	Signer-dependent
2021	[72]	− Convert to grayscale image. − Resize to a 640 × 480-pixel image. − Apply filtering methods to remove noise.	Implement global threshold method, then, edge detection technology using a Sobel filter method. After that, implement hand edge detection.	Hand shape-based description.	C4.5, NB, and KNN and MLP.	Accuracy, confusion matrix, prediction scale (Kappa statistic) and RMSE.	KNN obtained the best accuracy of 99.5%.	Dataset 1: 100% training (2800 images), Dataset 2: 100% training (2800 images), Dataset 3: 100% training (1400 images), Dataset 4: 100% testing (1400 images).	Signer-independent
2021	[71]	− Resize images to 100 × 100. − Normalize images. − Image augmentation − The augmentation of data for the dynamic sign (noise salt and paper and blurring images with filters gaussian, median, averaging and morphological operation erosion and dilation of the dataset).	Single models: DenseNet121, VGG16, RESNet50, MobileNetV2, Xception, Efficient B0, NASNetMobile, and InceptionV3. Multi-models: DenseNet121 model and VGG16 model, RESNet50 & MobileNetV2, Xception&Efficient B0, NASNetMobile & InceptionV3, DenseNet121 & MobileNetV2, and DenseNet121&RESNet50.	Single models: DenseNet121, VGG16, RESNet50, MobileNetV2, Xception, Efficient B0, NASNetMobile, and InceptionV3. Multi-models: DenseNet121 model and VGG16 model, RESNet50 & MobileNetV2, Xception&Efficient B0, NASNetMobile & InceptionV3, DenseNet121 & MobileNetV2, and DenseNet121&RESNet50.	Single models: DenseNet121, VGG16, RESNet50, MobileNetV2, Xception, Efficient B0, NASNetMobile, and InceptionV3. Multi-models: DenseNet121 model and VGG16 model, RESNet50 & MobileNetV2, Xception&Efficient B0, NASNetMobile & InceptionV3, DenseNet121 & MobileNetV2, and DenseNet121&RESNet50.	Accuracy, precision, recall, F-1 measure, confusion matrix.	Single model: the DenseNet121 obtained the best accuracy with 100%. Multi-model: the DenseNet121 & VGG16 multi-model CNN is the best with accuracy = 100%.	Self-acquired dataset: Training: 80% (176,000 images), Validation: 10% (22,000 images), and Testing: 10% (22,000 images). ASL standard dataset: Training: 80% (69,600 images), Validation: 10% (8700 images), and Testing: 10% (8700 images).	Signer-dependent
2021	[73]	Resize images to 224 × 224.	Enhancing ROI pooling layer performance in VGG16 based on Faster R-CNN and ResNet18-based on Faster R-CNN.	− VGG16 based on Faster R-CNN, − ResNet18-based on Faster R-CNN.	− VGG16-Faster Region-based CNN (R-CNN), − ResNet18-Faster Region-based CNN (R-CNN).	Accuracy, precision, recall, F-1 measure, confusion matrix.	ResNet-18-Faster Region-based CNN (R-CNN) obtained higher accuracy with 93.4%.	Training: 60% (12,240 images), Validation: 20% (3060 images), Testing: 20% (3060 images).	Signer-dependent
2021	[57]	All images are normalized. Then, the images are standardized.	ArSL-CNN, ArSL-CNN + SMOTE, ArSL-CNN + RMU, and ArSL-CNN + RMO.	ArSL-CNN, ArSL-CNN + SMOTE, ArSL-CNN + RMU, and ArSL-CNN + RMO.	ArSL-CNN, ArSL-CNN + SMOTE, ArSL-CNN + RMU, and ArSL-CNN + RMO.	Accuracy, and confusion matrix.	ArSL-CNN + SMOTE obtained higher accuracy with 97.29%.	Training: 60%, Validation: 20%, Testing: 20%.	Signer-dependent
2021	[56]	− Data augmentation. − Normalization.	CNN-2 (two hidden layers), and CNN-3 (three hidden layers).	CNN-2 (two hidden layers), and CNN-3 (three hidden layers).	CNN-2 (two hidden layers), and CNN-3 (three hidden layers).	Accuracy, precision, recall, F-1 measure, confusion matrix.	CNN-2 produced the best results (accuracy of 96.4%) for the ArSL dataset. CNN-3 achieved an accuracy of 99.6% for the ASL dataset.	ArSL2018: Training: 49% (20,227 images), Validation: 21% (8669 images), Testing: 30% (12,480 images). ASL dataset: Training: 60% (20,283 images), Validation: 20% (7172 images), Testing: 20% (7172 images).	Signer-dependent
2022	[58]	Resize images.	ROI using experimented models	AlexNet, VGG16, ResNet50, and EfficientNet.	AlexNet, VGG16, ResNet50, and EfficientNet.	Accuracy, and loss (categorical cross-entropy).	AlexNet had the highest accuracy at 94.81%.	Training: 80%, Testing: 20%.	Signer-dependent
2022	[74]	− Reduce image size to 224 × 224 pixels. − Data augmentation.	Lightweight EfficientNet Models	Lightweight EfficientNet Models	Lightweight EfficientNet Models with different settings.	Accuracy, precision, recall, F-1 measure, confusion matrix, and loss (cross-entropy).	Lightweight EfficientNet model outperformed the other models, with accuracy of 94.30%.	Training: 80% (4320 images), Testing: 10% (540 images), Validation: 10% (540 images).	Signer-dependent
2022	[59]	Data augmentation.	Sobel operator method.	AlexNet, VGGNet and GoogleNet/Inception.	AlexNet, VGGNet and GoogleNet/Inception models.	Accuracy.	VGGNet was the best with 97%.	Training: 80%, Testing: 20%.	Signer-dependent
2022	[60]	− Resize images to 64 × 64 pixels. − Convert to RGB images. − Median filter for noise. − Data augmentation.	ResNet50 and MobileNetV2 together.	ResNet50 and MobileNetV2 together.	ResNet50 and MobileNetV2 together.	Accuracy, precision, recall, F1-score, confusion matrix.	Accuracy = 97.0%.	Training: 80%, Testing: 20%.	Signer-dependent
2022	[61]	− Remove class imbalance. − Resize images to 32 × 32 pixels. − Data augmentation. − Data normalization.	EfficientNetB4	EfficientNetB4	Xception, VGG16, Resnet50, InceptionV3, MobileNet and EfficientNetB4	Accuracy, precision, recall, F-score.	EfficientNetB4 had best accuracy with 95.0%	Training: 80%, Testing: 20%.	Signer-dependent
2023	[46]	− Convert images to 64 × 64 grayscale images. − Standardize Images.	Hand gesture detection using QSLRS-CNN model.	QSLRS-CNN model	100 Epochs: QSLRS-CNN, QSLRS-CNN with RMU, QSLRS-CNN with RMO, QSLRS-CNN with SMOTE.	Accuracy, recall, precision, F-score, confusion matrix. Training time.	Best result achieved by QSLRS-CNN with SMOTE (97.67%).	ArSL2018: Training: 80%, testing: 20%. ArSL dataset: Testing: 100%.	Signer-dependent
2023	[64]	-	− VGG, ResNet, MobileNet, Xception, Inception, DenseNet, InceptionResNet, BiT, and vision transformers (ViT & Swin). − CNNs.	− VGG, ResNet, MobileNet, Xception, Inception, DenseNet, InceptionResNet, BiT, and vision transformers (ViT & Swin). − CNNs.	− Transfer learning with pretrained models: VGG, ResNet, MobileNet, Xception, Inception, DenseNet, InceptionResNet, BiT, and vision transformers (ViT and Swin). − Deep learning using CNNs.	Accuracy, AUC, precision, recall, F1-score and loss	ResNet and InceptionResNet obtained a comparably high accuracy of 98%.	Not mentioned	Signer-dependent
2023	[65]	− Resize images to 64 × 64 pixels. − Image normalization. − Data augmentation.	Six pretrained fine-tuned models, VGG16, MobileNetV2, Xception, InceptionV3, ResNet50V2, ResNet152.	Six pretrained fine-tuned models, MobileNetV2, VGG16, InceptionV3, Xception, ResNet50V2, ResNet152.	Six distinct pretrained fine-tuned models, MobileNetV2, VGG16, InceptionV3, ResNet50V2, Xception, ResNet152.	Accuracy and Loss.	InceptionV3 and achieved 100% accuracy.	Training: 70%, Testing: 30%.	Signer-dependent
2023	[66]	− Resize Images to 64 × 64. − Rescale images to 32 × 32. − Data Augmentation.	CNN-based EfficientNetB3 with encoder and decoder network.	CNN-based EfficientNetB3 with encoder and decoder network.	CNN-based EfficientNetB3 with encoder and decoder network.	Accuracy, recall, precision, F1-score, confusion matrix, loss (cross-entropy).	Accuracy with encoder and decoder = 99.26% (Best accuracy).	70% training,20% validation and 10% testing	Signer-dependent
2023	[41]	Images are converted to 128 × 128 grayscale images.	-	− Individual descriptors: DWT, DT-CWT, HOG. − Combined descriptors: DWT + HOG, − DT-CWT + HOG.	Three variants of the ANN: PNN, RBNN, and MLP, and SVM and RF.	Accuracy, processing time	Best Accuracy was for DT-CWT + HOG + SVM (One-against-all) with 94.89%.	50% training, 50% testing	Signer-dependent
2023	[47]	Convert Images to 64 × 64 grayscale images. -Standardize images using 0–1-pixel values.	Hand gesture detection using QSLRS-CNN model	QSLRS-CNN model	− 100 Epochs: QSLRS-CNN, QSLRS-CNN-RMU, QSLRS-CNN-RMO, QSLRS-CNN-SMOTE. − 200 Epochs: QSLRS-CNN, QSLRS-CNN-RMU, QSLRS-CNN-RMO, QSLRS-CNN-SMOTE.	Accuracy, precision, recall, F-score, confusion matrix, Training time.	100 Epochs: QSLRS-CNN-SMOTE: 97.67%. 200 Epochs: QSLRS-CNN-SMOTE: 97.79%.	ArSL2018: Training: 80%, Testing: 20%. ArSL dataset: Testing: 100%.	Signer-dependent
2023	[62]	Convert images into HSV color space.	Hand edge detection recognize hand shapes based on detecting human skin colors and mathematical morphology techniques.	VGG16, InceptionV3, Xception, MobileNet, NASNetLarge, VGG19, InceptionResNetV2, DenseNet121, ResNet50, DenseNet169, DenseNet201, NASNetMobile.	Transfer learning based on the majority voting of the 12 model’s predictions: VGG16, VGG19, ResNet50, InceptionV3, Xception, InceptionResNetV2, MobileNet, DenseNet121, DenseNet169, DenseNet201, NASNetLarge, NASNetMobile.	Accuracy, recall, precision, F-score.	Best accuracy was for transfer learning CNN with majority voting = 93.7%	ArSL2018: Training: 90%, Validation: 10%. ASL-Digits-dataset: Training: 90%, Validation: 10%.	Signer-dependent
2023	[63]	− Resize images to 64 × 64. − Data augmentation.	VGG16, VGG19 fine tuning block4 and block5.	VGG16, VGG19 fine tuning block4 and block5.	VGG16, VGG19 fine tuning block4 and block5.	accuracy, top-5 accuracy, Loss	VGG-16, fine tuning block4 obtained best accuracy = 96.51%.	ArSL2018: Training: 70%, Testing: 30%. Self-built dataset: Testing: 100%. Ibn Zohr University dataset: Testing: 100%	Both

**Table 15:** Summary of isolated words ArSLR research papers.

Year	Ref.	Preprocessing Methods	Segmentation Methods	Feature Extraction Methods	ArSLR Algorithm	Evaluation Metrics	Performance Results	Testing and Training Methodology	Signing Mode
2015	[79]	Transform to YCbCr. Image normalization.	Skin detection, background removal techniques.	Face and Hand Isolation and feature extraction through observation detection and calculation using proposed EMCC.	EMCC and HMM.	Accuracy	Best accuracy = 98.8%	Training: 81.13% (1045 videos), and Testing: 18.87% (243 videos).	Signer-dependent
2015	[80]	PCNN signature to decrease the random noise and add image signature.	-	Leap motion sequences for hand signs. ANN is used with PCA to extract features from facial expressions and body movement.	ANN with MLP for hand signs recognition. ANN with PCA and MLP for facial expressions and body movement.	Accuracy	Facial expressions: 90%, body movement: 86%, hand sign: 90%, integrated sign testing: 95%	-	Signer-dependent
2017	[81]	Remove all the null values and any features with zero variance.	-	Two histograms to show two types of features: Type1: contains 3-angles for each hand joint. Type2: is for one angle between two vectors.	SVM with default parameters and linear kernel (SVMLD), SVM with tuned parameters and linear kernel (SVMLT), SVM with default parameters and radial kernel (SVMRD), and SVM with tuned parameters and radial kernel (SVMRT).	Accuracy	SVMLD obtained best accuracy with 97.059%.	Training: 75% (109), Testing: 25% (34)	Signer-dependent
2018	[82]	− For hand shapes, the RGB image conversion to greyscale. − For hand motion, normalize three-dimensional skeleton joint coordinates.	The hand shape segmentation is based on the depth and position of the hand joints (thresholding).	− Hand shape descriptor using HOG and dimensionality reduction using PCA. − Motion+ descriptor: 3D skeleton data and Cov3DJ+ descriptor.	CCA for hand shape matching and RF.	Accuracy, and confusion matrix.	The proposed algorithm achieves an accuracy of 55.57%.	Training: 55.99% (7754 gestures), validation: 24.28% (3362 gestures), and testing: 19.80% (2742 gestures).	Signer-independent
2018	[5]	-	− Dynamic skin detector based on face skin tone color to segment hands. − A skin-blob tracking to track hands.	Geometric features of the spatial domain are used.	Euclidean distance	Accuracy, confusion matrix.	Recognition accuracy = 97%.	Training: 66.67% (300 videos), Testing: 33.33% (600 videos).	Signer-independent
2019	[83]	-	-	GMM, and LDA.	Results from: GMM and LDA were combined individually using DS theory.	Accuracy	Accuracy = 91.83%	Training: 70% (1400 samples), Testing: 30% (600 samples).	Signer-dependent
2019	[33]	Resize segmented key frames (images) into 201 × 201 pixels.	− Video segmentation: Key Frames Extraction (KFE) and Shot Boundary Detection (SBD) algorithms. − Video key frames: RoI.	Intensity histogram and GLCM feature vectors.	Weighted Euclidean distance measure	Accuracy	System accuracy = 95.80%	-	Signer-dependent
2019	[76]	-	Segmenting videos to key frames (VidSeg), and KeyFeat algorithm to detect hands (optical flow and thresholding).	MFT, LBP, HOGs, and HOG-HOF. Compare different techniques on Database-01.	HMM using GRT toolkit. K-means clustering algorithm to quantize the features vector.	Accuracy and overlap ratio.	The best accuracy is achieved by HMM with MFT and HOG features with 99.11% and 99.33%.	Database-01: not stated, Database-02: Testing 100%.	Signer-independent
2020	[84]	-	-	Feature vectors extracted by LMC.	LSTM	Accuracy, precision	One hand:89%, two hands: 96%.	Training: 80% (352, 232 for one hand & 120 for two hands), Testing: 20% (88, 58 for one hand, 30 for two hands).	Signer-dependent
2020	[85]	Data interpolation to equalize the number of captured frames for the same gesture word.	-	Data interpolation in feature extraction (bone directions and joint angles were the main features).	Different settings of 3 algorithms (RF, SVM, KNN), KNND, KNNT, RFD, RFDS, RFT, RFTS, SVMLD, SVMLT, SVMRD, SVMRT, SVMRTS.	LogLoss, AUC, Accuracy.	The best accuracy was achieved by SVMRTS with 83%.	Not mentioned	Signer-dependent
2020	[77]	-	DeepLabv3	Hand shape features: CSOM. The sequence of feature vectors: BiLSTM.	BiLSTM	Accuracy, confusion matrix.	Accuracy: 89.5%	Training: 70%, Testing: 30%.	Signer-independent
2021	[70]	− Low-pass filter to eliminate dynamic noise, − High-pass filter to remove low-frequency drift from gyroscope readings.	An adaptive segmentation method to measure the energy of the signal and then compare with two thresholds.	8 TD features, and 9 features obtained from autocorrelation and FD, normalized arrays for both the DFT and the autocorrelation.	SVM and NB.	Accuracy	SVM, feature-based fusion. User-dependent: 98.6%, User-independent: 96%.	Training: 75% (3675), and Testing: 25% (1225).	Both
2021	[88]	-	CNN-LSTM, Inception-LSTM, Xception-LSTM, ResNet50-LSTM, VGG-16-LSTM, MobileNet-LSTM.	CNN-LSTM, Inception-LSTM, Xception-LSTM, ResNet50-LSTM, VGG-16-LSTM, MobileNet-LSTM. Signer-dependent mode: color and depth images are used, in signer-independent mode: optical flow is used.	CNN-LSTM, Inception-LSTM, Xception-LSTM, ResNet50-LSTM, VGG-16-LSTM, MobileNet-LSTM.	Accuracy	MobileNet-LSTM with transfer learning and fine tuning with 99.7% and 72.4% for signer-dependent and signer-independent modes.	The videos from three signers were divided to: 90% training, and 10% validation set. Testing: fourth signer’s videos.	Both
2022	[91]	Weighted average filtering approach.	CapsNet	CapsNet feature extractor to produce a collection of feature vectors.	Atom Search Optimization (ASO) with the Deep Convolutional Autoencoder (DCAE).	Accuracy, precision, recall, F1-score, Jaccard Index, confusion matrix, precision–recall analysis, Loss, ROC.	Accuracy of ASODCAE-SLR model = 99.17%	Training: 70%, Testing: 30%.	Signer-dependent
2022	[92]	− Video split into consecutive frames, − Cropping. − Normalization. − Resize all frames to 200 × 200 pixels. − Data augmentation.	− Single model: ResNet50-LSTM, Conv2D-LSTM, Conv3D-LSTM, Conv3D. − Multi-model: ResNet50-LSTM, DenseNet121-LSTM, ResNet50-GRU, MobileNet-LSTM, VGG16-LSTM, ResNet50-BiLSTM-Normalization.	− Single model: ResNet50-LSTM, Conv2D-LSTM, Conv3D-LSTM, Conv3D. − Multi-model: ResNet50-LSTM, DenseNet121-LSTM, ResNet50-GRU, MobileNet-LSTM, VGG16-LSTM, ResNet50-BiLSTM-Normalization.	− Single model: ResNet50-LSTM, Conv2D-LSTM, Conv3D-LSTM, Conv3D. − Multi-model: ResNet50-LSTM, DenseNet121-LSTM, ResNet50-GRU, MobileNet-LSTM, VGG16-LSTM, ResNet50-BiLSTM-Normalization.	accuracy, confusion matrix.	In single model: ResNet50-LSTM achieved highest accuracy with 99.62%. In multimodel, ResNet50-BiLSTM-Normalization obtained best accuracy with 100.0%.	Training: 85.7% (6300 videos), validation: 7.14% (525 videos), and testing: 7.14% (525 videos).	Signer-dependent
2022	[78]	Raw colored video frames and AFD frames (computed from absolute frame difference to detect changes and track objects between consecutive frames).	CNN and LSTM	CNN to extract features from each video frame, and LSTM to learn the temporal features across video frames.	Vanilla LSTM, CNN-LSTM, CNN-SLSTM (stacked LSTM), and CNN-SLSTM-FC (stacked LSTM and Fully Connected layer).	Accuracy, precision, recall, F1 score, and computation (training) time.	CNN-SLSTM (stacked LSTM) for raw colored video frames input achieved higher accuracy for all datasets with 95.7% for K-RSL, 100% for Shanableh, 99% for KArSL-100, and 99.4% for KArSL-190.	-	Signer-dependent
2022	[90]	The median filter to remove the outliers.	DMN–(VGG16, Xception, ResNet152V2, and MobileNet)-LSTM, AMN—(FWD, BWD, Bi), SRN—(FWD-SRN, BWD-SRN, Bi-SRN).	DMN—(VGG16, Xception, ResNet152V2, and MobileNet)-LSTM, AMN—(FWD, BWD, Bi), SRN—(FWD-SRN, BWD-SRN, Bi-SRN).	DMN—(VGG16, Xception, ResNet152V2, and MobileNet)-LSTM, AMN—(FWD, BWD, Bi), SRN—(FWD-SRN, BWD-SRN, Bi-SRN).	Accuracy	Signer-dependent mode: DMN-MobileNet obtained high accuracy = 99.1%. Signer-independent mode: Bi-AMN obtained high accuracy = 91.8% in	KArSL-190: 80% training, 20% testing. KArSL-502: 80% training, 20% testing. LSA64: 80% training, 20% testing.	Both
2023	[93]	DenseNet169 model	DenseNet169 model	DenseNet169 model	Deer Hunting Optimization with Machine Learning (ASLGC-DHOML), and MLP.	Accuracy, sensitivity, specificity, F-score, G-measure, precision-recall curve, ROC curve analysis and loss.	Accuracy = 92.88%	-	Signer-dependent
2023	[35]	− Preprocessed video data to 20 frames. − Resize video frames to 224 × 224 pixels.	MediaPipe Holistic was used for hand-face region segmentation (ROI).	CNN-LSTM-SelfMLP with MobileNetV2 and ResNet18 backbones for feature extraction.	MobileNetV2, ResNet18, CNN, LSTM, SelfMLP.	Accuracy, Specificity, F1-score, Precision, Recall, ROC curve analysis, loss, confusion matrix, standard error, and confidence interval.	MobileNetV2-LSTM-SelfMLP achieved the best accuracy of 87.69%	The videos from three signers were split in two: 90% for training, & 10% for validation. Testing: videos for the fourth signer.	Signer-independent
2023	[94]	− Reduce each frame’s dimensions. − Convert into grayscale. − Apply the difference function to every two consecutive frames.	Adaptive threshold on the (n − 1) frames.	double CNNs.	CNN, and RNN.	Accuracy, loss, confusion Metrics, and top-1 Accuracy.	Accuracy = 92	-	Signer-independent

**Table 16:** Summary of continuous ArSLR research papers.

Year	Ref.	Preprocessing Methods	Segmentation Methods	Feature Extraction Methods	ArSLR Algorithm	Evaluation Metrics	Performance Results	Testing and Training Methodology	Signing Mode
2015	[95]	Resampling techniques to reduce data size. Normalize and standardize readings (Z-score).	Manual video segmentation and labelling.	Window-based approach	MKNN	Word recognition rate, Sentence recognition rate	Sentence recognition rate = 98.9%	Training: 70% (280), Testing: 30% (120).	Signer-dependent
2018	[97]	Data normalization (position and user size).	Automatic segmentation to separate between consequent signs.	Automated feature selection for the joints (hands, shoulder, elbow, wrist, spin mid and head center).	(KNN, SVM, ANN) with and without majority voting, and DTW.	Accuracy, response time.	Best accuracy is 89% for KNN classifier with majority voting and the segmentation accuracy reached 91%.	Training: 66.67% (840 samples), Testing: 33.33% (420 samples).	Signer-independent
2019	[96]	-	Manual labeling and segmentation in vision-based SLR. In sensor-based SLR, synchronize camera with gloves and tracker recordings to detect boundaries.	Vision-based dataset: 2D DCT, zonal coding. Sensor-based datasets: Sliding window-based statistical features extraction techniques.	MKNN, and HMM.	Word recognition rate, sentence recognition rate, computation time (train time & classification time)	Sentence recognition rates: MKNN achieved the best results for all datasets (97.78% for the gloves dataset). Word recognition, HMM was the best with 99.20% for the glove’s dataset.	DB1 (gloves): Training: 70% (280), and Testing: 30% (120), DB2 (Tracker): Signer-dependent, Training: 70%, Testing: 30%, Signer-independent, Training & Testing: 50%, DB3 (vision-based): not stated.	Signer-dependent for all datasets except for vision-based dataset 2 signer-dependent and signer-independent
2023	[98]	-	Divide videos into motion images equal to the number of sentence words.	Pertained CNN Inception-v3 network.	LSTM, biLSTM, biLSTM×2, biLSTM×3.	Word recognition rate, sentence recognition rate, training time and testing time.	biLSTM×2 achieved best results with word recognition rate = 97.3, and sentence recognition rate = 92.6	-	Signer-dependent

**Table 17:** Summary of miscellaneous ArSLR Research Papers.

Year	Ref.	Preprocessing Methods	Segmentation Methods	Feature Extraction Methods	ArSLR Algorithm	Evaluation Metrics	Performance Results	Testing and Training Methodology	Signing Mode
2017	[37]	− Background compensation for objects, infrared scanning to construct a 3D representation, and digital image creation of the hand in real time.	The palm speed during the motion is used as segmenter, to recognize the continuous sentences.	The used features in the model were Palm-Features set and Bone-Features set.	Static gestures: SVM with poly kernel and RBF, KNN and ANN with a Multilayer Perceptron. Dynamic gestures: classification with Simple Majority and DTW.	Accuracy	Static gestures: KNN was the best with 99% for palm features and 98% bone features. Dynamic gestures: DTW was the best with 97.4% for palm features and 96.4% for bone features.	Training: 66.67%, Testing: 33.33%.	Signer-independent
2020	[38]	Crop out hand object, resize images to 64 × 64 pixels, convert to grayscale image, reduce noise by Gaussian filter and median blur, and data augmentation.	CNN with 4 hidden layers.	CNN with 4 hidden layers.	− Train and test CNN with 4 hidden layers on ArSL2018 dataset. − Apply the trained CNN and ontology on the Self-acquired words dataset.	Accuracy	Accuracy on ArSL2018 is 88.87%, and accuracy on the word’s dataset is 94.31%.	ArSL2018: Training: 80% (42,960), Testing: 20% (11,089). Words dataset: Training: 70% (200), Testing: 30% (88)	Signer-dependent
2021	[39]	-	2D CNN network (OPENPOSE Network (OPL))	2D CNN network (OPENPOSE Network (OPL)).	Concatenation in serial of two parallel networks, a 2D CNN (OPL) network for key-points estimation and a second 1D CNN skeleton network.	Accuracy, precision, recall, F1- measure, Confusion matrix.	Signer-dependent mode: 98.39% for dynamic signs and 88.89% for static signs. Signer-independent mode: 96.69% for dynamic signs and 86.34% for static signs.	Training: 60% (9600 videos), Validation: 20% (3200 videos), Testing: 20% (3200 videos).	Both

**Table 18:** Limitations and future work for fingerspelling ArSLR studies.

Year	Ref.	Limitations/Challenges	Future Work
2015	[42]	Not mentioned	Not mentioned
2015	[43]	Not mentioned	− Improve the results in case of image occlusion. − Increase the size of the dataset. − Recognize alphabets from video frames and real time ArSL system.
2017	[48]	Not mentioned	Address how the proposed prototype can be utilized to collect and classify dynamic sign gestures of one word or phrase.
2018	[49]	Not mentioned	− Investigate kernel SVM to improve the performance of the proposed method. − A relevance feature weight is assigned to each sign gesture.
2019	[52]	The required training time.	− Implement a low depth residual network to reduce the training time. − Develop a fully automated system for ArSLR system.
2020	[53]	Not mentioned	Not mentioned
2020	[68]	Not mentioned	− Evaluate the proposed system on new datasets. − Test it in a real-time recognition.
2020	[54]	Not mentioned	Not mentioned
2020	[69]	Not mentioned	− More advanced hand gestures recognizing devices can be considered such as Leap Motion or Xbox Kinect. − Increase the size of the dataset and publish it.
2020	[44]	Not mentioned	− Investigate the impact of normalization and whitening on feature extraction. − Investigate the sparsity factor with various parameters. − Increase the dataset size. − Add more images that were underrepresented in feature extraction and have similar gestures, to investigate their effect on the process of learning and classification.
2020	[55]	− Limitation of the dataset size and number of signers used in obtaining the dataset. − Limitation in the system hardware available as image processing and deep learning algorithms require high processing and memory requirements. − Limitation of achieving acceptable recognition within reasonable time and accuracy.	− Extend the proposed solution by developing a system to translate the words and sentences. − Build effective algorithms to achieve higher accuracy. − Increase the dataset size. − Develop real-time mobile application for Arabic sign language translation.
2021	[72]	Not mentioned	− Develop educational tools for deaf and dumb children using the proposed AArSLRS. − Provide translation systems for the meanings of the Holy Quran.
2021	[71]	Not mentioned	− Implement mobile-based application to recognize Arabic sign language in real-time. − Use dynamic gesture recognition for Arabic sign language. − Build video-based dataset.
2021	[73]	Not mentioned	Study the performance of YOLO algorithm instead of Faster R-CNN for ArSL letter recognition.
2021	[57]	Not mentioned	− Consider testing the ArSL-CNN on different datasets. − Study the effectiveness of RNN for the application. − Utilize transfer learning for ArSLR model.
2021	[56]	Not mentioned	− Study CNN-2 and CNN-3 architectures on larger datasets. − Consider time and space complexity optimization to enable these architectures to be used on mobile phones.
2022	[58]	− The proposed model is limited to detecting only one object (a hand) without taking the background into consideration, which would affect the performance. − The detection process in the proposed model is highly sensitive to variations in the hand’s pose.	− Build a mobile application based on the proposed model. − Exploit transfer learning. − Other sign language datasets such as the American Sign Language dataset, MS-ASL can be used to pre-train a model to utilize transfer learning. − Implement data augmentation to produce training samples.
2022	[74]	Not mentioned	− Investigate utilizing transformers. − Extend the proposed work to recognize the Arabic sign language words or common expressions.
2022	[59]	Not mentioned	Generate real-time sentences and videos using sign language based on CNN models.
2022	[60]	Limitations in Real-Time recognition.	Not mentioned
2022	[61]	Not mentioned	− Combine different transfer learning models for single-hand gesture recognition, such as MobileNet and ResNet50 architectures. − Apply these models to recognize the two-hand gestures.
2023	[46]	− The proposed model is limited to images of static gestures that show the discontinuous letters at the beginning of the Qur’anic Surahs.	− Test the proposed model, QSLRS-CNN on various datasets and − Evaluate RNN and LSTM. − Improve the proposed model by adopting transfer learning. − Develop a deep learning model to translate the Holy Qur’an meanings into sign language.
2023	[64]	− The dataset was not representative enough. − Real-world applications may require solutions to practical implementation issues such computational resources, model deployment, real-time performance, and user usability.	− Consider addressing the class imbalance found in ArSL datasets to guarantee an equal representation and enhance the accuracy of minority classes. − Expand the study to include video-based ArSL recognition. − Investigate hybrid models that combine vision transformers and pretrained models to enhance accuracy. − Examine further the optimization methods and fine-tuning approaches for transfer learning using pretrained models and vision transformers. − Examine methods for data augmentation that are especially designed for ArSL recognition. − Look into cross-language transfer learning. − Carry out benchmarking and comparing various pretrained models, architectures, and vision transformers on ArSL recognition tasks and evaluate how well they perform on bigger and more varied datasets.
2023	[65]	Not mentioned	Not mentioned
2023	[66]	Not mentioned	Lower the learning parameters and model size using quantization or model-pronening techniques to boost the model’s efficiency.
2023	[41]	− The system is not robust to variations in illumination. − Only in the case where there is no background clutter, the HOG descriptor efficiently captures the hand structure. Thus, for practical real-life applications, an accurate segmentation is necessary. − Real-time applications will consider the 1.2 s characterization time to be slow. − The Random Forest classifier takes a lot of trees to perform well, which makes the model slower.	− Enhance the proposed system by including segmentation and hand tracking phases for real-time acquisition and recognition. − The system needs to be enhanced in order to attain high accuracy, particularly when dealing with a complicated background.
2023	[47]	− The proposed model exhibits the discontinuous letters at the beginning of Qur’anic surahs using just static gestures.	− Examine RNN and LSTM. − Using many datasets when testing the QSLRS-CNN. − Apply transfer learning to create a better deep learning model for ArSL that is compatible with ArSL variations. − Develop a deep learning model to translate the meanings of the Holy Qur’an into sign language.
2023	[62]	Not mentioned	− Investigate more cutting-edge deep-learning techniques to enhance the model’s practicality and accuracy. − Evaluate the model’s resilience and scalability for additional sign languages.
2023	[63]	− Because training was done on a dataset of remarkably similar images, testing on the other two datasets revealed low recognition accuracy. − The training time was impacted by the Internet speed.	− Continue the research to look into further deep learning models, such ResNet. − Further understanding of the decision-making processes of the model’s layers is necessary for the recognition process.

**Table 19:** Limitations and future work for isolated ArSLR studies.

Year	Ref.	Limitations/Challenges	Future Work
2015	[79]	Not mentioned	Not mentioned
2015	[80]	The suggested semantic oriented post-processing module that detects and corrects any translation errors would perform well in a particular predefined field.	By including a semantic-oriented post-processing module to identify and fix any translation errors, the accuracy of the translation can be improved.
2017	[81]	Since the dataset utilized in the study is small, the outcomes of using a large dataset with the linear and radial kernels may change.	− Makes use of depth sensors and supervised machine learning to identify ArSL phrases while considering the LMC’s constrained workspace and the user’s motion. − For Kinect and LMC to collect and recognize gestures more accurately and instantly, they both need to be faster.
2018	[82]	Not mentioned	Not mentioned
2018	[5]	Not mentioned	Not mentioned
2019	[83]	Not mentioned	− Examine various scenarios in which LMCs could be combined with other sensors, − Expand the system to incorporate continuous Arabic signs. − Incorporate efficient techniques to decrease the processing complexity. − Develop an upgrade plan for the system to enable mobile platform deployment.
2019	[33]	Not mentioned	− Increase the number of non-manual features for full ArSL recognition. − Increase the number of signs in the dataset and combine classifiers for a higher recognition rate. − Focus on continuous dynamic gesture (sentence) translation and recognition.
2019	[76]	Not mentioned	Not mentioned
2020	[84]	Not mentioned	Provide a mechanism that can transform sign language movements into complete sentences while recognizing overlapped gestures.
2020	[85]	Not mentioned	− Work on implementing algorithms that can yield better accuracy rates, including deep learning algorithms. − Increase the number of observations in the dataset.
2020	[77]	Not mentioned	Not mentioned
2021	[70]	Not mentioned	− Glove design: Bluetooth IMU sensors can be utilized, together with 3D printed rings that allow the sensors to be positioned on the fingers. − Preprocessing: employing more sophisticated methods such sensor fusion (e.g., Kalman filter) − Feature Extraction: merging magnetometer data with ACC and GYRO features to detect magnetic north. − Dataset: expand the dataset by gathering the Arabic Deaf people’s most commonly used words or gestures. − Classification: utilizing sequence-based classification techniques such as HMM and RNN.
2021	[88]	Not mentioned	− There will be further examination of the effectiveness of each component of the non-manual gestures. − Examine alternative deep learning methods for distinguishing between isolated signs. − Discover how the suggested methods may be applied to different datasets of signs. − Focus on ArSL continuous sign language recognition.
2022	[91]	Not mentioned	− Expand the suggested model to recognize sign boards in real-time applications. − A real-time, large-scale dataset can be used to evaluate the performance of the proposed model. − To improve the SL recognition performance, a combination of DL models can be developed.
2022	[92]	Not mentioned	Not mentioned
2022	[78]	Not mentioned	− Alternative models and datasets can be employed for comparison. − Further study is needed to extend the suggested method for continuous sign language recognition and − Enhance the training time of the proposed model.
2022	[90]	− In the LSA64 dataset, signs are performed by nonexpert signers. − Differences in gestures represented by the various signers of the sign. − The enormous number of produced frames, particularly when sign gestures are captured at high frame rates.	− Transformers and the attention mechanism are two other models that can be utilized to recognize sign language. − It is also possible to employ alternative sign language recognition modalities.
2023	[93]	Not mentioned	Employing sophisticated DL classification models would enhance the ASLGC-DHOML model’s performance.
2023	[35]	There are only a few subjects and classes in the dataset used to classify sign language.	− Build a larger dataset with sign alphabets, numbers, words, and sentences from various signers, as well as variations in background, lighting, and camera angles. − Create a sign language transformer that uses MediaPipe Holistic’s landmark data rather than videos as input. − Include the attention mechanism in the model for Arabic sign video classification so that the model can be trained on bigger datasets and be capable of recognizing sign videos in real-life scenarios. − Incorporate more cutting-edge 1D and 2D CNNs into a real-time Arabic Sign Language Transformer.
2023	[94]	Not mentioned	− Include additional users and new signs in the proposed dataset. − Focus on phrases as opposed to words. − Adjust the suggested model to accommodate the new videos through incorporating grammatically correct sentences. − Employing various strategies, networks, architectures and techniques. − More research can be undertaken with more Arabic datasets.

**Table 20:** Limitations and future work for continuous ArSLR studies.

Year	Ref.	Limitations/Challenges	Future Work
2015	[95]	Not mentioned	Not mentioned
2018	[97]	Not mentioned	− Utilize alternative techniques in the recognition phase, such as DTW for the purpose of improving the proposed system. − The segmentation method can be improved for increased accuracy. − Further testing of more words from various domains will improve the proposed system and increase its ubiquity.
2019	[96]	Not mentioned	Not mentioned
2023	[98]	For sign language recognition in the real world, the suggested solution is unsuitable.	Minimize the computation time and make the proposed solution appropriate for real-time sign language recognition.

**Table 21:** Limitations and future work for miscellaneous ArSLR studies.

Year	Ref.	Limitations/Challenges	Future Work
2017	[37]	Not mentioned	Enhance recognition accuracy by utilizing deep learning with large samples of complete sentences and incorporating more features engineering.
2020	[38]	Not mentioned	− Improve deep learning with ontology to address dynamic real video in real-time applications. − Transform the system into a mobile application.
2021	[39]	− Each camera’s field of vision was excessively wide, capturing the entire scene. This resulted in two thirds of worthless side pixel information. − Some singers had trouble coordinating their hands and making the same gestures. − The research focused on the initial, less-than-ideal factory calibration parameters of both Kinect cameras. − Owing to some blurry pixels in the original frames, finger key points were not produced correctly, primarily for the transient frames.	− To determine whether a shape is a sign or a transitory gesture, add a sign boundary detector or a network to the proposed solution. − Group the produced frame key points into meaningful reduced clusters would be the subject of further research in order to make the deep model lighter and more compact on mobile devices. − Improve delay removal across the pipeline using convolution suppression and optimum data propagation to minimize network size and maximize classification performance. − Additional enhancements include the ability to zoom in on singers and the addition of an automated method for detecting palm positions.

**Table 22:** Comparison of the most popular ML/DL techniques in ArSLR research.

ML/DL Methods	Recognition Accuracy	Efficiency	Robustness	Comments
SVM	Tends to be high, >85, especially with small datasets.	Moderate depending on kernel.	Tends to be high, which makes it good for noisy data.	Performs well with high-dimensional data but can be computationally expensive with large datasets or non-linear kernels.
KNN	Moderate to high, depending on metrics.	Low (distance computation-heavy). Fast to train; slow during testing.	Low to Moderate (sensitive to noise)	Simple to implement but computationally intensive for large datasets; performance depends heavily on the choice of k and distance metric.
HMM	Very high > 95%	Highly efficient in sequential data.	Moderate in handling sequence noise.	Excels in temporal sequence recognition but requires feature-rich sequential data.
MKNN	Very high > 95%	Moderate	Moderate	It addresses KNN limitations but remains computationally intensive for large datasets.
RF	Moderate to high	High	High robustness to noise and outliers.	Highly robust and efficient, which makes it ideal for large feature sets with proper tuning.
NB	Moderate	High	Low. It assumes feature independence. Sensitive to noisy, incomplete, or irrelevant features.	Fast and efficient, but makes strong assumptions (e.g., feature independence), which are rarely true in ArSLR applications.
CNNs	High for static signs > 90%.	Moderate (requires substantial computational resources for training but is efficient during inference).	High (handles noise and variations in gesture images effectively).	Excellent for static gesture recognition but requires a large dataset for optimal performance.
RNNs	High for dynamic gestures > 90%	Moderate (sequential processing can be time-consuming, especially for long sequences).	High.	Suitable for sequential gesture recognition, therefore it is commonly used for recognizing continuous gestures in dynamic sequences.
Hybrid CNN-RNN	High for both tasks > 90%	Low to Moderate (training is computationally expensive due to hybrid architecture).	Very high (effective for spatial-temporal data).	Powerful for complex gestures combining static and dynamic gestures but requires significant hardware resources.
Transfer Learning	Very high when fine-tuned on ArSLR datasets.	Faster training as it requires less data due to pre-learned features. However, the efficiency can be moderate depending on architecture depth.	Robust to variations in input data.	Benefits from the generalization capabilities of large-scale pretrained models.
Transformer-based Models	Very high > 95%.	Low. Requires significant computational power.	Very High (captures complex spatial-temporal dependencies).	State-of-the-art but requires extensive computational resources and large datasets.

## Data Availability

Data are contained within the article.
